# Supplementary figures and images for: Ethnic disparities in initiation and intensification of diabetes treatment in adults with type 2 diabetes in the UK, 1990–2017: A cohort study
Source: PLoS Med. 2020 May 15;17(5):e1003106. doi: 10.1371/journal.pmed.1003106 (PMC7228040; doi:10.1371/journal.pmed.1003106)

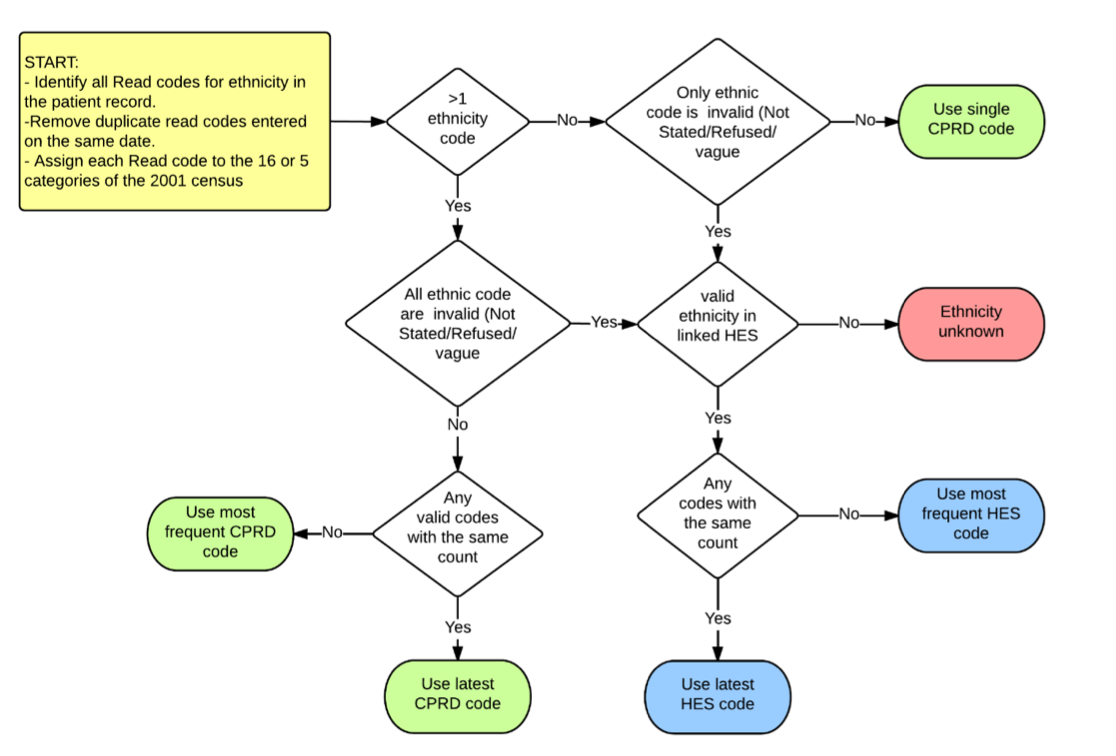

Supplement: S1 Fig — (TIFF) [file pmed.1003106.s002.tiff]

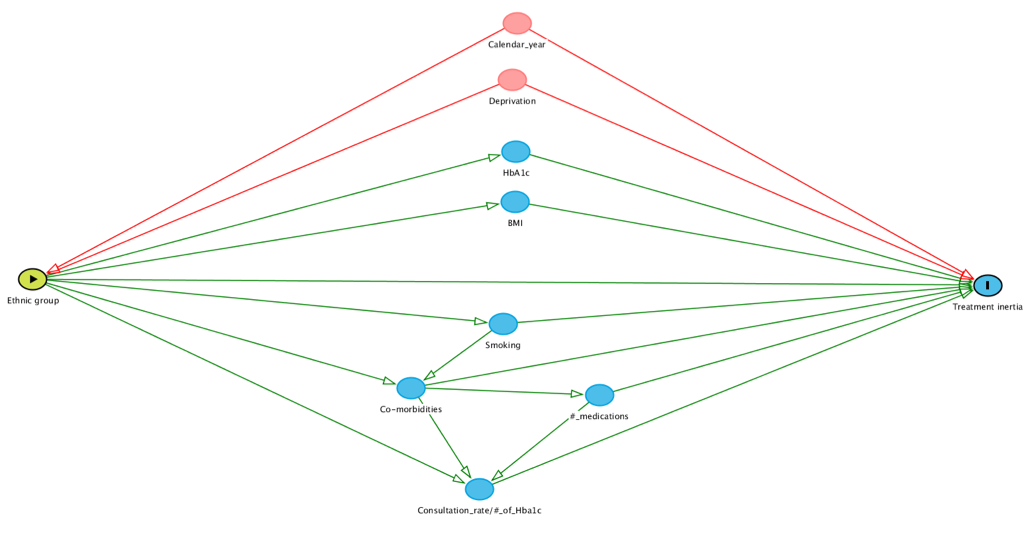

Supplement: S2 Fig — (TIFF) [file pmed.1003106.s003.tiff]

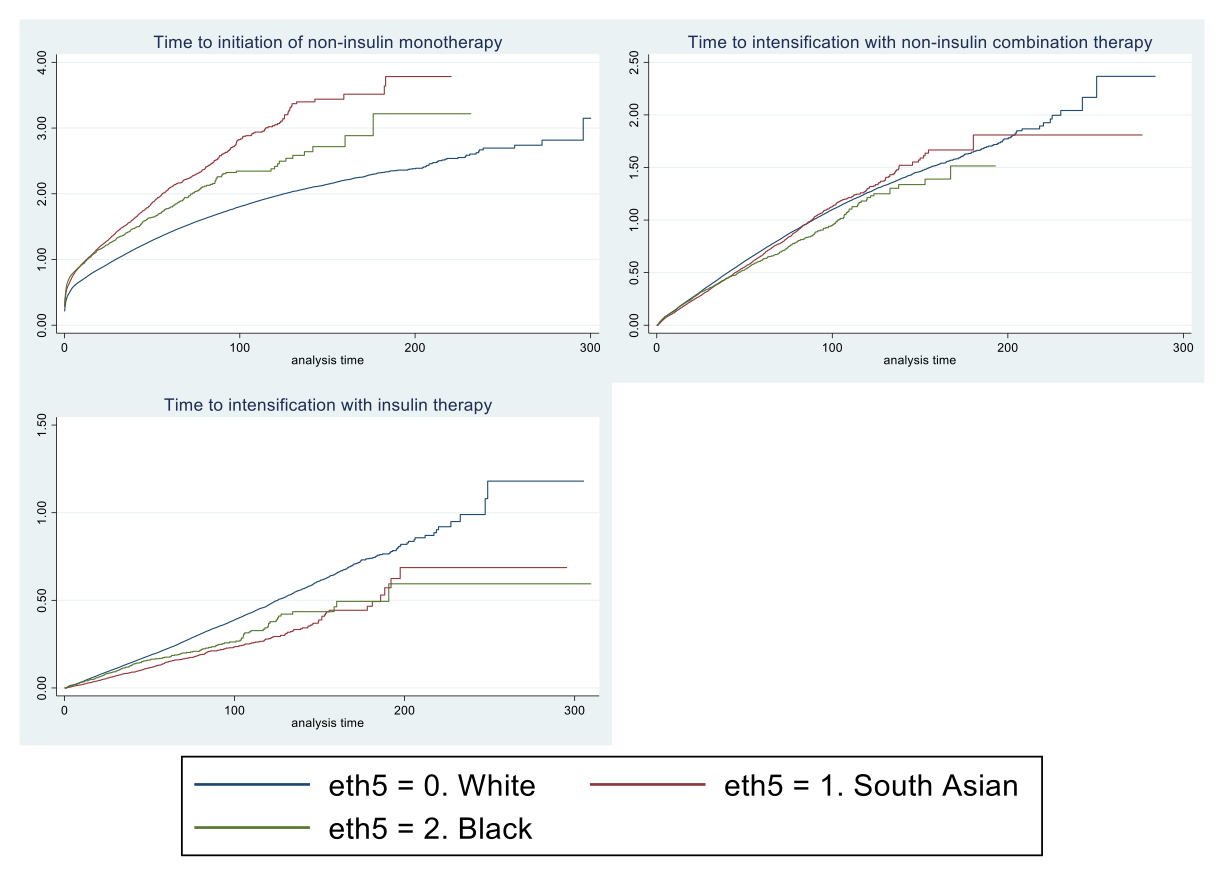

Supplement: S3 Fig — (TIFF) [file pmed.1003106.s004.tiff]

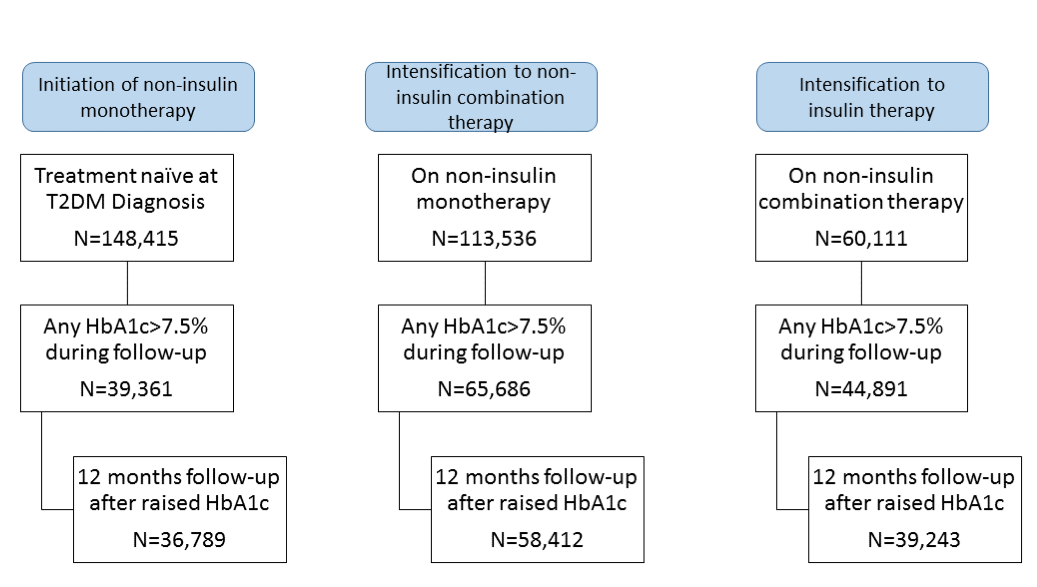

Supplement: S4 Fig — (TIFF) [file pmed.1003106.s005.tiff]
