# Supplementary material for: Ethnic disparities in initiation and intensification of diabetes treatment in adults with type 2 diabetes in the UK, 1990–2017: A cohort study
Source: PLoS Med. 2020 May 15;17(5):e1003106. doi: 10.1371/journal.pmed.1003106 (PMC7228040; doi:10.1371/journal.pmed.1003106)
Supplement: S1 Text — ISAC, Independent Scientific Advisory Committee. (DOCX) [file pmed.1003106.s006.docx]

**Supplementary File S4: ISAC Scientific Protocol**

| **Applicants must complete all sections listed below**  **Sections which do not apply should be completed as ‘*Not Applicable’*** |
| --- |
| 1. **Study Title^§^**   **^§^***Please note:* *This information will be published on CPRD’s website as part of its transparency policy*  Ethnic inequalities in trajectories of cardio-metabolic risk factor control and outcomes of type two diabetes |
| 1. **Lay Summary (Max. 200 words)^§^**   **^§^***Please note:* *This information will be published on CPRD’s website as part of its transparency policy*  People with diabetes are at increased risk of developing microvascular disease (which damages the eyes, kidneys, and nerves) and cardiovascular disease (such as heart disease, heart attacks, and stroke). We know that there are significant differences in the prevalence of diabetes, and in the risk of these major disease outcomes between different ethnic groups. What we do not know, is where along the care pathway these differences stem from. The aim of this observational GP database study is to identify areas of healthcare which can be modified in order to reduce the risk of future disease amongst people with type 2 diabetes. I will firstly explore whether individuals from different ethnic groups are equally likely to visit their GP and have high levels of risk factors identified. Secondly, amongst patients identified as being at high risk for heart and blood vessel disease, I will examine whether medications for controlling blood pressure, blood sugar, and cholesterol are prescribed equitably between ethnic groups. Finally I will examine whether the benefits of these treatments reduce the risk of disease equally between ethnic groups. This will establish an evidence base for future clinical trials and guidelines for the management of diabetes tailored to the multi-ethnic UK population. |
| 1. **Technical Summary (Max. 200 words**)**^§^**   **^§^***Please note:* *This information will be published on CPRD’s website as part of its transparency policy*  Type 2 diabetes increases the risk of vascular disease, with co-morbid hypertension and hyperlipidaemia increasing risk further. UK studies have identified substantial ethnic differences in the risk of vascular outcomes amongst individuals with type 2 diabetes. Whether these inequalities stem from differences in healthcare usage, quality of care, or differences in treatment efficacy remains unknown. Pharmacological control of blood pressure and blood glucose has been shown to profoundly reduce vascular risk. However, to date, no study has adequately examined whether these benefits manifest equally across different ethnic groups.  The aim of this study is to identify clinical factors underlying ethnic inequalities in vascular outcomes of type 2 diabetes in order to generate an evidence base for clinical management of diabetes tailored to the UK population.  Firstly, using multilevel linear and logistic regression, ethnic differences in the access to health care and the identification of high cardiovascular risk will be quantified. Secondly, using logistic regression, amongst individuals identified as being at high risk, ethnic differences in the prescription of appropriate and timely antidiabetic, antihypertensive, and lipid lowering treatment will be determined. Thirdly, amongst patients prescribed appropriately, ethnic differences in trajectories of risk factor control following treatment initiation and time taken to achieve control to target by ethnic group will be compared using latent class growth curve modelling. Finally, the causal relationship between pharmacological treatment and major vascular outcomes will be compared between ethnic groups using survival analysis incorporating marginal structural models. |
| 1. **Objectives, Specific Aims and Rationale**   **The aim of this study** **is**: To establish the role of clinical factors underlying ethnic differences in vascular disease amongst people with type 2 diabetes, by determining the extent to which inequalities in rates of consultations, quality of preventive care, and efficacy of pharmacological treatment contribute to ethnic inequalities in diabetic outcomes. The findings will be used to highlight areas where a formal trial would be beneficial, and ultimately, inform the evidence base for the clinical management of diabetes in the diverse UK population.  **The objectives are:**  1. To identify individual and practice level determinants of ethnic differences in use of health services (defined using consultation rates) and detection of high cardio-metabolic risk for patients newly diagnosed with type 2 diabetes.  2. To examine whether patients identified as having uncontrolled cardio-metabolic risk factors are treated equitably with antihypertensive, lipid lowering, and glucose lowering treatment across ethnic groups, and whether ethnic specific guidelines for blood pressure control are adhered to.  3. To determine whether antihypertensive, antidiabetic and lipid lowering treatment results in adequate and timely control of blood pressure, glycaemia, and cardiovascular risk across all ethnic groups.  4. To determine whether the benefits of pharmacological treatment on reduction of microvascular (retinopathy, nephropathy, neuropathy) and macrovascular (coronary heart disease, stroke, myocardial infarction, heart failure) events manifest equally across ethnic groups.  **Rationale:**  Large clinical trials, such as The UK Prospective Diabetes Study (UKPDS) and The Collaborative Atorvastatin Diabetes Study (CARDS), have confirmed the benefits of intensive glycaemic, lipid and antihypertensive control on a range of micro- and macrovascular outcomes.(1–5) However, to date, no study has adequately examined whether the benefits manifest equally across different ethnic groups.  Understanding how best to reduce vascular events and related mortality in diabetic populations is both timely and necessary. Given that risk factors are distributed inequitably across ethnic groups, and the overall disease burden is higher in ethnic minority populations, minimizing inequalities will have a large impact on the absolute reduction of disease burden and mortality in the UK. |
| 1. Background   Individuals with type 2 diabetes are at increased risk of vascular disease compared to those without, with the presence of co-morbid hypertension and hyperlipidaemia increasing risk further(6,7) Blood pressure, lipid, and glucose lowering agents have a profound beneficial effect on reducing risk.(1,8) Globally, the prevalence of type 2 diabetes is 3-6 times higher in South Asian and Black African/Caribbean populations compared to White populations.(9–11) Furthermore, for a given level of risk factor, people of ethnic minority groups may have worse outcomes than the White population.(12–15)  Marked ethnic differences in the risk of vascular outcomes among people with diabetes have been established in UK populations.(16–20) Whether these inequalities stem from differences in healthcare usage, quality of diabetes management, or differences in treatment efficacy remains unknown. Though equity of service provision is a central tenet of the National Health Service(21), recent studies have highlighted ethnic differences in access to healthcare, treatment provision and risk factor control. (22–30)  Though ethnicity has now been incorporated into care guidelines, such as those for hypertension and diabetes, there is still much scope to extend this to a wider range of conditions known to be ethnically patterned.(31,32) NICE acknowledges in its existing guidance that further research into ethnic differences is necessary in order for it to better tailor its recommendations to the UK population.(33,34) Research using electronic health databases will be able to provide much of the evidence base for treatment and care guidelines.  Understanding how best to reduce vascular events and related mortality in diabetic populations is both timely and necessary. Given that risk factors are distributed inequitably across ethnic groups, and the overall disease burden is higher in ethnic minority populations, minimizing inequalities will have a large impact on the absolute reduction of disease burden and mortality in the UK. |
| 1. **Study Type** Descriptive and Hypothesis testing |
| 1. **Study Design** An observational prospective cohort study using CPRD data and linked HES/ONS/IMD data. |
| 1. **Feasibility counts**   My PhD has demonstrated ethnic distributions in the CPRD are comparable to the 2011 census, with 90% completeness of ethnicity recording for patients registered since 2010.(35) In August 2013, 372,805 patients with diagnosed type 2 diabetes in the CPRD, were identified via an adjudication algorithm which takes into account Read codes, prescribing, blood sugar levels, ethnicity, and age. In the July 2016 database, this number has increased to 400,086, of whom 243,448 are eligible for linkage with HES and ONS mortality data. |
| 1. **Sample size considerations**   The July 2016 CPRD was used to conduct the following sample size calculations:  Objective 1a) Identify ethnic differences in GP consultation rate  Null Hypothesis: Consultation rate is equal across ethnic groups. From QRESEARCH: The average patient had 3.9 consultations each year in 1995 rising to 5.5 consultations each year by 2008.(36) From THIN: Average annual consultation date for diabetic patients was 11.6 in 2011. The minimum sample size to detect a difference in annual consultation rate of 0.1 consultation/year with 80% power was calculated using the command power 11.6 11.7, sd1(0.5) sd2(0.5).  Objective 1b) Identify ethnic differences in risk factor monitoring  Null Hypothesis: The proportion of patients receiving preventive care is equal across ethnic groups. Research using the Lambeth Datanet found that ethnic differences in BP monitoring for patients with diabetes ranged from 95.6% for White, to 93.1% for South Asian, 93.7% for Caribbean and 97.1% for African groups.(26) The minimum sample size to detect a 1% difference in proportions with 80% power was calculated using the command: power twoproportions 0.94 0.95.  Objective 2) Identify ethnic differences in appropriate pharmacological treatment  Null Hypothesis: The proportion of patients receiving appropriate pharmacological treatment is equal across ethnic groups. The minimum sample size to detect a 2% difference in proportions with 80% power was calculated using the command: power twoproportions 0.94 0.95.  Objective 3) Identify ethnic differences in proportion of patients achieving appropriate risk factor control following treatment initiation  Null Hypothesis: The proportion of patients achieving appropriate risk factor control is equal across ethnic groups. The minimum sample size to detect a 2% difference in proportions with 80% power was calculated using the command: power twoproportions 0.94 0.95.  Objective 4) Identify ethnic differences in time to first vascular events  Null Hypothesis: The time taken to experience ones first incident vascular event following diabetes diagnosis is equal across ethnic groups. In order to detect a difference in of 0.2 in the hazard ratio between ethnic groups with 80% power, the command: stpower cox, hratio(1.2) was used  Within the population of 400K patients with type 2 diabetes in the July 2016 CPRD, 150,972 are of White ethnicity, 15,348 are of South Asian ethnicity, and 7,096 are of Black African/Caribbean ethnicity. Amongst the 243,448 eligible for HES and ONS linkage, 101,324 are of White ethnicity, 11,616 are of South Asian ethnicity, and 5,656 are of Black African/Caribbean ethnicity. The study population includes enough people from each ethnic group to identify the minimally clinically relevant difference for each study objective.  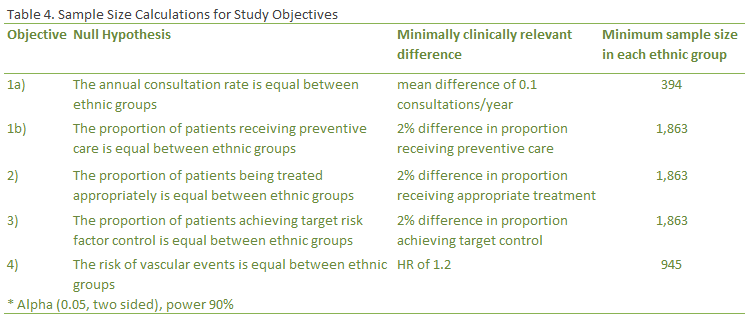 |
| 1. **Data Linkage Required (if applicable):^§^**   **^§^***Please note that the data linkage/s requested in research protocols will be published by the CPRD as part of its transparency policy*  Linkage to HES inpatient and outpatient data, ONS mortality data, and Individual IMD score is requested.  Linkage to HES inpatient data will allow for improved identification of all conditions of interest (Myocardial Infarction, CKD, CHD, heart failure, neuropathy, stroke, and retinopathy) both at baseline to identify disease free cohorts, and in follow-up to identify ethnic differences in risk of each developing outcome.  Linkage to HES outpatient data will be contribute to the analysis of ethnic differences in consultation and attendance rates for diabetes related care not provided in the primary care setting (for example, various clinics for retinopathy screening and dialysis)  ONS mortality data will be used to identify cause-specific mortality for each of the conditions listed above. In the survival analysis, both fatal and non-fatal disease incidence will be considered. Death due to all other causes will be used to identify end of follow-up up for patients not experiencing the outcome of interest.  Individual level IMD score will be used to separate the effects of ethnicity and depressionrivation on diabetes care and outcomes. |
| 1. **Study population**   All patients aged 18 and over with newly diagnosed type 2 diabetes between 1990 and 2017 in the CPRD eligible for the linkages requested. For the final objectives examining risk of incident vascular disease, individuals with established disease prior to diabetes onset will be excluded from the analysis of that particular outcome. |
| 1. **Selection of comparison group(s) or controls**   Ethnicity will be defined according to self-report as coded on the patient record using the 9i% or 9S% hierarchy. Ethnicity codes will be collapsed into the 16 categories of the 2011 Census for England and Wales. Discrepant and multiple ethnicity codes will be adjudicated according to an algorithm to identify the most common or most recent ethnicity code available. For patients with missing ethnicity in CPRD, ethnicity will be defined according to the “ethnos” variable in the linked HES record. As linked HES data are only available from 1997 onwards, the completeness of the ethnicity variable is likely to be lower for patients registered only in the period 1990-1997 compared to patients whose registration period includes time from 1997 onwards. Sensitivity analyses restricted to the period of full linkage (1997-2016) will be conducted to examine the effect of differential ethnicity recording, and identify potential bias in the resulting effect estimates for between group comparisons.    Patients of all ethnic groups will be included in the study, however testing of main hypotheses will be restricted to people of White, South Asian, and Black ethnicity. |
| 1. **Exposures, Health Outcomes^§^ and Covariates**   **^§^***Please note:* *Summary information on health outcomes (as included on the ISAC application form above )will be published on CPRD’s website as part of its transparency policy*  Exposures: Within the study population of all individuals with newly diagnosed type 2 diabetes between 1990 and 2017, the key study exposure will be self-reported ethnicity as recorded in the primary care record or in the linked HES record.  Outcomes: The outcome of interest will vary according to each objective.  Objective 1a: Consultation rates in primary care and attendance rates in hospital  Objective 1b: Time updated measures of blood pressure, HbA1c, and cardiovascular risk assessment (QRISK2) and time taken to achieve target control.  Objective 2: Timely and appropriate prescribing and intensification of antihypertensive, antidiabetic, and lipid lowering medications based on preceding risk factor levels.  Objective 3: Trajectories of control of blood pressure, hba1c, and cardiovascular risk.  Objective 4: Vascular outcomes (Myocardial Infarction Nephropathy (CKD) Coronary Heart Disease Heart Failure Neuropathy Stroke Retinopathy)    Covariates for all analyses will include age, sex, depressionrivation according to individual IMD score, presence of vascular disease at baseline, medication use at baseline, calendar year (to account for temporal changes in disease management guidelines and recommended treatment regimes), GP practice identifier (for clustering in multilevel models), BMI, smoking status, family history of diabetes or CVD. |
| 1. **Data/ Statistical Analysis**   **Follow-up period: will begin at the latest of: one year after current registration, the practice up to standard date, date of diabetes diagnosis, or start of linkage to HES/ONS data. The end of follow-up will be censored earliest of either transfer out date, death date, last collection date from practice, end of linkage to HES, or end of linkage to ONS mortality data. For Objective 4 of the study, follow-up will end at the earliest of all previously mentioned events, or date of first incident fatal or non-fatal vascular event** **Objective 1: Ethnic differences in GP consultation rate**  **A prospective cohort study including all patients diagnosed with type 2 diabetes between 1990 and 2017 in the CPRD, free from pre-existing vascular conditions will be conducted.** Ethnic differences in the annual GP consultation rate and the extent to which they are explained by individual and general practice level factors will be compared using **multilevel linear regression,** which nests patients within practices**.**  Linear regression model:  Population: All patients with type 2 diabetes  Outcome: number of GP consultations in the 12 months prior to diabetes diagnosis  Primary exposure: Ethnic group  Covariates: Baseline age, sex, depressionrivation, smoking status, vascular disease  Multilevel factors: GP practice  **Objective 1b: Time updated measures of blood pressure, HbA1c, and cardiovascular risk assessment (QRISK2 Read Codes 38DF and 38DP) and time taken to achieve target control**  Ethnic differences in the timeliness and completeness of blood pressure and HbA1c recording and cardiovascular risk assessment will be quantified using **multilevel logistic regression.** The extent to which ethnic differences are explained by individual and general practice level factors will be assessed.  Logistic regression model:  Population: All patients with type 2 diabetes  Outcome: Risk factor recorded within 12 months of diabetes diagnosis.  Primary exposure: Ethnic group  Covariates: Baseline age, sex, depressionrivation, smoking status, vascular disease, baseline consultation rate (from objective 1), GP practice list size, proportion of non-white patients registered at the GP practice.  Multilevel factors: GP practice identifier  Broadly speaking, measures will be considered to have been recorded in a timely manner if they appear on the GP record a) Within 6 months of diagnosis (prior or after, as the information may have formed part of the diagnostic decision making process) b) At least once per year following initial diabetes diagnosis.  **Blood Glucose**  Timeliness of blood glucose management will be considered in relation to the NICE guidance for type 2 diabetes. HbA1c measurements will be considered to have been recorded in a timely manner if they are recoded in either  a) 3-6 monthly intervals until HbA1c is stable  b) 6 monthly intervals once HbA1c is stable.  Similarly, prescribing for blood glucose management will be considered timely if an anti-diabetic medication is prescribed within 6 months of patient having a raised HbA1c value recorded (>48 mmol/mol or 6.5%).  For patients whose HbA1c rises above 58 mmo/mol (7.5%), intensification of prescribing will be considered timely if second or third line medications are prescribed within 6 months of the first recorded measure at this level.  **Blood Pressure**  As per the NICE guidance for type 2 diabetes, Blood pressure measures will be considered complete if they are recorded at least once every 12 months. For individuals with raised blood pressure, BP measurements will be considered timely if they are made every 1-2 months. Specifically, if BP recording is >150/90 mmHg, a repeat measurement will be considered timely if it is made within 1 month. If a BP recording of >140/80 mmHg (or 130/80mmHg for those with vascular disease), a repeat measurement will be considered timely if it is made within 2 months.  Antihypertensive prescribing will be considered timely if prescribed within 4-6 months of having a raised BP value measured. Intensification of antihypertensive therapy will be considered timely if the patients BP has remained uncontrolled for the previous 4-6 months using first line treatement only (ACE inhibitor or CCB if of Black African/Caribbean ethnicity).  **Other clinical measures**  Timeliness of other clinical measures (BMI, cardiovascular risk assessment, diabetes annual review, diabetes process of care measures) will be considered timely if they have been measured within 6 months of diagnosis (either before or after, as the measurement may form part of the diagnostic decision making process), and then at least once every 12 months thereafter.  **Ethnicity**  Completeness will be operationalized for ethnicity as ever having had a valid ethnicity code recorded (a code which is not “unknown” or at too high a level to be meaningful). **Objective 2: Ethnic differences treatment provision** a) Amongst diabetic patients without diagnosed hypertension, the odds of being prescribed appropriate antihypertensive treatment following a blood pressure reading ≥140/80 mmHg (or 130/80 mmHg if there is kidney, eye or cerebrovascular damage) will be determined using **multilevel** **logistic regression**.(37) Values outside of the valid range will be excluded (<=30 or >=200 for diastolic BP, <=40 or >=240 for systolic BP)  Currently, NICE specifies that patients of Black African/Caribbean ethnicity and those aged 55 and over should receive calcium channel blockers (CCB) or diuretics as first line monotherapy instead of angiotensin converting enzyme inhibitors (ACE/ARB).(38) The contribution of further individual and general practice level factors will be explored.  Logistic regression model:  Population: All patients with type 2 diabetes free from diagnosed hypertension at baseline/ not on antihypertensive medication at baseline, who have a BP reading of reading ≥140/80 mmHg or 130/80 mmHg during follow-up  Outcome: Appropriate antihypertensive medication prescribed within 6 months of first elevated BP reading (ACE/ARB or CCB or diuretic depressionending on ethnicity)  Primary exposure: Ethnic group  Covariates: Baseline age, sex, depressionrivation, smoking status, vascular disease baseline consultation rate, GP practice list size, proportion of non-white patients registered at the GP practice.  Multilevel factors: GP practice identifier  b) The odds of being prescribed metformin following initial diagnosis or first HbA1c measurement of ≥6.5%/47.5 mmol/mol and ≤20%/210 mmol/mol will be compared between ethnic groups using logistic regression. Amongst patients with an HbA1c level of ≥7.5%/58.5 mmol/mol) following initial metformin therapy, the odds of having treatment intensified to metformin plus sulfonylurea and/or insulin, as recommended in the NICE guidance (39) will be compared between ethnic groups using logistic regression.  Logistic regression model:  Populations:  a) All patients who have an HbA1c ≥6.5%/47.5 mmol/mol during follow-up  a) All patients who have an HbA1c ≥7.5%/58.5 mmol/mol following initiation of metformin  Outcome:  a) Initiation of metformin within 6 months of diagnosis  b) Intensification of diabetes therapy following metformin monotherapy within 6 months of having HbA1c ≥7.5%/  Primary exposure: Ethnic group  Covariates: Baseline age, sex, depressionrivation, smoking status, vascular disease baseline consultation rate, GP practice list size, proportion of non-white patients registered at the GP practice.  Multilevel factors: GP practice identifier  c) Amongst diabetic patients with a 10-year cardiovascular risk score of >10% according to the QRISK2 algorithm, the odds of being prescribed lipid lowering medications (statins) will be compared between ethnic groups using logistic regression.  Logistic regression model:  Population: All patients with type 2 diabetes not already on lipid lowering treatment prior to study entry.  Outcome: Initiation of lipid lowering medication within 6 months of having a QRISK2 score >10%  Primary exposure: Ethnic group  Covariates: Baseline age, sex, depressionrivation, smoking status, vascular disease baseline consultation rate, GP practice list size, proportion of non-white patients registered at the GP practice.  Multilevel factors: GP practice identifier Objective 3: Ethnic differences in risk factor control **latent class growth curve modelling** as demonstrated by Walraven et al. will be used **to identify ethnic differences in** trajectories of risk factor control following treatment initiation.(40,41) This method will identify subgroups of patients with distinct trajectories of HbA1c and blood pressure control, and statin persistence **(Figure 3). Five risk factor control groups will be derived from the latent class modelling. 1) Consistently good risk factor control 2) consistently poor risk factor control 3) Fast responders 4) Slow responders 5) Variable control.**  **Latent class model:**  **Population: All patients initiated on antihypertensive, antidiabetic , or lipid lowering medication (3 analyses)**  **Outcome: Derivation of 5 latent classes**  **Exposure: Repeated measures of BP, HbA1c, and QRISK2 score**  **Covariates: Baseline age, sex, depressionrivation, smoking status, vascular disease baseline consultation rate, GP practice list size, proportion of non-white patients registered at the GP practice.**  **Multilevel factors: GP practice identifier**  Multinomial logistic regression **will be used to explore whether people from different ethnic groups are more likely to belong to different risk factor control groups. Patients will be classified according to the five risk factor control groups derived from the latent class modelling. The odds of belonging to each control group will be compared between ethnic groups.**  **Multinomial logistic regression:**  **Population: All patients initiated on antihypertensive, antidiabetic , or lipid lowering medication (3 analyses)**  **Outcome: Probability of membership to each latent class**  **Primary exposure: Ethnic group**  **Covariates: Baseline age, sex, depressionrivation, smoking status, vascular disease baseline consultation rate, GP practice list size, proportion of non-white patients registered at the GP practice.**  **Multilevel factors: GP practice identifier**  Time to event analysis **will be used to compare the time taken to achieve control to target by ethnic group. Follow-up time will end at the earliest of reaching the target risk factor level, or the censoring criteria specified above.**  **Cox proportional hazards regression:**  **Population: All patients with type 2 diabetes**  **Outcome: Time to achievement of target risk factor level according to NICE guidelines (3 analyses)**  **Exposure: Ethnic group**  **Covariates: Baseline age, sex, depressionrivation, smoking status, vascular disease baseline consultation rate, GP practice list size, proportion of non-white patients registered at the GP practice.**  **Multilevel factors: GP practice identifier** Objective 4: Ethnic differences in treatment efficacy **The relationship between risk factor control and treatment is cyclical by nature, with baseline risk factor values influencing future treatment decisions which in turn influence subsequent risk factor levels. In order to best describe ethnic differences in the relationship between pharmacological treatment and major vascular outcomes, This can lead to the problem of time depressionendent confounding in which risk factors (such as HbA1c) that are affected by prior treatment. In order to overcome this problem, marginal Structural Models (MSMs) will be used to estimate the causal relationship between pharmacological treatment and major vascular outcomes and to compare the efficacy of treatment between ethnic groups while taking into account the time depressionendent nature of the exposure and covariates. MSMs offer a way to model the unbiased relationship between treatment and outcomes in an observational setting. (42,43) The analysis will account for the time varying nature of confounders such as HbA1c, cholesterol, blood pressure, BMI and smoking status.**  **Marginal Structural Models**  **Population: All patients with type 2 diabetes free from each vascular outcome of interest at baseline**  **Outcome: first ever fatal or non-fatal vascular disease endpoint**  **Exposure: Time updated medication use with ethnic group as an interaction variable.**  **Covariates: Baseline age, sex, depressionrivation, smoking status, time updated medication use, time updated risk factor level baseline consultation rate, GP practice list size, proportion of non-white patients registered at the GP practice.** |
| Plan for addressing confounding  Treatment status: **Databases contain information on prescriptions issued, but not whether they are dispensed, thus we may underestimate treatment effects. A comparison of prescribing data from electronic health databases and NHS dispensing data shows the two sources to be highly comparable, with 97% of endocrine and cardiovascular medications dispensed as prescribed.(44)**  Confounding by indication: **In routine care, patients who receive therapy likely differ from those who do not for reasons related to the outcome of interest. Propensity score matching will be used to match patients of different ethnic groups on different treatment regimens as closely as possible to minimize this source of bias. Marginal Structural Models will be used to minimize bias from time updating confounders.**  A priori confounders: **A priori confounders influencing ethnic differences in the patterning of macro- and micro-vascular outcomes amongst people with type 2 diabetes, available in the CPRD will be accounted for using stratification (by age and gender) and adjustment (for age, gender, depressionrivation, BMI, smoking status, GP practice, relevant cardio-metabolic co-morbidities, and relevant polypharmacy/contraindications).** |
| 1. **Plans for addressing missing data**   The analysis is restricted to individuals in the CPRD with type 2 diabetes. This is a population that will require ongoing chronic disease management and thus is likely to consult more regularly, and have key risk factors, process of care measures, and relevant diagnoses coded more accurately than the general population. Previous studies in other UK primary care databases have found the recording of ethnicity to approach 90% for individuals on chronic disease registers.(45)The recording of key variables such as diabetes diagnosis, ethnicity, and macro- and micro-vascular disease, and process of care measures/risk factors has improved since the introduction of the Quality and Outcomes Framework in 2004.  For the purposes of this study, multiple imputation is likely to be inappropriate because several of the variables included in the study are likely to be missing not at random, and because reasons for ’missingness’ are likely to differ depressionending on the covariate of interest. While measures such blood pressure, Hba1c, cardiovascular risk and BMI are likely to be measured regularly as part of a the incentivised diabetes care programme under the Quality and Outcomes Framework, other non-incentivised covariates are likely to be measured more frequently in individuals with established or suspected health conditions, limiting the ability to make accurate comparisons between those with and without disease. Furthermore, variables such as tobacco consumption and alcohol use are subject to reporting bias which may affect an individual’s propensity to visit the GP or to report habits candidly, most often leading to an underestimate of the true exposure. The appropriateness of methods such as multiple imputation will be explored to address the problem of missing data, particularly in the time period 1990-2004.If the missing data are considered missing at random, then multiple imputation can be utilized. If a variable is thought to be missing not at random, a complete case analysis will be performed and the likely limitations of this discussed.  Sensitivity analyses will be conducted to quantify the influence of missing data on resulting estimates by restricting to  a) Patients with complete covariate data (complete case analysis)  b) Patients registered from 2006 onwards when ethnicity completeness is highest |
| 1. **Patient or user group involvement (if applicable)**   Feedback from the BHF Heart Voices group has usefully informed the lay summary. During the study, I will have two aspects of patient group involvement.  **Involvement**  1. **Research Prioritization Workshop**  I will liaise with INVOLVE, UCL Partners and Diabetes UK invite patients and public to attend a consultation event.  a) I will present information about the key objectives, outputs and timelines.  b) Participants will discuss key issues for patients living with diabetes, identify priority areas for the research, and identify ways to sustain collaboration throughout the project.  c) Participants will be invited to join an advisory panel to meet regularly throughout the project and/or to receive updates via social media.  **Engagement**  1. **Reflection Workshop:**  a) I will facilitate a roundtable discussion on how to interpret and contextualize the research findings, draw out key themes, and incorporate them into recommendations for clinicians, policy makers and commissioners. |
| 1. **Plans for disseminating and communicating study results, including the presence or absence of any restrictions on the extent and timing of publication**   Study results will be disseminated via publication in peer-reviewed journals, and posters and presentations at conferences for the International Society of Pharmacoepidemiology, Diabetes UK, and the Royal College of General Practitioners.  I will create information leaflets summarizing key findings and learning points for circulation at general practices, community centres, diabetes support groups, and places of worship. |
| 1. **Limitations of the study design, data sources, and analytic methods**   The main limitation in this project is one common to all studies using electronic medical records. It is the problem of accurately defining measures for outcomes, exposures and covariates. Coding may be a reflection of individual physicians’ diagnostic beliefs (both GP and hospital doctors) and the patterns and context of coding behaviour (of GPs and those coding discharge letters). Variation in coding practices will impact on the reliability of the definitions we use to identify outcomes and covariates in our study. Research suggests that most diagnoses within CPRD are recorded accurately, and, further research suggests that there have been improvements in data quality in the domains assessed by the Quality and Outcomes Framework (QOF) over time.(46,47)  The study will be unable to account for potential confounders not available in the CPRD (such as migration status, education, employment status, diet, exercise), or inconsistently recorded (such as country of birth, religion, first language). These factors may mediate ethnic differences in consultation rates, timely identification of diabetes risk factors, and subsequent vascular disease diagnoses. |
| 1. **References**   1. Newman CB, Szarek M, Colhoun HM, Betteridge DJ, Durrington PN, Hitman GA, *et al.* The safety and tolerability of atorvastatin 10 mg in the Collaborative Atorvastatin Diabetes Study (CARDS). *Diab Vasc Dis Res* 2008;5:177–83.  2. Stratton IM, Adler AI, Neil HA, Matthews DR, Manley SE, Cull CA, *et al.* Association of glycaemia with macrovascular and microvascular complications of type 2 diabetes (UKPDS 35): Prospective observational study. *BMJ* 2000;321:405–12.  3. Holman R, TUrner R, Stratton I, Cull C, Frighi V, Manley S, *et al.* Efficacy of atenolol and captopril in reducing risk of macrovascular and microvascular complications in type 2 diabetes: UKPDS 39. UK Prospective Diabetes Study Group. *BMJ* 1998;317:713–20.  4. Stratton IM, Cull CA, Adler AI, Matthews DR, Neil HA, Holman RR, *et al.* Additive effects of glycaemia and blood pressure exposure on risk of complications in type 2 diabetes: a prospective observational study (UKPDS 75). *Diabetologia* 2006;49:1761–9.  5. Shepherd J, Barter P, Carmena R, Deedwania P, Fruchart J-C, Haffner S, *et al.* Effect of lowering LDL cholesterol substantially below currently recommended levels in patients with coronary heart disease and diabetes: the Treating to New Targets (TNT) study. *Diabetes Care* 2006;29:1220–6.  6. Juutilainen A, Lehto S, Ronnemaa T, Pyorala K, Laakso M. Type 2 diabetes as a ‘coronary heart disease equivalent’: an 18-year prospective population-based study in Finnish subjects. *Diabetes Care* 2005;28:2901–7.  7. M. H, Hanefeld M. Outcome studies in type 2 diabetes. *Curr Med Res Opin* 2005;21 Suppl 1:S41–8.  8. UK Prospective Diabetes Study Group. Intensive blood-glucose control with sulphonylureas or insulin compared with conventional treatment and risk of complications in patients with type 2 diabetes ( UKPDS 33 ). 1998;352:837–53.  9. Oldroyd J, Banerjee M, Heald A, Cruickshank K, J. O, M. B, *et al.* Diabetes and ethnic minorities. *Postgrad Med J* 2005;81:486–90.  10. Tillin T, Forouhi NG, McKeigue PM, Chaturvedi N. Southall And Brent REvisited: Cohort profile of SABRE, a UK population-based comparison of cardiovascular disease and diabetes in people of European, Indian Asian and African Caribbean origins. *Int J Epidemiol* 2012;41:33–42.  11. Davis TME. Ethnic diversity in Type 2 diabetes. *Diabet Med* 2008;25:52–6.  12. Eastwood S V., Tillin T, Chaturvedi N, Hughes AD. Ethnic Differences in Associations Between Blood Pressure and Stroke in South Asian and European Men. *Hypertension* 2015;66:481–8.  13. Tillin T, Hughes AD, Godsland IF, Whincup P, Forouhi NG, Welsh P, *et al.* Insulin resistance and truncal obesity as important determinants of the greater incidence of diabetes in Indian Asians and African Caribbeans compared with Europeans: the Southall And Brent REvisited (SABRE) cohort. *Diabetes Care* 2013;36:383–93.  14. Hartland AJ, DeVille-Almond J, Orton M, Hartland V. Age of diagnosis of type 2 diabetes is more than 9 years younger in Asian-British compared with the rest of the UK population. Diabetes. 2013;62:A365.  15. Bakker LE, Sleddering MA, Schoones JW, Meinders AE, Jazet IM. Pathogenesis of type 2 diabetes in South Asians. *Eur J Endocrinol* 2013;169:R99–114.  16. Bellary S, O’Hare JP, Raymond NT, Mughal S, Hanif WM, Jones A, *et al.* Premature cardiovascular events and mortality in south Asians with type 2 diabetes in the United Kingdom Asian Diabetes Study effect of ethnicity on risk. *Curr Med Res Opin* 2010;26:1873–9.  17. Adler AI, Neil HAW, Stratton IM, Holman R., Turner R. Ethnicity and cardiovascular disease: The incidence of myocardial infarction in white, South Asian, and Afro-Caribbean patients with type 2 diabetes (U.K. prospective diabetes study 32). Diabetes Care. 1998;21:1271–7.  18. Tillin T, Hughes AD, Mayet J, Whincup P, Sattar N, Forouhi NG, *et al.* The relationship between metabolic risk factors and incident cardiovascular disease in Europeans, South Asians, and African Caribbeans: SABRE (Southall and Brent Revisited) - A prospective population-based study. *J Am Coll Cardiol* 2013;61:1777–86.  19. Dreyer G, Hull S, Mathur R, Chesser a., Yaqoob MM. Progression of chronic kidney disease in a multi-ethnic community cohort of patients with diabetes mellitus. *Diabet Med* 2013;30:956–63.  20. Davis TME, Coleman RL, Holman RR. Ethnicity and long-term vascular outcomes in Type 2 diabetes: A prospective observational study (UKPDS 83). *Diabet Med* 2014;31:200–7.  21. Depressionartment of Health. *Equity and excellence: liberating the NHS (White Paper)*. 2010. doi:10.1136/adc.2010.205294  22. Burt J, Lloyd C, Campbell J, Roland M, Abel G. Variations in GP-patient communication by ethnicity, age, and gender: evidence from a national primary care patient survey. *Br J Gen Pract* Published Online First: 5 November 2015. doi:10.3399/bjgp15X687637  23. Lyratzopoulos G, Elliott M, Barbiere JM, Henderson A, Staetsky L, Paddison C, *et al.* Understanding ethnic and other socio-demographic differences in patient experience of primary care: evidence from the English General Practice Patient Survey. *BMJ Qual Saf* 2012;21:21–9.  24. Hull S, Dreyer G, Badrick E, Chesser A, Yaqoob M. The relationship of ethnicity to the prevalence and management of hypertension and associated chronic kidney disease. *BMC Nephrol* 2011;12:41.  25. Fischbacher CM, Bhopal R, Steiner M, Morris a. D, Chalmers J, C.M. F, *et al.* Is there equity of service delivery and intermediate outcomes in South Asians with type 2 diabetes? Analysis of DARTS database and summary of UK publications. *J Public Health (Oxf)* 2009;31:239–49.  26. Schofield P, Saka O, Ashworth M. Ethnic differences in blood pressure monitoring and control in South East London. *Br J Gen Pract* 2011;61:e190–6.  27. Alshamsan R, Lee JT, Majeed A, Netuveli G, Millett C. Effect of a UK pay-for-performance program on ethnic disparities in diabetes outcomes: Interrupted time series analysis. *Ann Fam Med* 2012;10:228–34.  28. James GD, Baker P, Badrick E, Mathur R, Hull S, Robson J. Type 2 diabetes: a cohort study of treatment, ethnic and social group influences on glycated haemoglobin. *BMJ Open* 2012;2:e001477–e001477.  29. Forde I, Chandola T, Raine R, Marmot MG, Kivimaki M. Socioeconomic and ethnic differences in use of lipid-lowering drugs after deregulation of simvastatin in the UK: The Whitehall II prospective cohort study. *Atherosclerosis* 2011;215:223–8.  30. Verma A, Birger R, Bhatt H, Murray J, Millett C, Saxena S, *et al.* Ethnic disparities in diabetes management: a 10-year population-based repeated cross-sectional study in UK primary care. *J Public Health (Bangkok)* 2010;32:250–8.  31. National Institute for Health and Care Excellence. Clinical management of primary hypertension in adults. 2011.  32. National Institute for Health and Care Excellence. Preventing type 2 diabetes : risk identification and interventions for individuals at high risk. 2012.  33. National Institute for Health and Care Excellence. Chronic obstructive pulmonary disease. 2010.  34. National Institute for Health and Care Excellence. Prevention of cardiovascular disease. 2010.  35. Mathur R, Bhaskaran K, Chaturvedi N, Leon DA, vanStaa T, Grundy E, *et al.* Completeness and usability of ethnicity data in UK-based primary care and hospital databases. *J Public Health (Oxf)* 2014;36. doi:10.1093/pubmed/fdt116  36. Hippisley-Cox J, Vinogradova Y. Trends in consultation rates in general practice 1995/1996 to 2008/2009: analysis of the QResearch database. *London QResearch Inf Cent …* 2009;:1–24.  37. The National Institute for Health and Care Excellence. Type 2 diabetes in adults: management NICE guidelines [NG28]. http://www.nice.org.uk/guidance/ng28/chapter/1-Recommendations#blood-pressure-management-2 (accessed 14 Dec 2015).  38. The National Institute for Health and Care Excellence. Hypertension in adults: diagnosis and management [CG127]. NICE 2013. http://www.nice.org.uk/guidance/CG127 (accessed 14 Dec 2015).  39. The National Institute for Health and Care Excellence. Algorithm for blood glucose lowering therapy in adults with type 2 diabetes: Type 2 diabaetes in adults: management [NG28]. 2015.  40. Walraven I, Mast M, Hoekstra T, Jansen A, van der Heijden A, Rauh S, *et al.* Classes and characteristics of distinct HbA1c trajectories in a type 2 diabetes cohort. *Prep* 2015;:267–75.  41. Walraven I, Mast MR, Hoekstra T, Jansen APD, Rauh SP, Rutters FR, *et al.* Real-world evidence of suboptimal blood pressure control in patients with type 2 diabetes. *J Hypertens* 2015;:1.  42. Robins JM, Robins JM, Hernán M a, Hernán M a, Brumback B, Brumback B. Marginal structural models and causal inference in epidemiology. *Epidemiology* 2000;11:550–60.  43. Hernán MA, Brumback B, Robins JM. Marginal structural models to estimate the causal effect of zidovudine on the survival of HIV-positive men. *Epidemiology* 2000;11:561–70.  44. The NHS Information Centre. Prescribing compliance: a review of the proportion of prescriptions dispensed. 2011;:1–37.  45. Hull SA, Mathur R, Badrick E, Robson J, Boomla K. Recording ethnicity in primary care: assessing the methods and impact. *Br J Gen Pract*;61:e290-4.  46. Khan NF, Harrison SE, Rose PW. Validity of diagnostic coding within the General Practice Research Database: a systematic review. *Br J Gen Pract* 2010;60:e128-36.  47. Herrett E, Thomas SL, Schoonen WM, Smeeth L, Hall AJ. Validation and validity of diagnoses in the General Practice Research Database: a systematic review. *Br J Clin Pharmacol* 2010;69:4–14. |
| **List of Appendices** *(Submit all appendices as separate documents to this application)* |
