# Supplementary material for: Ethnic disparities in initiation and intensification of diabetes treatment in adults with type 2 diabetes in the UK, 1990–2017: A cohort study
Source: PLoS Med. 2020 May 15;17(5):e1003106. doi: 10.1371/journal.pmed.1003106 (PMC7228040; doi:10.1371/journal.pmed.1003106)
Supplement: S1 Table — (DOCX) [file pmed.1003106.s007.docx]

Table S2. Code lists for all study variables

| CPRD Medcode | Readcode | Readterm |  |  |
| --- | --- | --- | --- | --- |
| Ethnicity |  |  | Ethnicity in 16 categories | Ethnicity in 5 categories |
| 10196 | 9S...00 | Ethnic groups (1991 census) | Not Stated | Not Stated |
| 22467 | 9S1..00 | White | British | White |
| 12446 | 9S10.00 | White British | British | White |
| 24837 | 9S11.00 | White Irish | Irish | White |
| 12444 | 9S12.00 | Other white ethnic group | Other White | White |
| 26467 | 9S13.00 | White Scottish | Other White | White |
| 26310 | 9S14.00 | Other white British ethnic group | British | White |
| 12632 | 9S2..00 | Black Caribbean | Caribbean | Black |
| 12778 | 9S3..00 | Black African | African | Black |
| 24339 | 9S4..00 | Black, other, non-mixed origin | Other Black | Black |
| 12452 | 9S41.00 | Black British | Other Black | Black |
| 57435 | 9S42.00 | Black Caribbean/W.I./Guyana | Other Black | Black |
| 47950 | 9S42.11 | Black Caribbean | Other Black | Black |
| 47997 | 9S42.12 | Black West Indian | Other Black | Black |
| 32100 | 9S42.13 | Black Guyana | Other Black | Black |
| 41329 | 9S43.00 | Black N African/Arab/Iranian | Other Black | Black |
| 46812 | 9S43.11 | Black North African | Other Black | Black |
| 57752 | 9S43.12 | Black Arab | Other Black | Black |
| 50286 | 9S43.13 | Black Iranian | Other Black | Black |
| 35412 | 9S44.00 | Black - other African country | African | Black |
| 47965 | 9S45.00 | Black E Afric Asia/Indo-Caribb | Other Black | Black |
| 57753 | 9S45.11 | Black East African Asian | Other Black | Black |
| 57763 | 9S45.12 | Black Indo-Caribbean | Other Black | Black |
| 48005 | 9S46.00 | Black Indian sub-continent | Other Black | Black |
| 35350 | 9S47.00 | Black - other Asian | Other Black | Black |
| 26312 | 9S48.00 | Black Black - other | Other Black | Black |
| 25676 | 9S5..00 | Black - other, mixed | Other Black | Black |
| 25623 | 9S51.00 | Other Black - Black/White orig | Other Mixed | Mixed |
| 32165 | 9S52.00 | Other Black - Black/Asian orig | Other Mixed | Mixed |
| 12482 | 9S6..00 | Indian | Indian | South Asian |
| 24690 | 9S7..00 | Pakistani | Pakistani | South Asian |
| 24740 | 9S8..00 | Bangladeshi | Bangladeshi | South Asian |
| 24272 | 9S9..00 | Chinese | Chinese | Other |
| 30280 | 9SA..00 | Other ethnic non-mixed (NMO) | Other ethnic group | Other |
| 32110 | 9SA1.00 | Brit. ethnic minor. spec.(NMO) | Other ethnic group | Other |
| 57764 | 9SA2.00 | Brit. ethnic minor. unsp (NMO) | Other ethnic group | Other |
| 54593 | 9SA3.00 | Caribbean I./W.I./Guyana (NMO) | Other Black | Black |
| 57094 | 9SA3.11 | Caribbean Island (NMO) | Other Black | Black |
| 57075 | 9SA3.12 | West Indian (NMO) | Other Black | Black |
| 93144 | 9SA3.13 | Guyana (NMO) | Other Black | Black |
| 24962 | 9SA4.00 | N African Arab/Iranian (NMO) | Other ethnic group | Other |
| 47285 | 9SA4.11 | North African Arab (NMO) | Other ethnic group | Other |
| 25082 | 9SA4.12 | Iranian (NMO) | Other ethnic group | Other |
| 47969 | 9SA5.00 | Other African countries (NMO) | African | Black |
| 38097 | 9SA6.00 | E Afric Asian/Indo-Carib (NMO) | Other Asian | South Asian |
| 46818 | 9SA6.11 | East African Asian (NMO) | Other Asian | South Asian |
| 99316 | 9SA6.12 | Indo-Caribbean (NMO) | Other Asian | South Asian |
| 39696 | 9SA7.00 | Indian sub-continent (NMO) | Other Asian | South Asian |
| 26379 | 9SA8.00 | Other Asian (NMO) | Other Asian | South Asian |
| 24270 | 9SA9.00 | Irish (NMO) | Irish | White |
| 45947 | 9SAA.00 | Greek/Greek Cypriot (NMO) | Other ethnic group | Other |
| 45955 | 9SAA.11 | Greek (NMO) | Other ethnic group | Other |
| 47949 | 9SAA.12 | Greek Cypriot (NMO) | Other ethnic group | Other |
| 32066 | 9SAB.00 | Turkish/Turkish Cypriot (NMO) | Other ethnic group | Other |
| 32126 | 9SAB.11 | Turkish (NMO) | Other ethnic group | Other |
| 32069 | 9SAB.12 | Turkish Cypriot (NMO) | Other ethnic group | Other |
| 12633 | 9SAC.00 | Other European (NMO) | Other ethnic group | Other |
| 41214 | 9SAD.00 | Other ethnic NEC (NMO) | Other ethnic group | Other |
| 12696 | 9SB..00 | Other ethnic, mixed origin | Other Mixed | Mixed |
| 47401 | 9SB1.00 | Other ethnic, Black/White orig | Other Mixed | Mixed |
| 32401 | 9SB2.00 | Other ethnic, Asian/White orig | White and Asian | Mixed |
| 35459 | 9SB3.00 | Other ethnic, mixed white orig | Other Mixed | Mixed |
| 32420 | 9SB4.00 | Other ethnic, other mixed orig | Other Mixed | Mixed |
| 32425 | 9SB5.00 | Black Caribbean and White | White and Black Caribbean | Mixed |
| 32443 | 9SB6.00 | Black African and White | White and Black African | Mixed |
| 25411 | 9SC..00 | Vietnamese | Other ethnic group | Other |
| 12429 | 9SD..00 | Ethnic group not given - patient refused | Not Stated | Not Stated |
| 24340 | 9SE..00 | Ethnic group not recorded | Not Stated | Not Stated |
| 32136 | 9SG..00 | Other black ethnic group | Other Black | Black |
| 12668 | 9SH..00 | Other Asian ethnic group | Other Asian | South Asian |
| 47601 | 9SI..00 | Irish traveller | Irish | White |
| 12757 | 9SJ..00 | Other ethnic group | Other ethnic group | Other |
| 45199 | 9SZ..00 | Ethnic groups (census) NOS | Not Stated | Not Stated |
| 12435 | 9i...00 | Ethnic category - 2001 census | Not Stated | Not Stated |
| 12351 | 9i0..00 | British or mixed British - ethnic category 2001 census | British | White |
| 98111 | 9i00.00 | White British - ethnic category 2001 census | British | White |
| 12532 | 9i1..00 | Irish - ethnic category 2001 census | Irish | White |
| 98213 | 9i10.00 | White Irish - ethnic category 2001 census | Irish | White |
| 12421 | 9i2..00 | Other White background - ethnic category 2001 census | Other White | White |
| 12352 | 9i20.00 | English - ethnic category 2001 census | Other White | White |
| 12436 | 9i21.00 | Scottish - ethnic category 2001 census | Other White | White |
| 12681 | 9i22.00 | Welsh - ethnic category 2001 census | Other White | White |
| 28887 | 9i23.00 | Cornish - ethnic category 2001 census | Other White | White |
| 42294 | 9i24.00 | Northern Irish - ethnic category 2001 census | Other White | White |
| 40102 | 9i25.00 | Ulster Scots - ethnic category 2001 census | Other White | White |
| 32778 | 9i26.00 | Cypriot (part not stated) - ethnic category 2001 census | Other White | White |
| 12355 | 9i27.00 | Greek - ethnic category 2001 census | Other White | White |
| 12769 | 9i28.00 | Greek Cypriot - ethnic category 2001 census | Other White | White |
| 12746 | 9i29.00 | Turkish - ethnic category 2001 census | Other White | White |
| 32413 | 9i2A.00 | Turkish Cypriot - ethnic category 2001 census | Other White | White |
| 12412 | 9i2B.00 | Italian - ethnic category 2001 census | Other White | White |
| 55223 | 9i2C.00 | Irish Traveller - ethnic category 2001 census | Other White | White |
| 55113 | 9i2D.00 | Traveller - ethnic category 2001 census | Other White | White |
| 42290 | 9i2E.00 | Gypsy/Romany - ethnic category 2001 census | Other White | White |
| 12467 | 9i2F.00 | Polish - ethnic category 2001 census | Other White | White |
| 12433 | 9i2G.00 | Baltic Estonian/Latvian/Lithuanian - ethn categ 2001 census | Other White | White |
| 28973 | 9i2H.00 | Commonwealth (Russian) Indepression States - ethn categ 2001 census | Other White | White |
| 26341 | 9i2J.00 | Kosovan - ethnic category 2001 census | Other White | White |
| 25422 | 9i2K.00 | Albanian - ethnic category 2001 census | Other White | White |
| 46956 | 9i2L.00 | Bosnian - ethnic category 2001 census | Other White | White |
| 28866 | 9i2M.00 | Croatian - ethnic category 2001 census | Other White | White |
| 47074 | 9i2N.00 | Serbian - ethnic category 2001 census | Other White | White |
| 28936 | 9i2P.00 | Other republics former Yugoslavia - ethnic categ 2001 census | Other White | White |
| 26391 | 9i2Q.00 | Mixed Irish and other White - ethnic category 2001 census | Other White | White |
| 12402 | 9i2R.00 | Oth White European/European unsp/Mixed European 2001 census | Other White | White |
| 28900 | 9i2S.00 | Other mixed White - ethnic category 2001 census | Other White | White |
| 12591 | 9i2T.00 | Other White or White unspecified ethnic category 2001 census | Other White | White |
| 12742 | 9i3..00 | White and Black Caribbean - ethnic category 2001 census | White and Black Caribbean | Mixed |
| 12437 | 9i4..00 | White and Black African - ethnic category 2001 census | White and Black African | Mixed |
| 12638 | 9i5..00 | White and Asian - ethnic category 2001 census | White and Asian | Mixed |
| 12873 | 9i6..00 | Other Mixed background - ethnic category 2001 census | Other Mixed | Mixed |
| 12795 | 9i60.00 | Black and Asian - ethnic category 2001 census | Other Mixed | Mixed |
| 49940 | 9i61.00 | Black and Chinese - ethnic category 2001 census | Other Mixed | Mixed |
| 40110 | 9i62.00 | Black and White - ethnic category 2001 census | Other Mixed | Mixed |
| 12706 | 9i63.00 | Chinese and White - ethnic category 2001 census | Other Mixed | Mixed |
| 47005 | 9i64.00 | Asian and Chinese - ethnic category 2001 census | Other Mixed | Mixed |
| 32408 | 9i65.00 | Other Mixed or Mixed unspecified ethnic category 2001 census | Other Mixed | Mixed |
| 12414 | 9i7..00 | Indian or British Indian - ethnic category 2001 census | Indian | South Asian |
| 12460 | 9i8..00 | Pakistani or British Pakistani - ethnic category 2001 census | Pakistani | South Asian |
| 28888 | 9i9..00 | Bangladeshi or British Bangladeshi - ethn categ 2001 census | Bangladeshi | South Asian |
| 12513 | 9iA..00 | Other Asian background - ethnic category 2001 census | Other Asian | South Asian |
| 26392 | 9iA1.00 | Punjabi - ethnic category 2001 census | Other Asian | South Asian |
| 64133 | 9iA2.00 | Kashmiri - ethnic category 2001 census | Other Asian | South Asian |
| 47077 | 9iA3.00 | East African Asian - ethnic category 2001 census | Other Asian | South Asian |
| 12608 | 9iA4.00 | Sri Lankan - ethnic category 2001 census | Other Asian | South Asian |
| 12760 | 9iA5.00 | Tamil - ethnic category 2001 census | Other Asian | South Asian |
| 12887 | 9iA6.00 | Sinhalese - ethnic category 2001 census | Other Asian | South Asian |
| 32399 | 9iA7.00 | Caribbean Asian - ethnic category 2001 census | Other Asian | South Asian |
| 12653 | 9iA8.00 | British Asian - ethnic category 2001 census | Other Asian | South Asian |
| 46056 | 9iA9.00 | Mixed Asian - ethnic category 2001 census | Other Asian | South Asian |
| 28935 | 9iAA.00 | Other Asian or Asian unspecified ethnic category 2001 census | Other Asian | South Asian |
| 12432 | 9iB..00 | Caribbean - ethnic category 2001 census | Caribbean | Black |
| 12350 | 9iC..00 | African - ethnic category 2001 census | African | Black |
| 32389 | 9iD..00 | Other Black background - ethnic category 2001 census | Other Black | Black |
| 12443 | 9iD0.00 | Somali - ethnic category 2001 census | Other Black | Black |
| 32886 | 9iD1.00 | Nigerian - ethnic category 2001 census | Other Black | Black |
| 40097 | 9iD2.00 | Black British - ethnic category 2001 census | Other Black | Black |
| 40096 | 9iD3.00 | Mixed Black - ethnic category 2001 census | Other Black | Black |
| 46047 | 9iD4.00 | Other Black or Black unspecified ethnic category 2001 census | Other Black | Black |
| 12468 | 9iE..00 | Chinese - ethnic category 2001 census | Chinese | Other |
| 12434 | 9iF..00 | Other - ethnic category 2001 census | Other ethnic group | Other |
| 12719 | 9iF0.00 | Vietnamese - ethnic category 2001 census | Other ethnic group | Other |
| 12473 | 9iF1.00 | Japanese - ethnic category 2001 census | Other ethnic group | Other |
| 12420 | 9iF2.00 | Filipino - ethnic category 2001 census | Other ethnic group | Other |
| 12730 | 9iF3.00 | Malaysian - ethnic category 2001 census | Other ethnic group | Other |
| 63872 | 9iF4.00 | Buddhist - ethnic category 2001 census | Other ethnic group | Other |
| 56127 | 9iF5.00 | Hindu - ethnic category 2001 census | Other ethnic group | Other |
| 46063 | 9iF6.00 | Jewish - ethnic category 2001 census | Other ethnic group | Other |
| 47091 | 9iF7.00 | Muslim - ethnic category 2001 census | Other ethnic group | Other |
| 49658 | 9iF8.00 | Sikh - ethnic category 2001 census | Other ethnic group | Other |
| 46059 | 9iF9.00 | Arab - ethnic category 2001 census | Other ethnic group | Other |
| 47028 | 9iFA.00 | North African - ethnic category 2001 census | Other ethnic group | Other |
| 28909 | 9iFB.00 | Mid East (excl Israeli, Iranian & Arab) - eth cat 2001 cens | Other ethnic group | Other |
| 46964 | 9iFC.00 | Israeli - ethnic category 2001 census | Other ethnic group | Other |
| 25937 | 9iFD.00 | Iranian - ethnic category 2001 census | Other ethnic group | Other |
| 45964 | 9iFE.00 | Kurdish - ethnic category 2001 census | Other ethnic group | Other |
| 25451 | 9iFF.00 | Moroccan - ethnic category 2001 census | Other ethnic group | Other |
| 26246 | 9iFG.00 | Latin American - ethnic category 2001 census | Other ethnic group | Other |
| 12756 | 9iFH.00 | South and Central American - ethnic category 2001 census | Other ethnic group | Other |
| 32382 | 9iFJ.00 | Mauritian/Seychellois/Maldivian/St Helena eth cat 2001census | Other ethnic group | Other |
| 26455 | 9iFK.00 | Any other group - ethnic category 2001 census | Other ethnic group | Other |
| 12459 | 9iG..00 | Ethnic category not stated - 2001 census | Not Stated | Not Stated |
| Type 2 diabetes |  |  |  |  |
|  | C10FL | Definite T2 codes |  |  |
|  | C10F4 | Definite T2 codes |  |  |
|  | C10F6 | Definite T2 codes |  |  |
|  | C10FQ | Definite T2 codes |  |  |
|  | C10FP | Definite T2 codes |  |  |
|  | C10F9 | Definite T2 codes |  |  |
|  | C10FG | Definite T2 codes |  |  |
|  | C10FH | Definite T2 codes |  |  |
|  | C10FK | Definite T2 codes |  |  |
|  | C10FJ | Definite T2 codes |  |  |
|  | C10FD | Definite T2 codes |  |  |
|  | C10FR | Definite T2 codes |  |  |
|  | C10FA | Definite T2 codes |  |  |
|  | C10FN | Definite T2 codes |  |  |
|  | C10F3 | Definite T2 codes |  |  |
|  | C10F5 | Definite T2 codes |  |  |
|  | C10FC | Definite T2 codes |  |  |
|  | C10F. | Definite T2 codes |  |  |
|  | C10F2 | Definite T2 codes |  |  |
|  | C10F1 | Definite T2 codes |  |  |
|  | C10FM | Definite T2 codes |  |  |
|  | C10F0 | Definite T2 codes |  |  |
|  | C10FB | Definite T2 codes |  |  |
|  | C10FE | Definite T2 codes |  |  |
|  | C10FF | Definite T2 codes |  |  |
|  | C10F7 | Definite T2 codes |  |  |
|  | C1071 | Possible T2 codes |  |  |
|  | C1051 | Possible T2 codes |  |  |
|  | C1041 | Possible T2 codes |  |  |
|  | C1072 | Possible T2 codes |  |  |
|  | C112z | Possible T2 codes |  |  |
|  | C112. | Possible T2 codes |  |  |
|  | L180X | Possible T2 codes |  |  |
|  | C1021 | Possible T2 codes |  |  |
|  | C1031 | Possible T2 codes |  |  |
|  | C1061 | Possible T2 codes |  |  |
|  | C1001 | Possible T2 codes |  |  |
|  | L1806 | Probable T2 codes |  |  |
|  | C1099 | Probable T2 codes |  |  |
|  | C109J | Probable T2 codes |  |  |
|  | C1090 | Probable T2 codes |  |  |
|  | C109E | Probable T2 codes |  |  |
|  | C109G | Probable T2 codes |  |  |
|  | C109. | Probable T2 codes |  |  |
|  | C1097 | Probable T2 codes |  |  |
|  | C109D | Probable T2 codes |  |  |
|  | C1095 | Probable T2 codes |  |  |
|  | C1091 | Probable T2 codes |  |  |
|  | C109H | Probable T2 codes |  |  |
|  | C1096 | Probable T2 codes |  |  |
|  | C109B | Probable T2 codes |  |  |
|  | C1074 | Probable T2 codes |  |  |
|  | C1094 | Probable T2 codes |  |  |
|  | C109C | Probable T2 codes |  |  |
|  | C109K | Probable T2 codes |  |  |
|  | C1093 | Probable T2 codes |  |  |
|  | C10y1 | Probable T2 codes |  |  |
|  | C1092 | Probable T2 codes |  |  |
|  | C10z1 | Probable T2 codes |  |  |
|  | C109F | Probable T2 codes |  |  |
|  | C109A | Probable T2 codes |  |  |
| Smoking status |  |  |  |  |
| medcode | readcode | readterm |  |  |
| 33 | 1371.00 | Never smoked tobacco |  |  |
| 54 | 137..00 | Tobacco consumption |  |  |
| 60 | 137L.00 | Current non-smoker |  |  |
| 90 | 137S.00 | Ex smoker |  |  |
| 93 | 137P.00 | Cigarette smoker |  |  |
| 776 | 137K.00 | Stopped smoking |  |  |
| 1822 | 1376.00 | Very heavy smoker - 40+cigs/d |  |  |
| 1823 | 137P.11 | Smoker |  |  |
| 1878 | 1374.00 | Moderate smoker - 10-19 cigs/d |  |  |
| 3568 | 1375.00 | Heavy smoker - 20-39 cigs/day |  |  |
| 10558 | 137R.00 | Current smoker |  |  |
| 11788 | 1371.11 | Non-smoker |  |  |
| 12240 | 137G.00 | Trying to give up smoking |  |  |
| 12878 | 137T.00 | Date ceased smoking |  |  |
| 12941 | 1372.11 | Occasional smoker |  |  |
| 12942 | 137..11 | Smoker - amount smoked |  |  |
| 12943 | 137J.00 | Cigar smoker |  |  |
| 12944 | 1373.00 | Light smoker - 1-9 cigs/day |  |  |
| 12945 | 137M.00 | Rolls own cigarettes |  |  |
| 12946 | 137F.00 | Ex-smoker - amount unknown |  |  |
| 12947 | 137H.00 | Pipe smoker |  |  |
| 12951 | 137Q.11 | Smoking restarted |  |  |
| 12952 | 137Q.00 | Smoking started |  |  |
| 12955 | 1379.00 | Ex-moderate smoker (10-19/day) |  |  |
| 12956 | 137A.00 | Ex-heavy smoker (20-39/day) |  |  |
| 12957 | 1378.00 | Ex-light smoker (1-9/day) |  |  |
| 12958 | 1372.00 | Trivial smoker - < 1 cig/day |  |  |
| 12959 | 137B.00 | Ex-very heavy smoker (40+/day) |  |  |
| 12960 | 137Z.00 | Tobacco consumption NOS |  |  |
| 12961 | 1377.00 | Ex-trivial smoker (<1/day) |  |  |
| 12962 | 137E.00 | Tobacco consumption unknown |  |  |
| 12963 | 137Y.00 | Cigar consumption |  |  |
| 12964 | 137C.00 | Keeps trying to stop smoking |  |  |
| 12965 | 137X.00 | Cigarette consumption |  |  |
| 12966 | 137V.00 | Smoking reduced |  |  |
| 12967 | 137a.00 | Pipe tobacco consumption |  |  |
| 13351 | 137I.00 | Passive smoker |  |  |
| 19488 | 137O.00 | Ex cigar smoker |  |  |
| 23017 | 137U.00 | Not a passive smoker |  |  |
| 26470 | 137N.00 | Ex pipe smoker |  |  |
| 30423 | 137c.00 | Thinking about stopping smoking |  |  |
| 30762 | 137d.00 | Not interested in stopping smoking |  |  |
| 31114 | 137b.00 | Ready to stop smoking |  |  |
| 32973 | 137W.00 | Chews tobacco |  |  |
| 41979 | 137e.00 | Smoking restarted |  |  |
| 46300 | 137g.00 | Cigarette pack-years |  |  |
| 46321 | 137f.00 | Reason for restarting smoking |  |  |
| 46654 | 137D.00 | Admitted tobacco cons untrue ? |  |  |
| 62686 | 137h.00 | Minutes from waking to first tobacco consumption |  |  |
| 97029 | 137k.00 | Refusal to give smoking status |  |  |
| 97210 | 137j.00 | Ex-cigarette smoker |  |  |
| 99838 | 137K000 | Recently stopped smoking |  |  |
| 100495 | 137l.00 | Ex roll-up cigarette smoker |  |  |
| 101069 | 137I000 | Exposed to tobacco smoke at home |  |  |
| 101338 | 137m.00 | Failed attempt to stop smoking |  |  |
| 105501 | 137o.00 | Waterpipe tobacco consumption |  |  |
| 105711 | 137n.00 | Total time smoked |  |  |
| 106891 | 137i.00 | Ex-tobacco chewer |  |  |
| Coronary Heart Disease (QOF Definition) |  |  |  |  |
| 240 | G3...00 | Ischaemic heart disease |  |  |
| 241 | G30..00 | Acute myocardial infarction |  |  |
| 1204 | G30..14 | Heart attack |  |  |
| 1344 | G340.12 | Coronary artery disease |  |  |
| 1414 | G33z300 | Angina on effort |  |  |
| 1430 | G33..00 | Angina pectoris |  |  |
| 1431 | G311.13 | Unstable angina |  |  |
| 1655 | G340.11 | Triple vessel disease of the heart |  |  |
| 1676 | G3z..00 | Ischaemic heart disease NOS |  |  |
| 1677 | G30..15 | MI - acute myocardial infarction |  |  |
| 1678 | G308.00 | Inferior myocardial infarction NOS |  |  |
| 1792 | G3...13 | IHD - Ischaemic heart disease |  |  |
| 2491 | G30..12 | Coronary thrombosis |  |  |
| 3704 | G307.00 | Acute subendocardial infarction |  |  |
| 3999 | G340000 | Single coronary vessel disease |  |  |
| 4017 | G32..00 | Old myocardial infarction |  |  |
| 4656 | G311.11 | Crescendo angina |  |  |
| 5254 | G340100 | Double coronary vessel disease |  |  |
| 5387 | G301.00 | Other specified anterior myocardial infarction |  |  |
| 5413 | G340.00 | Coronary atherosclerosis |  |  |
| 7320 | G343.00 | Ischaemic cardiomyopathy |  |  |
| 7347 | G311100 | Unstable angina |  |  |
| 7696 | G33z200 | Syncope anginosa |  |  |
| 8935 | G302.00 | Acute inferolateral infarction |  |  |
| 9276 | G31y000 | Acute coronary insufficiency |  |  |
| 9413 | G31y.00 | Other acute and subacute ischaemic heart disease |  |  |
| 9507 | G307000 | Acute non-Q wave infarction |  |  |
| 9555 | G33z500 | Post infarct angina |  |  |
| 10562 | G307100 | Acute non-ST segment elevation myocardial infarction |  |  |
| 11983 | G311500 | Acute coronary syndrome |  |  |
| 12139 | G300.00 | Acute anterolateral infarction |  |  |
| 12229 | G30X000 | Acute ST segment elevation myocardial infarction |  |  |
| 12804 | G33z700 | Stable angina |  |  |
| 13566 | G30..11 | Attack - heart |  |  |
| 13571 | G30..16 | Thrombosis - coronary |  |  |
| 14658 | G30z.00 | Acute myocardial infarction NOS |  |  |
| 14897 | G301z00 | Anterior myocardial infarction NOS |  |  |
| 14898 | G305.00 | Lateral myocardial infarction NOS |  |  |
| 15754 | G34z.00 | Other chronic ischaemic heart disease NOS |  |  |
| 16408 | G32..11 | Healed myocardial infarction |  |  |
| 17307 | G311200 | Angina at rest |  |  |
| 17464 | G32..12 | Personal history of myocardial infarction |  |  |
| 17689 | G30..17 | Silent myocardial infarction |  |  |
| 17872 | G301100 | Acute anteroseptal infarction |  |  |
| 18118 | G311400 | Worsening angina |  |  |
| 18125 | G330000 | Nocturnal angina |  |  |
| 18842 | G35..00 | Subsequent myocardial infarction |  |  |
| 18889 | G34z000 | Asymptomatic coronary heart disease |  |  |
| 19655 | G311.14 | Angina at rest |  |  |
| 20095 | G330.00 | Angina decubitus |  |  |
| 20416 | G3...12 | Atherosclerotic heart disease |  |  |
| 21844 | G31y300 | Transient myocardial ischaemia |  |  |
| 22383 | G3y..00 | Other specified ischaemic heart disease |  |  |
| 23078 | G34y100 | Chronic myocardial ischaemia |  |  |
| 23892 | G304.00 | Posterior myocardial infarction NOS |  |  |
| 24540 | G34y000 | Chronic coronary insufficiency |  |  |
| 24783 | G3...11 | Arteriosclerotic heart disease |  |  |
| 25842 | G33z.00 | Angina pectoris NOS |  |  |
| 26863 | G33z600 | New onset angina |  |  |
| 27951 | G31..00 | Other acute and subacute ischaemic heart disease |  |  |
| 27977 | G31yz00 | Other acute and subacute ischaemic heart disease NOS |  |  |
| 28138 | G34..00 | Other chronic ischaemic heart disease |  |  |
| 28554 | G33zz00 | Angina pectoris NOS |  |  |
| 28736 | G30y000 | Acute atrial infarction |  |  |
| 29421 | G344.00 | Silent myocardial ischaemia |  |  |
| 29643 | G303.00 | Acute inferoposterior infarction |  |  |
| 29758 | G30X.00 | Acute transmural myocardial infarction of unspecif site |  |  |
| 29902 | G330z00 | Angina decubitus NOS |  |  |
| 30330 | G309.00 | Acute Q-wave infarct |  |  |
| 30421 | G30..13 | Cardiac rupture following myocardial infarction (MI) |  |  |
| 32272 | G38..00 | Postoperative myocardial infarction |  |  |
| 32450 | G33z400 | Ischaemic chest pain |  |  |
| 32854 | G30B.00 | Acute posterolateral myocardial infarction |  |  |
| 34328 | G311300 | Refractory angina |  |  |
| 34633 | G34y.00 | Other specified chronic ischaemic heart disease |  |  |
| 34803 | G30y.00 | Other acute myocardial infarction |  |  |
| 35713 | G34yz00 | Other specified chronic ischaemic heart disease NOS |  |  |
| 36523 | G311.00 | Preinfarction syndrome |  |  |
| 36609 | G342.00 | Atherosclerotic cardiovascular disease |  |  |
| 38609 | G351.00 | Subsequent myocardial infarction of inferior wall |  |  |
| 39449 | G312.00 | Coronary thrombosis not resulting in myocardial infarction |  |  |
| 39546 | Gyu3000 | [X]Other forms of angina pectoris |  |  |
| 39655 | G311.12 | Impending infarction |  |  |
| 39693 | G31y200 | Subendocardial ischaemia |  |  |
| 40429 | G301000 | Acute anteroapical infarction |  |  |
| 41221 | G30y200 | Acute septal infarction |  |  |
| 41835 | G384.00 | Postoperative subendocardial myocardial infarction |  |  |
| 45809 | G350.00 | Subsequent myocardial infarction of anterior wall |  |  |
| 46017 | G30yz00 | Other acute myocardial infarction NOS |  |  |
| 46112 | G380.00 | Postoperative transmural myocardial infarction anterior wall |  |  |
| 46166 | G35X.00 | Subsequent myocardial infarction of unspecified site |  |  |
| 46276 | G381.00 | Postoperative transmural myocardial infarction inferior wall |  |  |
| 47637 | Gyu3300 | [X]Other forms of chronic ischaemic heart disease |  |  |
| 52517 | Gyu3.00 | [X]Ischaemic heart diseases |  |  |
| 54251 | G311z00 | Preinfarction syndrome NOS |  |  |
| 54535 | G33z100 | Stenocardia |  |  |
| 55137 | G311011 | MI - myocardial infarction aborted |  |  |
| 61072 | G311000 | Myocardial infarction aborted |  |  |
| 62626 | G30y100 | Acute papillary muscle infarction |  |  |
| 63467 | G306.00 | True posterior myocardial infarction |  |  |
| 66388 | G33z000 | Status anginosus |  |  |
| 68357 | G31y100 | Microinfarction of heart |  |  |
| 68401 | Gyu3200 | [X]Other forms of acute ischaemic heart disease |  |  |
| 68748 | G38z.00 | Postoperative myocardial infarction, unspecified |  |  |
| 72562 | G353.00 | Subsequent myocardial infarction of other sites |  |  |
| 96838 | Gyu3400 | [X]Acute transmural myocardial infarction of unspecif site |  |  |
| 99991 | Gyu3600 | [X]Subsequent myocardial infarction of unspecified site |  |  |
| 105479 | G39..00 | Coronary microvascular disease |  |  |
| 106812 | G383.00 | Postoperative transmural myocardial infarction unspec site |  |  |
| Heart Failure (QOF definition) |  |  |  |  |
| 398 | G580.00 | Congestive heart failure |  |  |
| 884 | G581.00 | Left ventricular failure |  |  |
| 2062 | G58..00 | Heart failure |  |  |
| 2906 | G580.11 | Congestive cardiac failure |  |  |
| 13188 | 662G.00 | Hypertensive treatm.changed |  |  |
| 4024 | G58z.00 | Heart failure NOS |  |  |
| 1223 | G58..11 | Cardiac failure |  |  |
| 21826 | 662F.00 | Hypertension treatm. started |  |  |
| 5942 | G581.13 | Impaired left ventricular function |  |  |
| 13189 | 662g.00 | New York Heart Association classification - class II |  |  |
| 12948 | 662H.00 | Hypertension treatm.stopped |  |  |
| 18853 | 662f.00 | New York Heart Association classification - class I |  |  |
| 19066 | 662h.00 | New York Heart Association classification - class III |  |  |
| 5255 | G581000 | Acute left ventricular failure |  |  |
| 32671 | G580100 | Chronic congestive heart failure |  |  |
| 10079 | G580.12 | Right heart failure |  |  |
| 9524 | G580.14 | Biventricular failure |  |  |
| 17278 | G58z.12 | Cardiac failure NOS |  |  |
| 23707 | G580000 | Acute congestive heart failure |  |  |
| 10154 | G580.13 | Right ventricular failure |  |  |
| 27964 | G582.00 | Acute heart failure |  |  |
| 27884 | G580200 | Decompensated cardiac failure |  |  |
| 23481 | G581.11 | Asthma - cardiac |  |  |
| 51214 | 662i.00 | New York Heart Association classification - class IV |  |  |
| 43618 | G581.12 | Pulmonary oedema - acute |  |  |
| 11424 | G580300 | Compensated cardiac failure |  |  |
| 22262 | G1yz100 | Rheumatic left ventricular failure |  |  |
| 12590 | G58z.11 | Weak heart |  |  |
| 101138 | G583.00 | Heart failure with normal ejection fraction |  |  |
| 94870 | G580400 | Congestive heart failure due to valvular disease |  |  |
| 104275 | G584.00 | Right ventricular failure |  |  |
| 101137 | G583.11 | HFNEF - heart failure with normal ejection fraction |  |  |
| 106897 | G583.12 |  |  |  |
| Ischaemic Stroke |  |  |  |  |
| 5363 | G64..11 | CVA - cerebral artery occlusion |  |  |
| 6155 | G64..13 | Stroke due to cerebral arterial occlusion |  |  |
| 33543 | G6X..00 | Cerebrl infarctn due/unspcf occlusn or sten/cerebrl artrs |  |  |
| 53745 | Gyu6400 | [X]Other cerebral infarction |  |  |
| 40758 | G6W..00 | Cereb infarct due unsp occlus/stenos precerebr arteries |  |  |
| 40053 | G671.00 | Generalised ischaemic cerebrovascular disease NOS |  |  |
| 39403 | G683.00 | Sequelae of cerebral infarction |  |  |
| 91627 | Gyu6300 | [X]Cerebrl infarctn due/unspcf occlusn or sten/cerebrl artrs |  |  |
| 94482 | Gyu6G00 | [X]Cereb infarct due unsp occlus/stenos precerebr arteries |  |  |
| 92036 | Gyu6600 | [X]Occlusion and stenosis of other cerebral arteries |  |  |
| 90572 | Gyu6500 | [X]Occlusion and stenosis of other precerebral arteries |  |  |
| CKD stages 3-5 (QOF) |  |  |  |  |
| 2994 | 7L1A100 | Peritoneal dialysis |  |  |
| 2996 | 7L1A200 | Haemodialysis NEC |  |  |
| 2997 | 7B00.00 | Transplantation of kidney |  |  |
| 5504 | 7B00z00 | Transplantation of kidney NOS |  |  |
| 5911 | ZV42000 | [V]Kidney transplanted |  |  |
| 8037 | 7L1B000 | Insertion of ambulatory peritoneal dialysis catheter |  |  |
| 11553 | SP08300 | Kidney transplant failure and rejection |  |  |
| 11745 | 7B00100 | Transplantation of kidney from live donor |  |  |
| 11773 | 7L1A.11 | Dialysis for renal failure |  |  |
| 12479 | 1Z13.00 | Chronic kidney disease stage 4 |  |  |
| 12585 | 1Z14.00 | Chronic kidney disease stage 5 |  |  |
| 18774 | TB00111 | Renal transplant with complication, without blame |  |  |
| 20073 | 7L1A000 | Renal dialysis |  |  |
| 22252 | ZV45100 | [V]Renal dialysis status |  |  |
| 23773 | 7L1B100 | Removal of ambulatory peritoneal dialysis catheter |  |  |
| 24361 | 7B00200 | Transplantation of kidney from cadaver |  |  |
| 26862 | 7B06300 | Exploration of renal transplant |  |  |
| 28158 | TB11.00 | Kidney dialysis with complication, without blame |  |  |
| 30709 | 7L1C000 | Insertion of temporary peritoneal dialysis catheter |  |  |
| 30756 | 7L1A500 | Continuous ambulatory peritoneal dialysis |  |  |
| 36442 | 7L1B.11 | Placement ambulatory dialysis apparatus - compens renal fail |  |  |
| 45160 | ZV56y11 | [V]Aftercare involving peritoneal dialysis |  |  |
| 46145 | ZV56011 | [V]Aftercare involving renal dialysis NOS |  |  |
| 46438 | SP05613 | [X] Peritoneal dialysis associated peritonitis |  |  |
| 48057 | K0B5.00 | Renal tubulo-interstitial disordrs in transplant rejectn |  |  |
| 48121 | 7B01500 | Transplant nephrectomy |  |  |
| 48639 | SP01500 | Mechanical complication of dialysis catheter |  |  |
| 54844 | U612200 | [X]Failure sterile precautions dur kidney dialys/other perf |  |  |
| 54990 | TB00100 | Kidney transplant with complication, without blame |  |  |
| 55151 | 7B00000 | Autotransplant of kidney |  |  |
| 59315 | SP07G00 | Stenosis of arteriovenous dialysis fistula |  |  |
| 60446 | Z919.00 | Care of haemodialysis equipment |  |  |
| 60498 | Z919300 | Reversing haemodialysis lines |  |  |
| medcode | readcode | readterm |  |  |
| 60743 | ZV56.00 | [V]Aftercare involving intermittent dialysis |  |  |
| 63038 | ZV56z00 | [V]Unspecified aftercare involving intermittent dialysis |  |  |
| 63488 | ZV56y00 | [V]Other specified aftercare involving intermittent dialysis |  |  |
| 63502 | Z91A.00 | Peritoneal dialysis bag procedure |  |  |
| 64828 | 7L1A600 | Peritoneal dialysis NEC |  |  |
| 66705 | 7B00111 | Allotransplantation of kidney from live donor |  |  |
| 66714 | TB11.11 | Renal dialysis with complication, without blame |  |  |
| 69266 | TA22000 | Failure of sterile precautions during kidney dialysis |  |  |
| 69760 | ZVu3G00 | [X]Other dialysis |  |  |
| 70874 | 7B00y00 | Other specified transplantation of kidney |  |  |
| 72004 | 7B01511 | Excision of rejected transplanted kidney |  |  |
| 72336 | Z919100 | Priming haemodialysis lines |  |  |
| 88597 | 7L1A400 | Automated peritoneal dialysis |  |  |
| 89924 | 7B00300 | Allotransplantation of kidney from cadaver, heart-beating |  |  |
| 93366 | 7B0F.00 | Interventions associated with transplantation of kidney |  |  |
| 95122 | 1Z1H.00 | Chronic kidney disease stage 4 with proteinuria |  |  |
| 95405 | 1Z1L.00 | Chronic kidney disease stage 5 without proteinuria |  |  |
| 95406 | 1Z1J.00 | Chronic kidney disease stage 4 without proteinuria |  |  |
| 95508 | 1Z1K.00 | Chronic kidney disease stage 5 with proteinuria |  |  |
| 96133 | 7B00400 | Allotransplantation kidney from cadaver, heart non-beating |  |  |
| 96184 | TA02000 | Accid cut,puncture,perf,h'ge - kidney dialysis |  |  |
| 96347 | 7A61900 | Ligation of arteriovenous dialysis fistula |  |  |
| 97587 | 1Z1J.11 | CKD stage 4 without proteinuria |  |  |
| 97683 | 1Z1L.11 | CKD stage 5 without proteinuria |  |  |
| 98364 | 7B00211 | Allotransplantation of kidney from cadaver |  |  |
| 99160 | 1Z1K.11 | CKD stage 5 with proteinuria |  |  |
| 99312 | 1Z1H.11 | CKD stage 4 with proteinuria |  |  |
| 104963 | K054.00 | Chronic kidney disease stage 4 |  |  |
| 105151 | K055.00 | Chronic kidney disease stage 5 |  |  |
| Diabetic retinopathy |  |  |  |  |
| 1323 | F420.00 | Diabetic retinopathy |  |  |
| 1411 | 3128100 | Fundoscopy abnormal |  |  |
| 1438 | F421000 | Unspecified background retinopathy |  |  |
| 2254 | F424100 | Central serous retinopathy |  |  |
| 2986 | F420200 | Preproliferative diabetic retinopathy |  |  |
| 3286 | F420100 | Proliferative diabetic retinopathy |  |  |
| 3822 | 2BB8.00 | O/E - vitreous haemorrhages |  |  |
| 3837 | F420400 | Diabetic maculopathy |  |  |
| 3914 | 2BB9.00 | O/E - retinal pigmentation |  |  |
| 4514 | 7270011 | Anterior vitrectomy |  |  |
| 6509 | C108700 | Insulin depressionendent diabetes mellitus with retinopathy |  |  |
| 6702 | F421300 | Hypertensive retinopathy |  |  |
| 6836 | 7271100 | Laser photocoagulation of retina for detachment |  |  |
| 7069 | F420000 | Background diabetic retinopathy |  |  |
| 7890 | F422.00 | Other proliferative retinopathy |  |  |
| 8595 | F42y600 | Retinal exudate or depressionosit |  |  |
| 8742 | 2BB5.00 | O/E - retinal haemorrhages |  |  |
| 9318 | 7272300 | Laser destruction of lesion of retina |  |  |
| 9339 | F421.00 | Other background retinopathy |  |  |
| 9835 | 2BBL.00 | O/E - diabetic maculopathy present both eyes |  |  |
| 10099 | F420300 | Advanced diabetic maculopathy |  |  |
| 10755 | F420600 | Non proliferative diabetic retinopathy |  |  |
| 10882 | F421400 | Exudative retinopathy |  |  |
| 11053 | F421800 | Retinal microaneurysms NOS |  |  |
| 11129 | 2BBQ.00 | O/E - left eye background diabetic retinopathy |  |  |
| 11433 | 2BBP.00 | O/E - right eye background diabetic retinopathy |  |  |
| 11626 | F420z00 | Diabetic retinopathy NOS |  |  |
| 11858 | 7270400 | Pars plana vitrectomy |  |  |
| 11874 | F422100 | Proliferative retinopathy due to sickle cell disease |  |  |
| 11912 | 5B4..11 | Retinal laser therapy |  |  |
| 13097 | 2BBT.00 | O/E - right eye proliferative diabetic retinopathy |  |  |
| 13099 | 2BBR.00 | O/E - right eye preproliferative diabetic retinopathy |  |  |
| 13101 | 2BBV.00 | O/E - left eye proliferative diabetic retinopathy |  |  |
| 13102 | 2BBW.00 | O/E - right eye diabetic maculopathy |  |  |
| 13103 | 2BBS.00 | O/E - left eye preproliferative diabetic retinopathy |  |  |
| 13106 | 2BB6.00 | O/E - retinal exudates |  |  |
| 13107 | 2BBn.00 | O/E - left eye clinically significant macular oedema |  |  |
| 13108 | 2BBX.00 | O/E - left eye diabetic maculopathy |  |  |
| 17262 | C109600 | Non-insulin-depressionendent diabetes mellitus with retinopathy |  |  |
| 17293 | 727..00 | Retina and other parts of eye operations |  |  |
| 17916 | F422011 | Retinopathy of prematurity |  |  |
| 18387 | C10E700 | Type 1 diabetes mellitus with retinopathy |  |  |
| 18496 | C10F600 | Type 2 diabetes mellitus with retinopathy |  |  |
| 18775 | 2BBO.00 | O/E - Laser photocoagulation scars |  |  |
| 19532 | 2BB4.00 | O/E - retinal microaneurysms |  |  |
| 19533 | 2BBY.00 | O/E - referable retinopathy |  |  |
| 22871 | C10EP00 | Type 1 diabetes mellitus with exudative maculopathy |  |  |
| medcode | readcode | readterm |  |  |
| 22967 | 2BBF.00 | Retinal abnormality - diabetes related |  |  |
| 25591 | C10FQ00 | Type 2 diabetes mellitus with exudative maculopathy |  |  |
| 25888 | 2BBm.00 | O/E - right eye clinically significant macular oedema |  |  |
| 27022 | 5B42.00 | Laser therapy - retinal lesion |  |  |
| 30477 | F420700 | High risk proliferative diabetic retinopathy |  |  |
| 31829 | F433100 | Solar retinopathy |  |  |
| 34455 | F421112 | Atheroscleritic retinopathy |  |  |
| 35659 | 2BB7.00 | O/E - retinal vascular prolif. |  |  |
| 36035 | F422y00 | Other specified other proliferative retinopathy |  |  |
| 36119 | F421111 | Arterosclerotic retinopathy |  |  |
| 36855 | 2BBG.00 | Retinal abnormality - non-diabetes |  |  |
| 36867 | 2BBa.00 | O/E- non-referable retinopathy |  |  |
| 38096 | F422z00 | Proliferative retinopathy NOS |  |  |
| 38161 | C108711 | Type I diabetes mellitus with retinopathy |  |  |
| 39457 | F421C00 | Other intraretinal microvascular abnormality |  |  |
| 40982 | F421z00 | Other background retinopathy NOS |  |  |
| 41049 | C108712 | Type 1 diabetes mellitus with retinopathy |  |  |
| 41229 | F421100 | Atherosclerotic retinopathy |  |  |
| 42762 | C109612 | Type 2 diabetes mellitus with retinopathy |  |  |
| 45145 | 2BB2.00 | O/E - retinal vessel narrowing |  |  |
| 45876 | F421200 | Renal retinopathy |  |  |
| 46068 | 7272500 | Panretinal laser photocoagulation to lesion of retina NEC |  |  |
| 47328 | 2BBk.00 | O/E - right eye stable treated prolif diabetic retinopathy |  |  |
| 48751 | 2BB3.00 | O/E - retinal A-V nipping |  |  |
| 49655 | C10F611 | Type II diabetes mellitus with retinopathy |  |  |
| 50656 | 2BBc.00 | O/E - No retinal laser photocoagulation scars |  |  |
| 52041 | 2BBl.00 | O/E - left eye stable treated prolif diabetic retinopathy |  |  |
| 52630 | 2BBo.00 | O/E - sight threatening diabetic retinopathy |  |  |
| 55026 | 7270B11 | Anterior vitrectomy |  |  |
| 58604 | C109611 | Type II diabetes mellitus with retinopathy |  |  |
| 65463 | F420800 | High risk non proliferative diabetic retinopathy |  |  |
| 66964 | F426500 | Pseudoretinitis pigmentosa |  |  |
| 69662 | F421G00 | Venostasis retinopathy |  |  |
| 72424 | 7270B00 | Vitrectomy using anterior approach |  |  |
| 86068 | 7272800 | Panretinal laser photocoagulation to lesion of retina |  |  |
| 88368 | 7270411 | Vitrectomy using pars plana approach |  |  |
| 93875 | C10E712 | Insulin depressionendent diabetes mellitus with retinopathy |  |  |
| 95343 | C10E711 | Type I diabetes mellitus with retinopathy |  |  |
| 96926 | FyuF700 | [X]Other proliferative retinopathy |  |  |
| 97894 | C10EP11 | Type I diabetes mellitus with exudative maculopathy |  |  |
| 100979 | 7272900 | Focal laser photocoagulation of retina |  |  |
| 101881 | 2BBr.00 | Impaired vision due to diabetic retinopathy |  |  |
| 102242 | 2BBs.00 | Retinal arteries silverwire |  |  |
| Neuropathy |  |  |  |  |
| 2342 | F372.12 | Diabetic neuropathy |  |  |
| 2790 | F367.00 | Peripheral neuropathy |  |  |
| 2925 | F375.00 | Alcoholic polyneuropathy |  |  |
| 3958 | F366.00 | Polyneuropathy |  |  |
| 5002 | F372.11 | Diabetic polyneuropathy |  |  |
| 6908 | F36yz00 | Other idiopathic peripheral neuropathy NOS |  |  |
| 7635 | F362.00 | Hereditary sensory neuropathy |  |  |
| 7795 | C106.12 | Diabetes mellitus with neuropathy |  |  |
| 8591 | F35z.11 | Peripheral neuropathy - hereditary or idiopathic |  |  |
| 9193 | F336.00 | Phantom limb syndrome |  |  |
| 10722 | F37..00 | Inflammatory and toxic neuropathy |  |  |
| 11544 | N242300 | Neuropathic pain |  |  |
| 11663 | M271100 | Neuropathic diabetic ulcer - foot |  |  |
| 14883 | F36z.00 | Hereditary or idiopathic peripheral neuropathy NOS |  |  |
| 14884 | F36y.00 | Other idiopathic peripheral neuropathy |  |  |
| 15481 | F37z.00 | Toxic or inflammatory neuropathy NOS |  |  |
| 16230 | C106.00 | Diabetes mellitus with neurological manifestation |  |  |
| 16491 | C106.13 | Diabetes mellitus with polyneuropathy |  |  |
| 18016 | F336000 | Phantom limb syndrome with pain |  |  |
| 18075 | F36..00 | Hereditary and idiopathic peripheral neuropathy |  |  |
| 18425 | C10FB00 | Type 2 diabetes mellitus with polyneuropathy |  |  |
| 18534 | F342400 | Ulnar neuropathy |  |  |
| 19454 | F374A00 | Polyneuropathy in uraemia |  |  |
| 22573 | C106z00 | Diabetes mellitus NOS with neurological manifestation |  |  |
| 24121 | F378.00 | Intercostal neuropathy |  |  |
| 24216 | F370100 | Postinfectious polyneuritis |  |  |
| 24222 | F376.00 | Polyneuropathy due to drugs |  |  |
| 24226 | F37z.11 | Polyneuropathy unspecified |  |  |
| 24355 | F374200 | Polyneuropathy in vitamin B deficiency |  |  |
| 24571 | F372200 | Asymptomatic diabetic neuropathy |  |  |
| 24694 | C108B00 | Insulin depressionendent diabetes mellitus with mononeuropathy |  |  |
| 28333 | C373200 | Familial neuropathic amyloid |  |  |
| 30537 | F373.00 | Polyneuropathy in malignant disease |  |  |
| 31551 | F37X.00 | Inflammatory polyneuropathy, unspecified |  |  |
| 31790 | F372.00 | Polyneuropathy in diabetes |  |  |
| 32527 | F368.00 | Hereditary motor and sensory neuropathy |  |  |
| 34268 | C10F200 | Type 2 diabetes mellitus with neurological complications |  |  |
| 35465 | F368100 | Hereditary motor and sensory neuropathy type II |  |  |
| 35537 | Fyu7C00 | [X] Polyneuropathy, unspecified |  |  |
| 35785 | F372100 | Chronic painful diabetic neuropathy |  |  |
| 36643 | N035.12 | Neuropathic arthritis |  |  |
| 37315 | F3y0.00 | Diabetic mononeuropathy |  |  |
| 38401 | F360z00 | Hereditary peripheral neuropathy NOS |  |  |
| 39317 | C106100 | Diabetes mellitus, adult onset, + neurological manifestation |  |  |
| 39528 | F360.00 | Hereditary peripheral neuropathy |  |  |
| 39858 | Fyu7B00 | [X]Inflammatory polyneuropathy, unspecified |  |  |
| 40751 | F374900 | Polyneuropathy in sarcoidosis |  |  |
| 41652 | F37y.00 | Other toxic or inflammatory neuropathy |  |  |
| 41716 | C108C00 | Insulin depressionendent diabetes mellitus with polyneuropathy |  |  |
| 42831 | C10E200 | Type 1 diabetes mellitus with neurological complications |  |  |
| 44095 | F371000 | Polyneuropathy in disseminated lupus erythematosus |  |  |
| 44512 | F364.00 | Idiopathic progressive polyneuropathy |  |  |
| 45081 | F37..11 | Toxic neuropathy |  |  |
| 45467 | C109B00 | Non-insulin depressionendent diabetes mellitus with polyneuropathy |  |  |
| 45919 | C109212 | Type 2 diabetes mellitus with neurological complications |  |  |
| 46301 | C10EC00 | Type 1 diabetes mellitus with polyneuropathy |  |  |
| 46937 | F365.00 | Neuropathy in association with hereditary ataxia |  |  |
| 47409 | C109B11 | Type II diabetes mellitus with polyneuropathy |  |  |
| 47465 | F371100 | Polyneuropathy in polyarteritis nodosa |  |  |
| 49146 | C108211 | Type I diabetes mellitus with neurological complications |  |  |
| 50527 | C10FB11 | Type II diabetes mellitus with polyneuropathy |  |  |
| 50813 | C109A11 | Type II diabetes mellitus with mononeuropathy |  |  |
| 52089 | F374300 | Polyneuropathy in diphtheria |  |  |
| 52283 | C108200 | Insulin-depressionendent diabetes mellitus with neurological comps |  |  |
| 54124 | F377.00 | Other toxic agent polyneuropathy |  |  |
| 55076 | Fyu7.00 | [X]Polyneuropathies & other disord of peripheral nerv syst |  |  |
| 55842 | C109200 | Non-insulin-depressionendent diabetes mellitus with neuro comps |  |  |
| 56159 | Z6P2100 | Control of phantom sensation technique |  |  |
| 56272 | F374.00 | Polyneuropathy in disease EC |  |  |
| 56910 | F368000 | Hereditary motor and sensory neuropathy type I |  |  |
| 57313 | F371.00 | Polyneuropathy in collagen vascular disease |  |  |
| 58758 | F374800 | Polyneuropathy in porphyria |  |  |
| 59903 | C106.11 | Diabetic amyotrophy |  |  |
| medcode | readcode | readterm |  |  |
| 61523 | C106y00 | Other specified diabetes mellitus with neurological comps |  |  |
| 61829 | C108212 | Type 1 diabetes mellitus with neurological complications |  |  |
| 62401 | F371200 | Polyneuropathy in rheumatoid arthritis |  |  |
| 62674 | C10FA00 | Type 2 diabetes mellitus with mononeuropathy |  |  |
| 63555 | F374z00 | Polyneuropathy in disease NOS |  |  |
| 66336 | F374000 | Polyneuropathy in amyloidosis |  |  |
| 67853 | C106000 | Diabetes mellitus, juvenile, + neurological manifestation |  |  |
| 67905 | C109211 | Type II diabetes mellitus with neurological complications |  |  |
| 68105 | C10EB00 | Type 1 diabetes mellitus with mononeuropathy |  |  |
| 68960 | F374500 | Polyneuropathy in hypoglycaemia |  |  |
| 69047 | F37y000 | Serum neuropathy |  |  |
| 71258 | F371z00 | Polyneuropathy in collagen vascular disease NOS |  |  |
| 72320 | C109A00 | Non-insulin depressionendent diabetes mellitus with mononeuropathy |  |  |
| 72922 | Fyu6B00 | [X]Other mononeuropathies of lower limb |  |  |
| 73337 | F374100 | Polyneuropathy in beriberi |  |  |
| 91741 | Fyu6C00 | [X]Other specified mononeuropathies |  |  |
| 91943 | C10EC11 | Type I diabetes mellitus with polyneuropathy |  |  |
| 93228 | Fyu1300 | [X]Paraneoplastic neuromyopathy and neuropathy |  |  |
| 93868 | Fyu6A00 | [X]Other mononeuropathies of upper limb |  |  |
| 95351 | C10FA11 | Type II diabetes mellitus with mononeuropathy |  |  |
| 96256 | F37y100 | Axonal sensorimotor neuropathy |  |  |
| 97306 | Fyu7200 | [X]Other specified polyneuropathies |  |  |
| 97449 | Fyu7000 | [X]Other hereditary and idiopathic neuropathies |  |  |
| 97479 | Fyu7100 | [X]Other inflammatory polyneuropathies |  |  |
| 97848 | A72x100 | Mumps polyneuropathy |  |  |
| 98616 | C10F211 | Type II diabetes mellitus with neurological complications |  |  |
| 99231 | C108B11 | Type I diabetes mellitus with mononeuropathy |  |  |
| 99855 | M271700 | Neuropathic foot ulcer |  |  |
| 100064 | F374600 | Polyneuropathy in mumps |  |  |
| 101311 | C10EC12 | Insulin depressionendent diabetes mellitus with polyneuropathy |  |  |
| 101735 | C10E212 | Insulin-depressionendent diabetes mellitus with neurological comps |  |  |
| 105825 | C373K13 | Familial amyloid polyneuropathy type III |  |  |
| 106103 | F368200 | Hereditary motor and sensory neuropathy type III |  |  |
| 107322 | Fyu6D00 | [X]Other mononeuropathies in diseases classified elsewhere |  |  |
| Hypertension (QOF) |  |  |  |  |
| 204 | G2...00 | Hypertensive disease |  |  |
| 799 | G20..00 | Essential hypertension |  |  |
| 351 | G20..11 | High blood pressure |  |  |
| 15377 | G200.00 | Malignant essential hypertension |  |  |
| 1894 | G201.00 | Benign essential hypertension |  |  |
| 4372 | G202.00 | Systolic hypertension |  |  |
| 83473 | G203.00 | Diastolic hypertension |  |  |
| 10818 | G20z.00 | Essential hypertension NOS |  |  |
| 3712 | G20z.11 | Hypertension NOS |  |  |
| 7329 | G24..00 | Secondary hypertension |  |  |
| 31755 | G240.00 | Secondary malignant hypertension |  |  |
| 73293 | G240z00 | Secondary malignant hypertension NOS |  |  |
| 57288 | G241.00 | Secondary benign hypertension |  |  |
| 51635 | G241z00 | Secondary benign hypertension NOS |  |  |
| 34744 | G244.00 | Hypertension secondary to endocrine disorders |  |  |
| 16059 | G24z.00 | Secondary hypertension NOS |  |  |
| 31387 | G24z000 | Secondary renovascular hypertension NOS |  |  |
| 42229 | G24zz00 | Secondary hypertension NOS |  |  |
| 69753 | Gyu2.00 | [X]Hypertensive diseases |  |  |
| 102458 | Gyu2000 | [X]Other secondary hypertension |  |  |
| Depression |  |  |  |  |
| medcode |  | readterm | depress_diag_cat |  |
| 324 |  | Depressive disorder NEC | 3.depression |  |
| 543 |  | [X]Depression NOS | 3.depression |  |
| 595 |  | Endogenous depression | 3.depression |  |
| 655 |  | Anxiety with depression | 3.depression |  |
| 1055 |  | Agitated depression | 3.depression |  |
| 1131 |  | Neurotic depression reactive type | 3.depression |  |
| 1533 |  | Brief depressive reaction | 3.depression |  |
| 2560 |  | Depressive psychoses | 3.depression |  |
| 2639 |  | Postnatal depression | 3.depression |  |
| 2923 |  | Puerperal depression | 3.depression |  |
| 2970 |  | [X]Depressive episode, unspecified | 3.depression |  |
| 2972 |  | Postviral depression | 3.depression |  |
| 3291 |  | [X]Depressive disorder NOS | 3.depression |  |
| 3292 |  | [X]Recurrent depressive disorder | 3.depression |  |
| 4323 |  | Chronic depression | 3.depression |  |
| 4639 |  | [X]Depressive episode | 3.depression |  |
| 4979 |  | [X]Postpartum depression NOS | 3.depression |  |
| 5879 |  | Agitated depression | 3.depression |  |
| 5987 |  | [X] Reactive depression NOS | 3.depression |  |
| 6482 |  | Recurrent depression | 3.depression |  |
| 6546 |  | Endogenous depression first episode | 3.depression |  |
| 6854 |  | [X]Other depressive episodes | 3.depression |  |
| 6932 |  | Endogenous depression - recurrent | 3.depression |  |
| 6950 |  | Endogenous depression first episode | 3.depression |  |
| 7011 |  | Single major depressive episode NOS | 3.depression |  |
| 7604 |  | [X]Single episode of reactive depression | 3.depression |  |
| 7737 |  | [X]Neurotic depression | 3.depression |  |
| 7749 |  | [X]Mild anxiety depression | 3.depression |  |
| 7953 |  | [X]Dysthymia | 3.depression |  |
| 8478 |  | Reactive depressive psychosis | 3.depression |  |
| 8584 |  | [X]Depressive neurosis | 3.depression |  |
| 8826 |  | [X]SAD - Seasonal affective disorder | 3.depression |  |
| 8851 |  | [X]Recurrent episodes of depressive reaction | 3.depression |  |
| 8902 |  | [X]Recurrent episodes of reactive depression | 3.depression |  |
| 9055 |  | [X]Single episode of depressive reaction | 3.depression |  |
| 9183 |  | Masked depression | 3.depression |  |
| 9211 |  | [X]Moderate depressive episode | 3.depression |  |
| 9667 |  | [X]Severe depressive episode without psychotic symptoms | 3.depression |  |
| 10290 |  | [X]Depressive personality disorder | 3.depression |  |
| 10455 |  | Depressive personality disorder | 3.depression |  |
| 10610 |  | Single major depressive episode | 3.depression |  |
| 10667 |  | [X]Mild depression | 3.depression |  |
| 10720 |  | [X]Atypical depression | 3.depression |  |
| 10825 |  | Seasonal affective disorder | 3.depression |  |
| 11055 |  | [X]Schizoaffective disorder, depressive type | 3.depression |  |
| 11252 |  | [X]Major depression, recurrent without psychotic symptoms | 3.depression |  |
| 11329 |  | [X]Endogenous depression without psychotic symptoms | 3.depression |  |
| 11717 |  | [X]Mild depressive episode | 3.depression |  |
| 11913 |  | [X]Mixed anxiety and depressive disorder | 3.depression |  |
| 12099 |  | [X]Severe depressive episode with psychotic symptoms | 3.depression |  |
| 12122 |  | Depression medication review | 3.depression |  |
| 12399 |  | Depression annual review | 3.depression |  |
| 13307 |  | [X]Postnatal depression NOS | 3.depression |  |
| 14709 |  | Recurrent major depressive episodes, moderate | 3.depression |  |
| 15099 |  | Recurrent major depressive episode | 3.depression |  |
| 15155 |  | Single major depressive episode, moderate | 3.depression |  |
| 15219 |  | Single major depressive episode, severe, without psychosis | 3.depression |  |
| 15220 |  | [X]Persistant anxiety depression | 3.depression |  |
| 16506 |  | Single major depressive episode, mild | 3.depression |  |
| 16632 |  | Prolonged depressive reaction | 3.depression |  |
| 16861 |  | [X]Recurrent severe episodes of psychotic depression | 3.depression |  |
| 17770 |  | Psychotic reactive depression | 3.depression |  |
| 18510 |  | [X]Single episode of psychogenic depression | 3.depression |  |
| 19054 |  | [X]Recurrent brief depressive episodes | 3.depression |  |
| 19696 |  | [X]Recurrent episodes of psychogenic depression | 3.depression |  |
| 20785 |  | [X]Post-schizophrenic depression | 3.depression |  |
| 21887 |  | Senile dementia with depression | 3.depression |  |
| 22806 |  | [X]Single episode major depression w'out psychotic symptoms | 3.depression |  |
| 23731 |  | [X]Endogenous depression with psychotic symptoms | 3.depression |  |
| 24112 |  | [X]Single episode of psychotic depression | 3.depression |  |
| 24117 |  | [X]Single episode of major depression and psychotic symptoms | 3.depression |  |
| 24171 |  | Recurrent major depressive episodes, severe, with psychosis | 3.depression |  |
| 25563 |  | Recurrent major depressive episode NOS | 3.depression |  |
| 25697 |  | Recurrent major depressive episodes, severe, no psychosis | 3.depression |  |
| 27491 |  | Atypical depressive disorder | 3.depression |  |
| 27677 |  | Presenile dementia with depression | 3.depression |  |
| 27759 |  | [X] Senile dementia, depressed or paranoid type | 3.depression |  |
| 28248 |  | [X]Prolonged single episode of reactive depression | 3.depression |  |
| 28677 |  | [X]Manic-depress psychosis,depressed type+psychotic symptoms | 3.depression |  |
| 28756 |  | [X]Seasonal depressive disorder | 3.depression |  |
| 28863 |  | [X]Single episode of reactive depressive psychosis | 3.depression |  |
| 29342 |  | Recurrent major depressive episodes, mild | 3.depression |  |
| 29451 |  | [X]Manic-depress psychosis,depressd,no psychotic symptoms | 3.depression |  |
| 29520 |  | [X]Recurrent depressive disorder, current episode moderate | 3.depression |  |
| 29527 |  | [D]Postoperative depression | 3.depression |  |
| 29784 |  | [X]Recurrent depressive disorder, current episode mild | 3.depression |  |
| 30405 |  | Depression interim review | 3.depression |  |
| 31757 |  | [X]Recurr severe episodes/psychogenic depressive psychosis | 3.depression |  |
| 32159 |  | Single major depressive episode, severe, with psychosis | 3.depression |  |
| 32941 |  | [X]Recurr severe episodes/major depression+psychotic symptom | 3.depression |  |
| 33469 |  | [X]Recurr depress disorder cur epi severe without psyc sympt | 3.depression |  |
| 34390 |  | Single major depressive episode, unspecified | 34390 |  |
| 35274 |  | [X]Schizoaffective psychosis, depressive type | 35274 |  |
| 35671 |  | Recurrent major depressive episodes, unspecified | 35671 |  |
| 36246 |  | Brief depressive reaction NOS | 36246 |  |
| 36616 |  | [X]Monopolar depression NOS | 36616 |  |
| 37764 |  | [X]Recurrent severe episodes/reactive depressive psychosis | 37764 |  |
| 41022 |  | [X]Schizophreniform psychosis, depressive type | 41022 |  |
| 41089 |  | Senile dementia with depressive or paranoid features NOS | 41089 |  |
| 41989 |  | [X]Single episode agitated depressn w'out psychotic symptoms | 41989 |  |
| 43292 |  | Arteriosclerotic dementia with depression | 43292 |  |
| 44300 |  | [X]Recurrent depressive disorder, unspecified | 44300 |  |
| 44674 |  | Senile dementia with depressive or paranoid features | 44674 |  |
| 44848 |  | Depression management programme | 44848 |  |
| 47009 |  | [X]Recurrent depress disorder cur epi severe with psyc symp | 47009 |  |
| 47731 |  | [X]Other recurrent depressive disorders | 47731 |  |
| 52678 |  | [X]Single episode of psychogenic depressive psychosis | 52678 |  |
| 56609 |  | [X]Single episode of masked depression NOS | 56609 |  |
| 59386 |  | [X]Single episode vital depression w'out psychotic symptoms | 59386 |  |
| 73991 |  | [X]Vital depression, recurrent without psychotic symptoms | 73991 |  |
| 98252 |  | [X]Major depression, moderately severe | 98252 |  |
| 98346 |  | [X]Major depression, mild | 98346 |  |
| 98414 |  | [X]Major depression, severe without psychotic symptoms | 98414 |  |
| 98417 |  | [X]Major depression, severe with psychotic symptoms | 98417 |  |

# ISAC APPLICATION FORM

| For ISAC use only | | |
| --- | --- | --- |
| Protocol No.  Submission date  (DD/MM/YYYY) | ...........................  ........................... | ***IMPORTANT***  *Please refer to the* ***guidance*** *for ‘****Completing the ISAC application form’*** *found on the CPRD website (*[*www.cprd.com/isac*](http://www.cprd.com/isac)*). If you have any queries, please contact the ISAC Secretariat at* [*isac@cprd.com*](mailto:isac@cprd.com)*.* |

| SECTION A: GENERAL INFORMATION ABOUT THE PROPOSED RESEARCH STUDY | | |
| --- | --- | --- |
| 1. Study Title**^§^** (*Please state the study title below)*   Ethnic inequalities in trajectories of cardio-metabolic risk factor control and outcomes of type two diabetes  *^§^Please note: This information will be published on the CPRD’s website as part of its transparency policy.* | | |
| 1. **Has any part of this research proposal or a related proposal been previously submitted to ISAC?**   Yes **^*^** No x  **If yes, please provide the previous protocol number/s below. Please also state in your current submission how this/these are related or relevant to this study.* | | |
| 1. **Has this protocol been peer reviewed by another Committee? (e.g. grant award or ethics committee)**   Yes**^*^**  No x  **If Yes, please state the name of the reviewing Committee(s) below and provide an outline of the review process and outcome as an Appendix to this protocol* ***:*** | | |
| 1. **Type of Study** (please tick all the relevant boxes which apply)  \| Adverse Drug Reaction/Drug Safety \|  \| Drug Effectiveness \| x \| \| --- \| --- \| --- \| --- \| \| Drug Utilisation \| *x* \| Pharmacoeconomics \|  \| \| Disease Epidemiology \| *x* \| Post-authorisation Safety \|  \| \| Health care resource utilisation \| *x* \| Methodological Research \|  \| \| Health/Public Health Services Research \|  \| Other^*^ \|  \|     *^*^If Other, please specify the type of study in the lay summary* | | |
| 1. **Health Outcomes to be Measured^§^**   **^§^***Please note:* *This information will be published on CPRD’s website as part of its transparency policy.*  Please summarise below the primary/secondary health outcomes to be measured in this research protocol:   \| - Diabetes Mellitus \| - Myocardial Infarction \| - Nephropathy (CKD) \| \| --- \| --- \| --- \| \| - Coronary Heart Disease \| - Heart Failure \| - Neuropathy \| \| - Stroke \| - Retinopathy \|  \| | | |
| 1. **Publication: This study is intended for** (please tick all the relevant boxes which apply)**:**   Publication in peer-reviewed journals x Presentation at scientific conference x  Presentation at company/institutional meetings x Regulatory purposes  Other**^*^** x  **If Other, please provide further information:*     Presentation/Dissemination at diabetes patient groups | | |
| SECTION B: INFORMATION ON INVESTIGATORS AND COLLABORATORS | | |
| 1. **Chief Investigator^§^**   Please state the full name, job title, organisation name & e-mail address for correspondence - see guidance notes for eligibility. Please note that there can only be one Chief Investigator per protocol.  Rohini Mathur, Assistant Professor, London School of Hygiene & Tropical Medicine, Rohini.mathur@lshtm.ac.uk  **^§^***Please note:* *The name and organisation of the Chief Investigator and will be published on CPRD’s website as part of its transparency policy*  CV has been previously submitted to ISAC **x CV number:** 316_15CESL  A new CV is being submitted with this protocol  An updated CV is being submitted with this protocol | | |
| 1. **Affiliation of Chief Investigator** (full address)   London School of Hygiene & Tropical Medicine, Keppel Street, London, WC1E 7HT, UK | | |
| 1. **Corresponding Applicant^§^**   Please state the full name, affiliation(s) and e-mail address below:  Rohini Mathur, London School of Hygiene & Tropical Medicine, [Rohini.mathur@lshtm.ac.uk](mailto:Rohini.mathur@lshtm.ac.uk)  **^§^***Please note:* *The name and organisation of the corresponding applicant and their organisation name will be published on CPRD’s website as part of its transparency policy*  Same as chief investigator **x**  CV has been previously submitted to ISAC  **CV number:**  A new CV is being submitted with this protocol  An updated CV is being submitted with this protocol | | |
| 1. **List of all investigators/collaborators^§^**   Please list the full name, affiliation(s) and e-mail address* of all collaborators, other than the Chief Investigator below:  **^§^***Please note: The name of all investigators and their organisations/institutions will be published on CPRD’s website as part of its transparency policy*  Other investigator: Liam Smeeth, London School of Hygiene & Tropical Medicine, liam.smeeth@lshtm.ac.uk  CV has been previously submitted to ISAC **x CV number:** 045_15CEPSL  Other investigator: Krishnan Bhaskaran, London School of Hygiene & Tropical Medicine, [Krishnan.bhaskaran@lshtm.ac.uk](mailto:Krishnan.bhaskaran@lshtm.ac.uk)  CV has been previously submitted to ISAC x **CV number:** 156_15CESL  Other investigator: Ruth Farmer, London School of Hygiene & Tropical Medicine, ruth.farmer@lshtm.ac.uk  CV has been previously submitted to ISAC x CV number: 222_17    Other investigator: Sophie V Eastwood, UCL Institute of Cardiovascular Sciences, sophie.eastwood@ucl.ac.uk  CV has been previously submitted to ISAC x CV number: 221_17  Other investigator: Nish Chaturvedi, UCL Institute of Cardiovascular Sciences, n.chaturvedi@ucl.ac.uk  CV has been previously submitted to ISAC x CV number: 220_17  **Please note that your ISAC application form and protocol* ***must*** *be copied to all e-mail addresses listed above at the time of submission of your application to the ISAC mailbox. Failure to do so will result in delays in the processing of your application.* | | |
| 1. **Conflict of interest statement***   Please provide a draft of the conflict (or competing) of interest (COI) statement that you intend to include in any publication which might result from this work  No competing interests to declare  **Please refer to the International Committee of Medical Journal Editors (ICMJE) for guidance on what constitutes a COI.* | | |
| 1. **Experience/expertise available**   Please complete the following questions to indicate the experience/ expertise available within the team of investigators/collaborators actively involved in the proposed research, including the analysis of data and interpretation of results.  **Previous GPRD/CPRD Studies** **Publications using GPRD/CPRD data**  None  1-3  > 3 x x | | |
| **Experience/Expertise available** | **Yes** | **No** |
| **Is statistical expertise available within the research team?**  *If yes, please indicate the name(s) of the relevant investigator(s)*  Krishnan Bhaskaran is a senior lecturer with a background in medical statistics.  Ruth Farmer is an expert in the use of Marginal Structural Models to deal with time depressionendent confounding, particularly amongst individuals with diabetes. | **x** |  |
| **Is experience of handling large data sets (>1 million records) available within the research team?**  *If yes, please indicate the name(s) of the relevant investigator(s)*  RM, KB, RF, and LS all have significant experience using the CPRD | **x** |  |
| **Is experience of practising in UK primary care available to or within the research team?**  *If yes, please indicate the name(s) of the relevant investigator(s)*  LS, SVE, and NC are all experienced in practicing UK primary care | **x** |  |
| 1. **References relating to your study**   Please list up to 3 references (most relevant) relating to your proposed study:  Bellary S, O’Hare JP, Raymond NT, Mughal S, Hanif WM, Jones A, et al. Premature cardiovascular events and mortality in south Asians with type 2 diabetes in the United Kingdom Asian Diabetes Study - effect of ethnicity on risk. Curr Med Res Opin 2010;26:1873–9.  Stratton IM, Adler AI, Neil HA, Matthews DR, Manley SE, Cull CA, et al. Association of glycaemia with macrovascular and microvascular complications of type 2 diabetes (UKPDS 35): Prospective observational study. BMJ 2000;321:405–12.  Tillin T, Hughes AD, Mayet J, Whincup P, Sattar N, Forouhi NG, et al. The relationship between metabolic risk factors and incident cardiovascular disease in Europeans, South Asians, and African Caribbeans: SABRE (Southall and Brent Revisited) - A prospective population-based study. J Am Coll Cardiol 2013;61:1777–86. | | |
| SECTION C: ACCESS TO THE DATA | | |
| 1. **Financial Sponsor of study^§^**   **^§^***Please note:* *The name of the source of funding will be published on CPRD’s website as part of its transparency policy*  Pharmaceutical Industry  *Please specify name and country:*  Academia  *Please specify name and country:*  Government / NHS  *Please specify name and country:*  Charity *Please specify name and country*  Other  *Please specify name and country:*  None x | | |
| 1. **Type of Institution conducting the research**   Pharmaceutical Industry *Please specify name and country:*  Academia x *Please specify name and country: LSHTM, UK*  Government Depressionartment *Please specify name and country:*  Research Service Provider *Please specify name and country:*  NHS *Please specify name and country:*  Other  *Please specify name and country:* | | |
| 1. **Data access arrangements**   The financial sponsor/ collaborator* has a licence for CPRD GOLD and will extract the data  The institution carrying out the analysis has a licence for CPRD GOLD and will extract the data** x  A data set will be provided by the CPRD^¥€^  CPRD has been commissioned to extract the data and perform the analyses^€^  Other:  *If Other, please specify:*  **Collaborators supplying data for this study must be named on the protocol as co-applicants.*  ***If data sources other than CPRD GOLD are required, these will be supplied by CPRD*  ^¥^*Please note that datasets provided by CPRD are limited in size; applicants should contact CPRD (*[*kc@cprd.com*](mailto:kc@cprd.com)*) if a dataset of >300,000 patients is required.*  ^€^*Investigators must discuss their request with a member of the CPRD Research team before submitting an ISAC application. Please contact the CPRD Research Team on +44 (20) 3080 6383 or email (*[*kc@cprd.com*](mailto:kc@cprd.com)*) to discuss your requirements. Please also state the name of CPRD Research team with whom you have discussed this request (provide the date of discussion and any relevant reference information):*  Name of CPRD Researcher Daniel Dedman Reference number (where available) OCR9190 Date of contact 16th Feb 2017 | | |
| 1. **Primary care data**   Please specify which primary care data set(s) are required)  Vision only (Default for CPRD studies Both Vision and EMIS^®^* x  EMIS^®^ only*    *Note: Vision and EMIS are different practice management systems. CPRD has traditionally collected data from Vision practice. Data collected from EMIS is currently under evaluation prior to wider release.*  **Investigators requiring the use of EMIS data* ***must*** *discuss the study with a member of the CPRD Research team before submitting an ISAC application*  Please state the name of the CPRD Researcher with whom you have discussed your request for EMIS data:  Name of CPRD Researcher Daniel Dedman Reference number (where available) OCR9190 Date of contact 16^th^ Feb 2017 | | |
| SECTION D: INFORMATION ON DATA LINKAGES | | |
| 1. **Does this protocol seek access to linked data**   Yes* x No  If No, please move to section E.  **Research groups which have not previously accessed CPRD linked data resources* ***must*** *discuss access to these resources with a member of the CPRD Research team, before submitting an ISAC application. Investigators requiring access to HES Accident and Emergency data, HES Diagnostic Imaging Dataset and PROMS data* ***must*** *also discuss this with a member of the CPRD Research team before submitting an ISAC application. Please contact the CPRD Research Team on +44 (20) 3080 6383 or email* [*kc@cprd.com*](mailto:kc@cprd.com) *to discuss your requirements* ***before*** *submitting your application.*  Please state the name of the CPRD Researcher with whom you have discussed your linkage request.  Name of CPRD Researcher Daniel Dedman Reference number (where available) OCR9190 Date of contact 16th Feb 2017  *Please note that as part of the ISAC review of linkages, your protocol may be shared - in confidence - with a representative of the requested linked data set(s) and summary details may be shared - in confidence - with the Confidentiality Advisory Group of the Health Research Authority.* | | |
| 1. **Please select the source(s) of linked data being requested^§^**   *^§^Please note: This information will be published on the CPRD’s website as part of its transparency policy.*   \| X ONS Death Registration Data \| MINAP (Myocardial Ischaemia National Audit Project) \| \| --- \| --- \| \| X HES Admitted Patient Care \| Cancer Registration Data* \| \| X HES Outpatient \| PROMS (Patient Reported Outcomes Measure)** \| \| HES Accident and Emergency \| CPRD Mother Baby Link \| \| HES Diagnostic Imaging Dataset \|  \|     Practice Level Index of Multiple Depressionrivation (Standard)  Practice Level Index of Multiple Depressionrivation (Bespoke)  X Patient Level Index of Multiple Depressionrivation***  Patient Level Townsend Score ***  Other**** *Please specify:*  **Applicants seeking access to cancer registration data must complete a Cancer Dataset Agreement form (available from CPRD). This should be submitted to the ISAC as an appendix to your protocol.* *Please also note that applicants seeking access to cancer registry data must provide consent for publication of their study title and study institution on the UK Cancer Registry website.*  ***Assessment of the quality of care delivered to NHS patients in England undergoing four procedures: hip replacement, knee replacement, groin hernia and varicose veins. Please note that patient level PROMS data are only accessible by academics*  **** ‘Patient level IMD and Townsend scores will not be supplied for the same study*  *****If “Other” is specified, please provide the name of the individual in the CPRD Research team with whom this linkage has been discussed.*  Name of CPRD Researcher Daniel Dedman Reference number (where available) OCR9190 Date of contact 16th Feb 2017 | | |
| 1. **Total number of linked datasets requested including CPRD GOLD**   Number of linked datasets requested *(practice/ ’patient’ level Index of Multiple Depressionrivation, Townsend Score or the CPRD Mother Baby Link should* ***not*** *be included in this count)*  4  *Please note: Where ≥5 linked datasets are requested, approval may be required from the Confidentiality Advisory Group (CAG) to access these data* | | |
| 1. **Is linkage to a local^¥^ dataset with <1 million patients being requested?**   Yes *  No x  **If yes, please provide further details:*  **^¥^** *Data from defined geographical areas i.e. non-national datasets.* | | |
| 1. **If you have requested one or more linked data sets, please indicate whether the Chief Investigator or any of the collaborators listed in question 5 above, have access to these data in a patient identifiable form (e.g. full date of birth, NHS number, patient post code), or associated with an identifiable patient index.**   Yes*  No x  ** If yes, please provide further details:* | | |
| 1. **Does this study involve linking to patient *identifiable* data (e.g. hold date of birth, NHS number, patient post code) from other sources?**   Yes  No x | | |
| SECTION E: VALIDATION/VERIFICATION | | |
| 1. **Does this protocol describe a purely observational study using CPRD data?**   Yes* x No**  ** Yes: If you will be using data obtained from the CPRD Group, this study does not require separate ethics approval from an NHS Research Ethics Committee.*  *** No: You may need to seek separate ethics approval from an NHS Research Ethics Committee for this study. The ISAC will provide advice on whether this may be needed.* | | |
| 1. **Does this protocol involve requesting any additional information from GPs?**   Yes*  No x  * *If yes, please indicate what will be required:*  Completion of questionnaires by the GP*^ψ^* Yes  No  Is the questionnaire a validated instrument? Yes  No  If yes, has permission been obtained to use the instrument? Yes  No  Please provide further information:  Other (please describe)  *^ψ^ Any questionnaire for completion by GPs or other health care professional must be approved by ISAC before circulation for completion.* | | |
| 1. **Does this study require contact with patients in order for them to complete a questionnaire?**   Yes*  No x  **Please note that any questionnaire for completion by patients must be approved by ISAC before circulation for completion.* | | |
| 1. **Does this study require contact with patients in order to collect a sample?**   Yes*  No x  ** Please state what will be collected:* | | |
| SECTION F: DECLARATION | | |
| 1. **Signature from the Chief Investigator**  - I have read the guidance on ‘***Completion of the ISAC application form****’* and ‘***Contents of CPRD ISAC Research Protocols***’ and have understood these; - I have read the submitted version of this research protocol, including all supporting documents, and confirm that these are accurate. - I am suitably qualified and experienced to perform and/or supervise the research study proposed. - I agree to conduct or supervise the study described in accordance with the relevant, current protocol - I agree to abide by all ethical, legal and scientific guidelines that relate to access and use of CPRD data for research - I understand that the details provided in sections marked with (^§^) in the application form and protocol will be published on the CPRD website in line with CPRD’s transparency policy. - I agree to inform the CPRD of the final outcome of the research study: publication, prolonged delay, completion or termination of the study.   Name: Rohini Mathur Date: 27^th^ Feb 2017 e-Signature (type name): Rohini Mathur | | |
