# Supplementary material for: Ethnic disparities in initiation and intensification of diabetes treatment in adults with type 2 diabetes in the UK, 1990–2017: A cohort study
Source: PLoS Med. 2020 May 15;17(5):e1003106. doi: 10.1371/journal.pmed.1003106 (PMC7228040; doi:10.1371/journal.pmed.1003106)
Supplement: S2 Table — (DOCX) [file pmed.1003106.s008.docx]

Table S6. Ethnic breakdown of study population according to the 16 categories of the UK Census

| Ethnic group | N | % |
| --- | --- | --- |
| 1. British | 74,241 | 30.90 |
| 2. Irish | 922 | 0.38 |
| 3. Other White | 14,688 | 6.11 |
| 4. White and Black Caribbean | 106 | 0.04 |
| 5. White and Black African | 74 | 0.03 |
| 6. White and Asian | 81 | 0.03 |
| 7. Other Mixed | 164 | 0.07 |
| 8. Indian | 3,554 | 1.48 |
| 9. Pakistani | 1,888 | 0.79 |
| 10. Bangladeshi | 737 | 0.31 |
| 11. Other Asian | 1,961 | 0.82 |
| 12. CaribbeanS | 1,462 | 0.61 |
| 13. African | 1,224 | 0.51 |
| 14. Other Black | 659 | 0.27 |
| 15. Chinese | 441 | 0.18 |
| 16. Other ethnic group | 1,724 | 0.72 |
| 17. Unknown | 136,364 | 56.75 |
| Total | 240,290 | 100.00 |

*Study population defined as individuals diagnosed with T2DM between 1990 and 2017 with at least one year of continuous registration prior to diagnosis
