# Supplementary material for: Ethnic disparities in initiation and intensification of diabetes treatment in adults with type 2 diabetes in the UK, 1990–2017: A cohort study
Source: PLoS Med. 2020 May 15;17(5):e1003106. doi: 10.1371/journal.pmed.1003106 (PMC7228040; doi:10.1371/journal.pmed.1003106)
Supplement: S3 Table — (DOCX) [file pmed.1003106.s009.docx]

Supplementary table S7: Baseline Characteristics for mixed/other and unknown groups compared to White

|  | | | | **White** | | **Mixed/Other** | | **Unknown** | |
| --- | --- | --- | --- | --- | --- | --- | --- | --- | --- |
| N | | | | 150754 | | 2794 | | 75258 | |
| Years of follow-up, (mean,SD) | | | | 77072 | (51.1) | 1269 | (45.4) | 34323 | (45.6) |
| Age at diagnosis (mean, SD) | | | | 63.4 | (13.2) | 56.8 | (13.4) | 62.7 | (13.5) |
| Gender, male, n (%) | | | | 82619 | (54.8) | 1520 | (54.4) | 42372 | (56.3) |
| Deprivation Quintile | | | |  |  |  |  |  |  |
| 1 (least deprived) , n (%) | | | | 27606 | (18.3) | 451 | (16.1) | 16168 | (21.5) |
| 2, n (%) | | | | 30092 | (20) | 415 | (14.9) | 11593 | (15.4) |
| 3, n (%) | | | | 33318 | (22.1) | 622 | (22.3) | 13724 | (18.2) |
| 4, n (%) | | | | 28989 | (19.2) | 662 | (23.7) | 17498 | (23.3) |
| 5 (most deprived), n (%) | | | | 30749 | (20.4) | 644 | (23) | 16275 | (21.6) |
| Smoking status | | | |  |  |  |  |  |  |
| Never smoker, n (%) | | | | 51565 | (34.2) | 1246 | (44.6) | 27213 | (36.2) |
| Current smoker, n (%) | | | | 23503 | (15.6) | 405 | (14.5) | 11007 | (14.6) |
| Ex-smoker, n (%) | | | | 47629 | (31.6) | 551 | (19.7) | 20022 | (26.6) |
| Missing, n (%) | | | | 28057 | (18.6) | 592 | (21.2) | 17016 | (22.6) |
| BMI | | | |  |  |  |  |  |  |
| BMI at diagnosis, kg/m2 (mean, SD) | | | | 31.7 | (6.1) | 29.9 | (5.9) | 31.5 | (6) |
| Underweight (,<20, <18.5 for SA) | | | | 1447 | (1) | 52 | (1.9) | 704 | (.9) |
| Normal weight (20-25, 18.4-23 for SA) | | | | 13560 | (9) | 405 | (14.5) | 6813 | (9.1) |
| Overweight (25-30, 23.5-27.5) | | | | 39826 | (26.4) | 835 | (29.9) | 19575 | (26) |
| Obese (>30, >27.5) | | | | 71664 | (47.5) | 1002 | (35.9) | 33855 | (45) |
| Missing | | | | 24257 | (16.1) | 500 | (17.9) | 14311 | (19) |
| HbA1c | | | |  |  |  |  |  |  |
| HbA1c at diagnosis % (mean, SD) | | | | 8 | (2.1) | 8.2 | (2.2) | 8.1 | (2.2) |
| HbA1c at diagnosis, IFCC (mean, SD) | | | | 63.6 | (23.2) | 66 | (24.4) | 64.9 | (24.4) |
| ≤7.5%, n (%) | | | | 64642 | (42.9) | 1159 | (41.5) | 29405 | (39.1) |
| 7.5-7.9%, n (%) | | | | 11191 | (7.4) | 253 | (9.1) | 5391 | (7.2) |
| 8.0-8.9%, n (%) | | | | 13291 | (8.8) | 241 | (8.6) | 6674 | (8.9) |
| ≥9.0%, n (%) | | | | 29594 | (19.6) | 611 | (21.9) | 15582 | (20.7) |
| Missing, n (%) | | | | 32036 | (21.3) | 530 | (19) | 18206 | (24.2) |
| Blood Pressure | | | |  |  |  |  |  |  |
| SBP at diagnosis (mean, SD) | | | | 140.3 | (18.8) | 135.8 | (19) | 140.7 | (19.2) |
| DBP at diagnosis (mean, SD) | | | | 80.9 | (10.9) | 81.1 | (10.5) | 81.3 | (11) |
| <140/90, n (%) | | | | 27185 | (19) | 425 | (16.2) | 14286 | (20.4) |
| <150/90, n (%) | | | | 20424 | (14.3) | 286 | (10.9) | 10701 | (15.3) |
| <130/80, n (%) | | | | 76154 | (53.2) | 1246 | (47.4) | 38982 | (55.8) |
| Missing, n (%) | | | | 7564 | (5) | 168 | (6) | 5392 | (7.2) |
| Co-morbidities & medications | | | |  |  |  |  |  |  |
| Any Macrovascular, n (%) | | | | 21669 | (14.4) | 221 | (7.9) | 8805 | (11.7) |
| Any Microvascular, n (%) | | | | 4685 | (3.1) | 59 | (2.1) | 1959 | (2.6) |
| Depression, n(%) | | | | 34246 | (22.7) | 590 | (21.1) | 14825 | (19.7) |
| On antihypertensive at diagnosis, n (%) | | | | 43427 | (28.8) | 554 | (19.8) | 19642 | (26.1) |
| On statin at diagnosis, n (%) | | | | 77072 | (51.1) | 1269 | (45.4) | 34323 | (45.6) |
| DM treatment Initiation characteristics | | | |  |  |  |  |  |  |
| Initiate < 1 year before diagnosis, n (%) | | | | 10654 | (7.1) | 216 | (7.7) | 5504 | (7.3) |
| Initiate in 12 months prior to diagnosis, n (%) | | | | 33034 | (21.9) | 775 | (27.7) | 16570 | (22) |
| Initiate within 90 days of diagnosis,n (%) | | | | 27804 | (18.4) | 532 | (19) | 13280 | (17.6) |
| Initiate >90 days after diagnosis, n (%) | | | | 45187 | (30) | 751 | (26.9) | 21890 | (29.1) |
| Non-initiators of DM treatment, n (%) | | | | 34075 | (22.6) | 520 | (18.6) | 18014 | (23.9) |
|  |  |  |  |  |  |  |  |  |  |
