# Supplementary material for: Ethnic disparities in initiation and intensification of diabetes treatment in adults with type 2 diabetes in the UK, 1990–2017: A cohort study
Source: PLoS Med. 2020 May 15;17(5):e1003106. doi: 10.1371/journal.pmed.1003106 (PMC7228040; doi:10.1371/journal.pmed.1003106)
Supplement: S4 Table — (DOCX) [file pmed.1003106.s010.docx]

Supplementary Table S8. Time to diabetes treatment initiation and intensification for white vs. other, mixed and unknown ethnic groups

|  | Initiation of non-insulin monotherapy | | | | Intensification to non-insulin combination therapy | | | | | Intensification to insulin therapy | | | |  |  |
| --- | --- | --- | --- | --- | --- | --- | --- | --- | --- | --- | --- | --- | --- | --- | --- |
|  | White | Other | Mixed | Unknown | White | | Other | Mixed | Unknown | White | Other | Mixed | Unknown |  |  |
| N eligible to initiate/intensify | 136540 | 1946 | 552 | 67869 | 105025 | | 1557 | 454 | 51417 | 55872 | 715 | 215 | 26478 |  |  |
| % who initiate/intensify at any time | 98.9 | 98.9 | 98.8 | 98.9 | 46.2 | | 38.5 | 39.7 | 44.4 | 20.9 | 14.4 | 20.0 | 18.1 |  |  |
| **Time to treatment initiation/intensification** |  |  |  |  |  | |  |  |  |  |  |  |  |  |  |
| Months to initiation/intensification, (Mean, SD) | 6.2 (33.5) | 3.4 (24.4) | 1.9 (20.6) | 6.6 (33.1) | 29.6 (45.1) | | 26.2 (42.2) | 24.4 (36) | 27.9 (43) | 45.5 (59.8) | 44.5 (60.7) | 50.8 (55.6) | 42.8 (58.5) |  |  |
| Relative risk vs. white (HR, 95% CI , p.val) |  | 1.05 (0.87,1.26) 0.594 | 1.18 (0.73,1.90) 0.512 | 0.89 (0.82,0.97) 0.008 |  | | 0.88 (0.77,1.02) 0.081 | 0.88 (0.69,1.12) 0.305 | 0.97 (0.93,1.01) 0.099 |  | 0.41 (0.28,0.61) <0.001 | 0.84 (0.53,1.33) 0.461 | 0.89 (0.82,0.96) 0.002 |  |  |
| **Between treatment characteristics** |  |  |  |  |  | |  |  |  |  |  |  |  |  |  |
| Mean HbA1c at diagnosis/start of follow-up period (%) | 8 (2.1) | 8.1 (2.2) | 8.2 (2.2) | 8.1 (2.2) | 8.6 (1.9) | | 8.6 (2) | 8.5 (2.1) | 8.8 (2) | 8.9 (1.8) | 9 (2) | 9.2 (2) | 9 (1.9) |  |  |
| Mean HbA1c closest to date of initiation/intensification (%) | 8.6 (2) | 8.7 (2) | 8.5 (2.2) | 8.8 (2) | 8.8 (1.7) | | 8.9 (1.8) | 8.8 (1.6) | 8.9 (1.7) | 10 (1.9) | 10.1 (2.2) | 9.7 (1.8) | 10.1 (2) |  |  |
| # HbA1c measurements between treatment stages (mean, SD) | 1.5 (2.9) | 1.3 (2.8) | 1.2 (2.6) | 1.4 (2.9) | 1.7 (3.1) | | 1.5 (2.9) | 1.2 (2.6) | 1.7 (3) | 1.4 (2.5) | 1.1 (2.3) | 1.1 (2.5) | 1.4 (2.6) |  |  |
| HbA1c count Rate Ratio (RR, 95%CI, p.val) |  | 0.96 (0.90,1.01) 0.133 | 0.96 (0.85,1.09) 0.547 | 1.00 (0.97,1.03) 0.946 |  | | 0.90 (0.76,1.06) 0.213 | 0.84 (0.64,1.10) 0.206 | 1.03 (0.98,1.09) 0.242 |  | 0.71 (0.55,0.93) 0.012 | 0.76 (0.50,1.16) 0.198 | 1.04 (0.95,1.14) 0.398 |  |  |
| # consultations between treatment stages (mean, SD) | 12 (27.6) | 9.6 (22.6) | 10.5 (28.9) | 10.8 (25.1) | 14.8 (29.9) | | 11.5 (24.4) | 11.9 (28.4) | 13.6 (27.2) | 11.3 (23.5) | 8 (17.2) | 8.9 (25.5) | 11 (22.4) |  |  |
| Consultation Rate Ratio (RR, 95%CI, p.val) |  | 0.96 (0.90,1.02) 0.162 | 0.98 (0.89,1.09) 0.742 | 0.95 (0.92,0.98) 0.001 |  | | 1.03 (0.82,1.28) 0.819 | 0.80 (0.56,1.15) 0.225 | 0.98 (0.92,1.04) 0.444 |  | 0.61 (0.47,0.80) <0.001 | 0.69 (0.45,1.06) 0.093 | 1.04 (0.93,1.16) 0.465 |  |  |
| * Population eligible for initiation excludes those on any DM drug 90 days prior to diagnosis | | | | | | | | | | | | | | | |
| *All models adjusted for age, sex, deprivation, year of diagnosis, HbA1c, BMI, micro- and macro- vascular co-morbidities, depression, consultation count, smoking status and medication count at start of follow-up period, and clustering by practice. Models for intensification to combination therapy and insulin additionally account for time since diagnosis. | | | | | | | | | | | | | | | |
| *Mean HbA1c and BMI taken as the latest in the 6 months prior to diagnosis (for model 1) initiation (for model 2) intensification 1 (for model 3) | | | | | | |  | | | | | | | | |
