# Supplementary material for: Ethnic disparities in initiation and intensification of diabetes treatment in adults with type 2 diabetes in the UK, 1990–2017: A cohort study
Source: PLoS Med. 2020 May 15;17(5):e1003106. doi: 10.1371/journal.pmed.1003106 (PMC7228040; doi:10.1371/journal.pmed.1003106)
Supplement: S5 Table — HbA1c, glycated haemoglobin. (DOCX) [file pmed.1003106.s011.docx]

Supplementary Table S11. Therapeutic inertia at initiation of non-insulin monotherapy using a cut-off of 6.5% for definition of raised HbA1c

|  |  | **N with any HbA1c>6.5% and 12 months follow-up** | **% experiencing treatment inertia at 12 months, % (n)** | **Months between first HbA1c>6.5% and intensification/end f-up, mean (SD)** | **Adjusted**  **Odds Ratio**  **OR (95%CI), p.val** |
| --- | --- | --- | --- | --- | --- |
| **Initiation of non-insulin monotherapy** | **White** | 34,213 | 42.0 (14,175) | 15.7 (21.1) | 1 |
|  | **South Asian** | 1,660 | 35.7 (582) | 13.3 (19.5) | 0.86 (0.67-1.12), 0.274 |
|  | **Black** | 550 | 34.2 (180) | 12.9 (20.3) | 0.86 (0.60,1.23), 0.405 |

*Regression model adjusts for age, gender, depressionrivation, HbA1c value, BMI value, smoking status, macro- and micro-vascular co-morbidities, and depression at start of follow-up, number of consultations, and medications at the start of each follow-up period, calendar year at follow-up start, and clustering by practice.
