# Supplementary material for: Ethnic disparities in initiation and intensification of diabetes treatment in adults with type 2 diabetes in the UK, 1990–2017: A cohort study
Source: PLoS Med. 2020 May 15;17(5):e1003106. doi: 10.1371/journal.pmed.1003106 (PMC7228040; doi:10.1371/journal.pmed.1003106)
Supplement: S6 Table — (DOCX) [file pmed.1003106.s012.docx]

Supplementary Table S12. Ethnic breakdown of individuals included and excluded from analysis of therapeutic inertia

|  | **Initiation of non-insulin monotherapy** | | **Intensification to non-insulin combination therapy** | | **Intensification to Insulin therapy** | |
| --- | --- | --- | --- | --- | --- | --- |
|  | Included  N=36,789 | Excluded  N=2,356 | Included  N=58,412 | Excluded  N=7,712 | Included  N=39,243 | Excluded  N=5,564 |
| **White, % (n)** | 93.9  (34,546) | 92.7  (2,351) | 93.0  (54,307) | 92.1  (6,603) | 93.0  (36,480) | 93.0  (5,173) |
| **South Asian, % (n)** | 4.6  (1,683) | 5.2  (133) | 5.3  (3,076) | 5.7  (603) | 5.3  (2,061) | 5.0  (279) |
| **Black, % (n)** | 1.5  (560) | 2.1  (52) | 1.8  (1,029) | 158  (2.2) | 1.8  (702) | 2.0  (112) |

*Included individuals are those with at least one HbA1c >7.5% and a minimum of 12 months follow-up post HbA1c measure.
