# Supplementary material for: Ethnic disparities in initiation and intensification of diabetes treatment in adults with type 2 diabetes in the UK, 1990–2017: A cohort study
Source: PLoS Med. 2020 May 15;17(5):e1003106. doi: 10.1371/journal.pmed.1003106 (PMC7228040; doi:10.1371/journal.pmed.1003106)
Supplement: S7 Table — (DOCX) [file pmed.1003106.s013.docx]

Supplementary Table S13. Full models for time to initiation/intensification and therapeutic inertia

Time to initiation

|  | | HR | | 95%CI | |
| --- | --- | --- | --- | --- | --- |
| analysis time when record ends |  | |  | |  |
| 0. White | 1 | | [1.00,1.00] | |  |
| 1. South Asian | 1.21^***^ | | [1.08,1.36] | |  |
| 2. Black | 1.29^*^ | | [1.05,1.59] | |  |
| 3. Other | 1.05 | | [0.87,1.26] | |  |
| 4. Mixed | 1.18 | | [0.73,1.90] | |  |
| 5. Unknown | 0.89^**^ | | [0.82,0.97] | |  |
| year | 1.15^***^ | | [1.14,1.16] | |  |
| HbA1c closest to diagnosis within 1 yr before or 3 months after diagnosis | 1.57^***^ | | [1.54,1.59] | |  |
| 1. Male | 1 | | [1.00,1.00] | |  |
| 2. Female | 1.07^***^ | | [1.04,1.11] | |  |
| IMD quintile=1 | 1 | | [1.00,1.00] | |  |
| IMD quintile=2 | 1.05 | | [0.93,1.17] | |  |
| IMD quintile=3 | 1.13^*^ | | [1.02,1.26] | |  |
| IMD quintile=4 | 1.07 | | [0.95,1.22] | |  |
| IMD quintile=5 | 1.20^**^ | | [1.06,1.37] | |  |
| Age at T2DM diagnosis | 0.99^***^ | | [0.99,0.99] | |  |
| macrovascular co-morbidities | 0.93^**^ | | [0.89,0.97] | |  |
| microvascular co-morbidities | 0.97 | | [0.89,1.05] | |  |
| depression=0 | 1 | | [1.00,1.00] | |  |
| depression=1 | 0.96 | | [0.92,1.00] | |  |
| never smoker (ref) | 1 | | [1.00,1.00] | |  |
| current smoker | 1.06^*^ | | [1.01,1.11] | |  |
| ex-smoker | 1.03 | | [0.99,1.07] | |  |
| BMI | 1.00^**^ | | [0.99,1.00] | |  |
| # medications in 6 months prior to diagnosis | 1.29^***^ | | [1.25,1.34] | |  |
| # consultations in 6 months prior to diagnosis | 0.99^***^ | | [0.99,1.00] | |  |
| N_sub |  | |  | |  |

Exponentiated coefficients; 95% confidence intervals in brackets

^*^ *p* < 0.05, ^**^ *p* < 0.01, ^***^ *p* < 0.001

Time to first intensification

|  | HR | 95%CI |
| --- | --- | --- |
| analysis time when record ends |  |  |
| 0. White | 1 | [1.00,1.00] |
| 1. South Asian | 0.80^***^ | [0.74,0.87] |
| 2. Black | 0.79^***^ | [0.70,0.90] |
| 3. Other | 0.88 | [0.77,1.02] |
| 4. Mixed | 0.88 | [0.69,1.12] |
| 5. Unknown | 0.97 | [0.93,1.01] |
| year | 0.97^***^ | [0.96,0.97] |
| HbA1c in 6 months before initiation- latest | 1.21^***^ | [1.20,1.22] |
| 1. Male | 1 | [1.00,1.00] |
| 2. Female | 0.93^***^ | [0.90,0.95] |
| IMD quintile=1 | 1 | [1.00,1.00] |
| IMD quintile=2 | 1.03 | [0.97,1.09] |
| IMD quintile=3 | 1.02 | [0.96,1.08] |
| IMD quintile=4 | 1.01 | [0.95,1.08] |
| IMD quintile=5 | 1.05 | [0.98,1.12] |
| Age at T2DM diagnosis | 0.98^***^ | [0.98,0.98] |
| macrovascular co-morbidities | 1.00 | [0.97,1.04] |
| microvascular co-morbidities | 1.06^*^ | [1.01,1.10] |
| depression=0 | 1 | [1.00,1.00] |
| depression=1 | 1.03^*^ | [1.00,1.07] |
| never smoker (ref) | 1 | [1.00,1.00] |
| current smoker | 1.03 | [1.00,1.06] |
| ex-smoker | 1.06^***^ | [1.03,1.09] |
| BMI | 1.01^***^ | [1.00,1.01] |
| # medications in 6 months prior to initiation | 1.05^***^ | [1.03,1.08] |
| # consultations in 6 months prior to initiation | 1.01^***^ | [1.00,1.01] |
| time_since_dx | 0.94^***^ | [0.94,0.95] |
| N_sub |  |  |

Exponentiated coefficients; 95% confidence intervals in brackets

^*^ *p* < 0.05, ^**^ *p* < 0.01, ^***^ *p* < 0.001

Time to second intensification

|  | HR | | 95%CI | |  |
| --- | --- | --- | --- | --- | --- |
| analysis time when record ends | |  | |  | |
| 0. White | | 1 | | [1.00,1.00] | |
| 1. South Asian | | 0.49^***^ | | [0.41,0.58] | |
| 2. Black | | 0.69^**^ | | [0.53,0.89] | |
| 3. Other | | 0.41^***^ | | [0.28,0.61] | |
| 4. Mixed | | 0.84 | | [0.53,1.33] | |
| 5. Unknown | | 0.89^**^ | | [0.82,0.96] | |
| year | | 0.93^***^ | | [0.92,0.93] | |
| HbA1c | | 1.17^***^ | | [1.15,1.19] | |
| 1. Male | | 1 | | [1.00,1.00] | |
| 2. Female | | 1.21^***^ | | [1.15,1.28] | |
| IMD quintile=1 | | 1 | | [1.00,1.00] | |
| IMD quintile=2 | | 0.97 | | [0.88,1.07] | |
| IMD quintile=3 | | 0.98 | | [0.89,1.09] | |
| IMD quintile=4 | | 1.01 | | [0.91,1.13] | |
| IMD quintile=5 | | 0.94 | | [0.84,1.04] | |
| Age at T2DM diagnosis | | 0.97^***^ | | [0.97,0.98] | |
| macrovascular co-morbidities | | 1.26^***^ | | [1.18,1.34] | |
| microvascular co-morbidities | | 1.13^***^ | | [1.05,1.21] | |
| intensification1depression=0 | | 1 | | [1.00,1.00] | |
| intensification1depression=1 | | 1.10^***^ | | [1.04,1.17] | |
| never smoker (ref) | | 1 | | [1.00,1.00] | |
| current smoker | | 1.24^***^ | | [1.17,1.32] | |
| ex-smoker | | 1.13^***^ | | [1.07,1.20] | |
| BMI | | 0.97^***^ | | [0.97,0.98] | |
| # medications in 6 months prior to intensification 1 | | 1.19^***^ | | [1.14,1.24] | |
| # consultations in 6 months prior to intensification 1 | | 1.02^***^ | | [1.02,1.02] | |
| time_since_dx | | 0.87^***^ | | [0.86,0.89] | |
| N_sub | |  | |  | |

Exponentiated coefficients; 95% confidence intervals in brackets

^*^ *p* < 0.05, ^**^ *p* < 0.01, ^***^ *p* < 0.001

Odds of treatment inertia time to initiation

|  | OR | 95%CI |
| --- | --- | --- |
| 0. White | 1 | [1.00,1.00] |
| 1. South Asian | 0.97 | [0.76,1.24] |
| 2. Black | 0.94 | [0.69,1.28] |
| 3. Other | 1.12 | [0.79,1.58] |
| 5. Unknown | 1.07 | [0.97,1.18] |
| Age at T2DM diagnosis | 1.01^**^ | [1.00,1.01] |
| 1. Male | 1 | [1.00,1.00] |
| 2. Female | 0.90^**^ | [0.83,0.97] |
| IMD quintile=1 | 1 | [1.00,1.00] |
| IMD quintile=2 | 0.98 | [0.87,1.10] |
| IMD quintile=3 | 0.95 | [0.84,1.08] |
| IMD quintile=4 | 0.90 | [0.79,1.02] |
| IMD quintile=5 | 0.86^*^ | [0.74,0.99] |
| hba1c_pre75 | 0.95^***^ | [0.94,0.97] |
| BMI | 1.01 | [1.00,1.01] |
| Never smoker | 1 | [1.00,1.00] |
| Current smoker | 1.00 | [0.91,1.11] |
| Ex-smoker | 0.92^*^ | [0.85,1.00] |
| Depression=0 | 1 | [1.00,1.00] |
| Depression=1 | 1.03 | [0.94,1.12] |
| any_macrovasc=0 | 1 | [1.00,1.00] |
| any_macrovasc=1 | 0.97 | [0.87,1.07] |
| any_microvasc=0 | 1 | [1.00,1.00] |
| any_microvasc=1 | 1.34^*^ | [1.07,1.67] |
| # consultations in 6 months prior to HbA1c>7.5% | 1.01 | [1.00,1.01] |
| # of oral meds in 6 months prior to hba1c 7.5 | 0.81^***^ | [0.76,0.86] |
| year | 0.96^***^ | [0.95,0.98] |
| time_since_dx | 1.00^***^ | [1.00,1.00] |
| / |  |  |
| lnsig2u | 0.17^***^ | [0.13,0.21] |
| N_sub |  |  |

Exponentiated coefficients; 95% confidence intervals in brackets ^*^ *p* < 0.05, ^**^ *p* < 0.01, ^***^ *p* < 0.001

Odds of treatment inertia time to intensification1

|  | OR | | 95%CI | |  |
| --- | --- | --- | --- | --- | --- |
| 0. White | | 1 | | [1.00,1.00] | |
| 1. South Asian | | 1.45^***^ | | [1.23,1.70] | |
| 2. Black | | 1.43^**^ | | [1.09,1.87] | |
| 3. Other | | 0.95 | | [0.74,1.22] | |
| 5. Unknown | | 1.13^**^ | | [1.05,1.23] | |
| Age at T2DM diagnosis | | 1.02^***^ | | [1.02,1.02] | |
| 1. Male | | 1 | | [1.00,1.00] | |
| 2. Female | | 0.94^*^ | | [0.89,1.00] | |
| IMD quintile=1 | | 1 | | [1.00,1.00] | |
| IMD quintile=2 | | 0.84^**^ | | [0.75,0.94] | |
| IMD quintile=3 | | 0.89^*^ | | [0.79,1.00] | |
| IMD quintile=4 | | 0.93 | | [0.83,1.04] | |
| IMD quintile=5 | | 0.82^**^ | | [0.73,0.92] | |
| hba1c_pre75 | | 0.84^***^ | | [0.83,0.86] | |
| BMI | | 1.01^***^ | | [1.00,1.01] | |
| Never smoker | | 1 | | [1.00,1.00] | |
| Current smoker | | 1.02 | | [0.95,1.10] | |
| Ex-smoker | | 0.90^**^ | | [0.84,0.96] | |
| Depression=0 | | 1 | | [1.00,1.00] | |
| Depression=1 | | 1.00 | | [0.93,1.06] | |
| any_macrovasc=0 | | 1 | | [1.00,1.00] | |
| any_macrovasc=1 | | 1.02 | | [0.94,1.12] | |
| any_microvasc=0 | | 1 | | [1.00,1.00] | |
| any_microvasc=1 | | 0.95 | | [0.84,1.09] | |
| # consultations in 6 months prior to HbA1c>7.5% | | 1.00 | | [1.00,1.01] | |
| # of oral meds in 6 months prior to hba1c 7.5 | | 0.87^***^ | | [0.83,0.92] | |
| year | | 1.02^**^ | | [1.01,1.04] | |
| time_since_dx | | 1.00^***^ | | [1.00,1.00] | |
| / | |  | |  | |
| lnsig2u | | 0.18^***^ | | [0.14,0.22] | |
| N_sub | |  | |  | |

Exponentiated coefficients; 95% confidence intervals in brackets ^*^ *p* < 0.05, ^**^ *p* < 0.01, ^***^ *p* < 0.001

Odds of treatment inertia time to intensification2

|  | OR | | | 95%CI | | | |  |
| --- | --- | --- | --- | --- | --- | --- | --- | --- |
| 0. White | | 1 | | | | [1.00,1.00] |  |  |
| 1. South Asian | | 2.68^***^ | | | | [1.89,3.80] |  |  |
| 2. Black | | 1.82^*^ | | | | [1.13,2.92] |  |  |
| 3. Other | | 2.43^*^ | | | | [1.20,4.95] |  |  |
| 5. Unknown | | 1.12 | | | | [0.98,1.29] |  |  |
| Age at T2DM diagnosis | | 1.03^***^ | | | | [1.03,1.04] |  |  |
| 1. Male | | 1 | | | | [1.00,1.00] |  |  |
| 2. Female | | 0.69^***^ | | | | [0.62,0.76] |  |  |
| IMD quintile=1 | | 1 | | | | [1.00,1.00] |  |  |
| IMD quintile=2 | | 1.03 | | | | [0.84,1.27] |  |  |
| IMD quintile=3 | | 0.99 | | | | [0.82,1.20] |  |  |
| IMD quintile=4 | | 0.99 | | | | [0.83,1.19] |  |  |
| IMD quintile=5 | | 1.08 | | | | [0.90,1.30] |  |  |
| hba1c_pre75 | | 0.68^***^ | | | | [0.66,0.70] |  |  |
| BMI | | 1.06^***^ | | | | [1.05,1.07] |  |  |
| Never smoker | | 1 | | | | [1.00,1.00] |  |  |
| Current smoker | | 0.88 | | | | [0.77,1.00] |  |  |
| Ex-smoker | | 1.02 | | | | [0.89,1.16] |  |  |
| Depression=0 | | 1 | | | | [1.00,1.00] |  |  |
| Depression=1 | | 0.89 | | | | [0.79,1.00] |  |  |
| any_macrovasc=0 | | 1 | | | | [1.00,1.00] |  |  |
| any_macrovasc=1 | | 0.83^**^ | | | | [0.72,0.95] |  |  |
| any_microvasc=0 | | 1 | | | | [1.00,1.00] |  |  |
| any_microvasc=1 | | 0.79^***^ | | | | [0.69,0.91] |  |  |
| # consultations in 6 months prior to HbA1c>7.5% | | 1.00 | | | | [0.99,1.01] |  |  |
| # of oral meds in 6 months prior to hba1c 7.5 | | 0.60^***^ | | | | [0.55,0.65] |  |  |
| year | | 0.92^***^ | | | | [0.90,0.94] |  |  |
| time_since_dx | | 1.00^***^ | | | | [1.00,1.00] |  |  |
| / | | |  | |  | | | |
| lnsig2u | | 0.19^***^ | | | | [0.13,0.28] |  |  |
| N_sub | |  | | | |  |  |  |

Exponentiated coefficients; 95% confidence intervals in brackets ^*^ *p* < 0.05, ^**^ *p* < 0.01, ^***^ *p* < 0.001
