# Supplementary material for: Ethnic disparities in initiation and intensification of diabetes treatment in adults with type 2 diabetes in the UK, 1990–2017: A cohort study
Source: PLoS Med. 2020 May 15;17(5):e1003106. doi: 10.1371/journal.pmed.1003106 (PMC7228040; doi:10.1371/journal.pmed.1003106)
Supplement: Attachment — Submitted filename: PLOS_Med_Response_to_reviewers_05022020.docx [file pmed.1003106.s014.docx]

**Requests from the editors:**

1. *Please confirm that author REF was not an employee of Boehringer when the study was being carried out, and that the company had no involvement in planning or carrying out the study.*

We can confirm that REF was not an employee of Boehringer at the time of the study and that the company had no involvement in the planning or conduct of the study.

2. *Please add the start and end years of the study to your title.*
    We have added the years to the study title which now reads: Ethnic differences in initiation and intensification of antidiabetic therapy and therapeutic inertia in adults with type 2 diabetes: A cohort study in the UK Clinical Practice Research Datalink 2004-2017
3. *To the "methods and findings" subsection of your abstract, please add additional demographic information on study participants: i.e., mean age and proportion male.*
   Mean age and proportion male have now been added to the methods and findings section of the abstract. The second sentence now reads: “The study cohort comprised 162,238 adults aged 18 and over with newly diagnosed T2DM between January 1990 and December 2017 (mean age 62.7 years, 55.2% male); 93% were of white ethnicity (n=150,754), 5% south Asian (n=8,139), 2.1% black (n=3,345).”
4. *Please add a few words to the abstract to summarize the approach to adjustment.*

The abstract now includes text on our approach to adjustment. The methods section now includes the text: Ethnic differences in time to initiation and intensification of antidiabetic treatment were estimated at three time points (initiation of non-insulin monotherapy, intensification to non-insulin combination therapy, and intensification to insulin therapy). Using multivariable Cox proportional hazards regression, we adjusted for factors a priori hypothesized to be associated with initiation and intensification; Namely, age, gender, deprivation, HbA1c, BMI, smoking status, co-morbidities at start of follow-up, number of consultations and medications at the start of each follow-up period, calendar year (to account for secular trends), and clustering by practice. Odds of experiencing therapeutic inertia (failure to intensify treatment within 12 months of HbA1c >7.5%/58 mmol/mol), were estimated using multivariable logistic regression adjusted for the same hypothesized confounders.

5. *We ask you to add p values alongside 95% CI where available.*
   P.values have now been added to all reported results in the text throughout the manuscript.
6. *Please remove elements of repetition from the presentation of your findings, e.g., "... Treatment inertia was higher in south Asian and Black groups at this stage relative to White participants (south Asian OR 1.72 ...; Black OR 1.61 ...)".*

We have removed repetition of results from the manuscript.

Page 12:

time to initiation was 21% faster south Asian groups (95%CI 8%-36%, p<0.001), and 29% faster in black groups relative to white (95%CI 5%-59%, p=0.017).

Page 13:

Both south Asian and black groups experienced greater therapeutic inertia than white groups at this stage (south Asian OR 1.40, 95%CI 1.20-1.65, p<001, Black OR 1.41, 95%CI 1.08-1.85, p=0.013).

Over 93% of all individuals on non-insulin combination therapy experienced therapeutic inertia when intensifying to insulin therapy. At this stage, ethnic minority groups were substantially more likely to experience therapeutic inertia than white groups (south Asian OR 2.68, 95%CI 1.90-3.80, p<0.001, Black OR 1.77, 95% CI 1.09-2.86, p=0.020)

7. *Please add a new final sentence to the "methods and findings" subsection of your abstract to summarize the study's main limitations.*

The methods and findings section of the abstract now has the additional text “Limitations of the study included variable quality and completeness of routinely recorded data, and a lack of information on whether prescriptions made by the GP were filled.”

8. *After the abstract, we ask you to add a new and accessible "author summary" section in non-identical prose. You may find it helpful to consult one or two recent PLOS Medicine research papers to get a sense of the preferred style.*

We have now added an author summary below the abstract which reads:

**Why was this study done?**

- In the UK, ethnic minority populations, particularly of south Asian and black African/Caribbean descent, have a higher risk of type 2 diabetes (T2DM) and related adverse outcomes such as cardiovascular disease than the white majority population.
- Timely and appropriate antidiabetic treatment can substantially reduce risk of adverse outcomes associated with T2DM.
- We sought to quantify ethnic differences in time to initiation and intensification of antidiabetic treatment amongst individuals newly diagnosed with T2DM to assess whether these clinically modifiable factors may contribute to ethnic differences in outcomes

**What did the researchers do and find?**

- We used routinely recorded data from general practices across the UK to identify people newly diagnosed with T2DM and compared how long it took to initiate and intensify antidiabetic treatment comparing people of white, south Asian, and Black ethnicity.
- We found that south Asian and black groups initiated antidiabetic treatment more quickly than white groups, but were slower to intensify to second- and third- line treatment regimens.

**What do these findings mean?**

- While time to initial treatment of type 2 diabetes was appropriate, ethnic disparities in subsequent longer-term treatment may contribute to the worse outcomes seen in ethnic minority populations in the UK.
- Interventions to improve timely and appropriate intensification of antidiabetic treatment are key to reducing disparities in the downstream adverse outcomes of T2DM.

9. *Early in the methods section of your main text, please state whether the study had a protocol or prespecified analysis plan (and if so attach the document[s] as supplementary files, referred to in the text). Please highlight analyses that were not prespecified.*

We have now added the following text to the methods section on page 10: “Analyses were conducted as specified in the scientific protocol submitted to the CPRD Independent Scientific Advisory Committee.” and attached the ISAC protocol for the study in the supplementary files.

10. *Please move the statement on ethics approval to the methods section of the main text.*
    We have now moved this statement to the methods section on page 11.
11. *Please avoid claims of "the first" (e.g., in the first paragraph of the discussion section), and where necessary add "to our knowledge" or similar.*
    We have now rephrased this sentence to read “To our knowledge, this is the first UK based study to examine the extent to which the appropriate and timely prescribing of antidiabetic treatment amongst individuals with type 2 diabetes differs by ethnic group.”
12. *In the discussion section of your main text, please restructure the text to create a discrete paragraph discussing limitations and add "This study had some limitations ..." or similar at the start.*
    We have now added the phrase “Several limitations may have influenced our findings” to indicate the beginning of our limitations section and we have collapsed all discussion of limitations into a single paragraph.
13. *Throughout your text, please reformat reference call-outs as follows: "... ethnic origin [1-3].".*
    The call-outs have now been reformatted to be in line with the PLOS Medicine style.
14. *Please substitute "sex" for "gender" as appropriate, throughout the paper.*
    This has now been changed throughout the manuscript.
15. *Please check all reference citations to ensure that they match journal style. Several references appear to need additional access information (e.g., reference 33); reference 31 seems to lack a journal name; reference 8 needs reformatting. Please remove all iterations of "[Internet]".*
    The formatting of the references has now been fixed to be in line with the PLOS medicine style- this has fixed the issues mentioned above.
16. *Please revise the attached RECORD checklist so that individual items are referred to by section (e.g., "Methods") and paragraph number rather than by page or line numbers, as the latter generally change in the event of publication.*

    We have now updated the RECORD checklist to refer to paragraphs within sections.
17. Please refer to the checklist in the methods section of your main text.

We have now referred to the RECORD guidelines at the end of the methods section. “All analyses were completed using Stata version 15 and reported according to the RECORD guidelines.”

***** Reviewer #1:**

*Thanks for the opportunity to review your manuscript. My role is as a statistical reviewer so my comments are focused on the study design, data, and analysis (and the presentation of these). I appreciate that detailed appendices on the derivation of the ethnicity information and the types of pharmacotherapy (and the other exposure information). This had headed off many of my usual questions I have from studies using GPRD and other similar routinely collected data sources, and will prove to be useful to anyone else attempting to do something similar to your own study. This study uses UK routinely collected data to estimate whether initiation and intensification of pharmacotherapy for T2DM differs according to ethnicity. From my own experience doing similar work I appreciate how complicated an analysis like this is, and I think that you have presented the details of this very clearly. I have some general queries around the study, and some more specific queries and points
following that.

General*

1. *Was a study protocol or statistical analysis plan developed for this study? It would helpful to see this if it was develop a-priori.*

Thank you for your kind comments about the study. Details of the analytical approach were included in the ISAC scientific approval and grant application to the Wellcome Trust, but a full SAP was not developed a-priori. We have now mentioned this in the methods and included the ISAC protocol in the supplementary materials.

Page 10 “Analyses were conducted as specified in the scientific protocol submitted to the CPRD Independent Scientific Advisory Committee.”

2. *Will diagnoses and pharmacotherapy provided by specialists (endocrinologists) appear in the dataset, and medicine given during hospital stays?*

As part of a wider shift from secondary to primary care management, the primary responsibility for all diabetes care lies with the general practitioner. [1] This includes initiation and intensification of medications. In rare instances of change of medication can be made via hospital, these changes are communicated to the GP and actioned accordingly.

While individuals in our study may have attended secondary or tertiary care for their diabetes care, this study did not have access to linked hospital data to explore this. However, even when linked hospital data are available, prescriptions and medications given during hospital stays are not part of the linked dataset made available to researchers.

3. *One of the key parts of the data that this study hinges on is accurate identification of patient's ethnicity, and the algorithm used to do this is clearly presented. This seems reasonable, and rather than give you 'death by sensitivity analyses' asking for the main results to be repeated by the various assumptions that could be made here, is it possible to quantify what the frequency of ethnicity in the study would change if a stricter definition was used where all records had to be consistent? Has there been any methods papers on the UK CPRD looking at the quality of the use of this variable? My own experience with other sources of routinely collected data is that this can wildly vary - although it certainly can be well recorded and reliable.*

Yes, we published a paper on the quality of ethnicity data in CPRD and hospital episode statistics in 2014 (Mathur et al. 2014, Quality and Usability of ethnicity data in UK-based primary care and hospital databases).

This work showed that amongst all people with multiple ethnicities coded on their primary care record, only 0.3% of individuals had codes which were discrepant (ie/ codes which belonged to more than one of the 5 categories of the Census- White, South Asian, Black, Mixed, Other)– thus consistency of ethnicity recording in the CPRD over time is extremely high, making it a stable indicator of an individual’s own ethnic identification over time.

4. *There is a fair amount of literature looking at T2DM management in South-East Asians in other countries - in this study these were put together into the 'other' category of ethnicity. Was there an insufficient number of self-identifying south-east Asian persons to consider these in this study? Is it possible to have a table that reports on the frequencies of the persons excluded as 'other' according to the 16 category ethnicity variable in the appendix?*

Thank you for this suggestion- we have now added a table to the supplementary material which reports the ethnic breakdown of the study population as per the 16 categories of the census (Supplementary table S1) and referred to this table at the beginning of the results section.

The full breakdown shows that 441 individuals identified as being of Chinese ethnicity, representing 0.18% of the study population. Other south East Asian groups fell into the other ethnic group as defined by the Census. Similarly, individuals of mixed ethnicity only accounted for 0.18% of the study population (n=425). The Other and mixed ethnic groups are both extremely heterogeneous, making it difficult to make clinically meaningful inferences about these populations with respect to diabetes care and outcomes.

The table is replicated here for reference.

Table S1. Ethnic breakdown of study population according to the 16 categories of the UK Census

| Ethnic group | N | % |
| --- | --- | --- |
| 1. British | 74,241 | 30.90 |
| 2. Irish | 922 | 0.38 |
| 3. Other White | 14,688 | 6.11 |
| 4. White and Black Caribbean | 106 | 0.04 |
| 5. White and Black African | 74 | 0.03 |
| 6. White and Asian | 81 | 0.03 |
| 7. Other Mixed | 164 | 0.07 |
| 8. Indian | 3,554 | 1.48 |
| 9. Pakistani | 1,888 | 0.79 |
| 10. Bangladeshi | 737 | 0.31 |
| 11. Other Asian | 1,961 | 0.82 |
| 12. Caribbean | 1,462 | 0.61 |
| 13. African | 1,224 | 0.51 |
| 14. Other Black | 659 | 0.27 |
| 15. Chinese | 441 | 0.18 |
| 16. Other ethnic group | 1,724 | 0.72 |
| 17. Unknown | 136,364 | 56.75 |
| Total | 240,290 | 100.00 |

*Study population defined as individuals diagnosed with T2DM between 1990 and 2017 with at least one year of continuous registration prior to diagnosis

5. *Has the use of self-identification to an ethnic group changed over time in the CPRD?*

The recording of self-identified ethnicity has remained consistent in UK primary care settings since its introduction. It is expected that ethnicity will be recorded in one of two ways: either the patient will be asked to fill in a drop-down checklist of similar categories to those of the 2001 Census, or the patient will be asked face to face by the general practitioner or other practice staff. This can take place during initial registration or consultation.

6. *Could you clarify how someone would be classified if they had never had valid record of ethnicity in the data?*

Individuals who never had a valid record of ethnicity were classified as ‘Unknown’.

Specific queries

7. *P6, Paragraph 2. What are the adjustments to the BMI categories?*

The WHO recommends BMI categories with lower cut-offs for individuals of south Asian ethnicity (Appropriate body-mass index for Asian populations and its

implications for policy and intervention strategies, Lancet, 2004). The adjustments for BMI recommended by the World Health organisation are as follows:

| BMI category | Standard cut-offs (kg/m^2^) | South Asian cut-offs (kg/m^2^) |
| --- | --- | --- |
| Underweight | <18.5 | <18.5 |
| Normal weight | 18.5-24.9 | 18.5-22.9 |
| Overweight | 25-29.9 | 23-27.4 |
| Obese | ≥30 | ≥27.5 |

We have now added text to the methods section to clarify this. “While BMI was considered as continuous covariate in the analytical models, descriptive statistics categorized BMI according to the standard categories of underweight, normal weight, overweight, and obese, with adjustments for south Asian ethnicity as recommended by the WHO, which classify normal weight as between 18.5-22.9 kg/m2, overweight as between 23-27.4 kg/m2 and obesity as 27.5 kg/m2 or over for this ethnic group.”

In response to the comments made by Reviewer 3, we are now treating BMI as a continuous variable in all analyses and only report the breakdown by category in table 1.

8. *P6, Paragraph 3. How are participants who move from one form of therapy to a less intense form of therapy, and then to another form of therapy treated? i.e. Monotherapy to none, none to combination therapy?*

Individuals who stayed on monotherapy would be included in the first analysis looking at time from diagnosis to initiation of monotherapy. They would then enter the second analysis looking at time to intensification with combination therapy - but would censored at the end of follow-up (death, leaving the CPRD, last data collection) having not experienced the outcome of interest. They would not enter the third analysis looking at time from combination therapy to insulin therapy.

For individuals who started monotherapy and then stopped - all the time post monotherapy initiation would be included in the analysis looking at time from monotherapy to combination therapy – as we did not explicitly model stopping.

People who moved straight from no therapy to combination therapy would have contributed follow-up time to the first model looking at time to initiation with monotherapy and would have been censored at the end of follow-up or date of initiating combination therapy, having not experienced the outcome of interest (initiation with monotherapy). As only people who had initiated monotherapy were eligible for inclusion in the models looking at time to intensification with combination, these individuals would not contribute to any further analyses.

We have described this in the methods section “For individuals who did not initiate or intensify with the drug of interest during the follow-up period, follow-up time was censored at the earliest of; date of intensifying to a different drug, death, transferring out of the practice, or last data collection. For example, individuals who initiated with a treatment other than non-insulin monotherapy were censored at the date the alternative treatment was commenced, and individuals who intensified directly from non-insulin monotherapy to insulin were censored at the date of insulin initiation.”

9. *Is the CPRD based on prescription or dispensing records?*

CPRD data are based on prescription records rather than dispensing records- so we can identify when prescriptions are issued by the GP, but not whether they are picked up by the individual. We have now added text about this to the limitations section of the discussion which has the additional sentence “While the CPRD captures prescriptions made by the general practitioner, it does not hold any information on dispensing data, thus we cannot know for certain whether prescriptions are filled or taken by the individual.”

10. *P7. Paragraph 4. Are non-pharmacotherapy periods considered to be a 'drug regime'?*

For the purposes of this study, non-pharmacotherapy was not considered as a drug regime. We have just published a study examining ethnic differences in time to initiation of non-pharmacological diabetes management, and found that non-white groups were offered structured diabetes management and clinical risk assessments (the NHS health check) more quickly than white groups.[2]

We have now added a comment about this in the discussion (at the time of original submission the above article was not yet published. “Correspondingly, our previous study examining ethnic differences in management of type 2 diabetes around the time of diagnosis found that that south Asian and black groups were offered non-pharmacological interventions (structured diabetes management and risk assessments) more quickly than white groups.”

11. *P7, Paragraph 5. What were the levels in the multilevel Cox model? Is this to account for multiple treatment periods (i.e. none -> mono, mono -> combination) within the same individual? [I've noticed this is clarified below, but it would be helpful to explain the levels here as well] Were proportional hazards between levels of covariates checked, and how was this done?*

The multiple levels of the survival models are to account for the clustering individuals within general practices. Furthermore, the multilevel extension of the Cox type survival analysis model is actually fully parametric, rather than semi-parametric. We used the mestreg command in Stata and assumed an exponential baseline hazard function. There is no postestimation command for the mestreg model which allows estimation of residuals to test for proportional hazards. We have now clarified this in the statistical analysis part of the methods section.

“Multilevel multivariable proportional hazard model assuming an exponential baseline hazard function was employed to identify ethnic differences in time to initiation and intensification of glucose-lowering treatment whilst accounting for the clustering of individuals within general practices.”

12. *P8, Paragraph 3. If a covariate (e.g. microvascular complications) changes during a treatment period, how is this accounted for?*

The status of covariates was estimated at baseline only for each timepoint. For initiation with monotherapy, covariate status was taken in the 12 months prior to diagnosis. Baseline covariates for intensification with combination therapy were taken from the 6 months prior to initiation with monotherapy. Baseline covariates for intensification with insulin were taken from the 6 months prior to initiation of combination therapy.

13. *P8, Paragraph 4. Were there any checks of overdispersion with the Poisson regression model?*

We have now checked the Poisson models and found evidence of overdispersion. We have updated our analysis of our count outcomes (count of consultations/hba1c measures) using the negative binomial regression instead. We have updated our methods to specify this and updated the results and reporting accordingly.

“Due to ovedispersion in the Poisson model, multilevel multivariable negative binomial regression was used to estimate ethnic differences in the count of HbA1c measurements and consultations between treatment stages adjusted for all hypothesized confounders. “

14. *P11, Paragraph 2/3. I'd also like to see cumulative incidence plots of this if possible (in an appendix is fine), and the median time to intensification (i.e. the time when 50% of those able to receive intensification received it.*

Thank you for this suggestion- we have now added cumulative incidence plots to the appendix in supplementary figure S3.

Your comment also helped us spot an error in Table 2. In the section “Time to treatment initiation/intensification”, the row which reports “Months to initiation/intensification (Mean, SD)” is in fact reporting Median and IQR – as requested. We have now updated reporting of these figures in the results section (page 12).

***** Reviewer #2:**

*The manuscript by Mathur et al deals with a very relevant issue regarding ethnic differences in the implementation of antidiabetic treatment in real-world practice. The authors used the well-known CPRD database to explore the potential ethnic differences in initiation and intensification of hypoglycemic therapy in type 2 diabetes in the UK. The methodology used is sound and the paper well written and easy to read. The findings are relevant for daily clinical practice and for future research in this field.
This reviewer would like the authors to address the following issues:*

1. *- In the Abstract, under Conclusions, the authors include a statement on delays in treatment intensification (second sentence). Actually, delays may be related to many factors and I would recommend rephrasing the sentence or delete it.*

Many thanks for taking the time to review our paper, and for your kind comments. We have rephrased the sentence in question to help clarify that that delays may be related to other factors. The sentence now reads: ‘Reasons for delays in treatment intensification are multifactorial, and may, in part, be related to poorer long-term monitoring of risk factors in non-white groups.’

2. *- Under Methods, it is not clear whether the information available on drug use is based on prescription by physicians or on pharmacy dispensation. This is relevant as information on the latter is a better proxy to adherence. In addition, this is an issue that deserves a comment in the discussion.*

Thank you for this suggestion. We have now clarified the source of information in the methods and made mention of this in the discussion.

Methods: Glucose-lowering treatment was identified from GP prescribing data and categorized into six classes

Discussion: “While the CPRD captures prescriptions made by the general practitioner, it does not hold any information on dispensing data, thus we cannot know whether prescriptions are filled or taken by the individual.”

3. *Also, under Methods, please explain the method used to handle missing data.*

We have now added a sentence to the methods section on page 10 ‘Due to the fact that routinely recorded data in primary care electronic health records are likely to be missing not at random, multiple imputation of missing data was not considered appropriate as the assumption of missing at random is unlikely to be met. As such, a complete case analysis approach was employed.[3]

4. *- In the section on Results, first paragraph, the sum of percentages provided sum up to 100.1%. Please, check this. Also, please, check the percentages given under 'Patterns of glucose-lowering treatment'.*

Thank you for this comment- we have now made our reporting of the percentages more accurate in these parts of the results.

First para: “The ethnic breakdown of the study cohort was 92.9% white (n=150,754), 5.0% south Asian (n=8,139), 2.1% black (n=3,345)”

Patterns of glucose-lowering treatment: Overall, 36.2% of individuals remained on non-insulin monotherapy for the entire study period, 26.0% intensified from non-insulin monotherapy to non-insulin combination therapy, and 6.8% intensified from monotherapy to combination therapy to insulin therapy. Individuals with treatment patterns differing from guidelines included those who initiated with non-insulin combination therapy (3.5%) or insulin therapy (1.2%); those who intensified from non-insulin monotherapy directly to insulin (1.8%); and those who initiated with combination therapy and intensified to insulin (1.0%); 23.8% of the study population did not initiate any glucose-lowering treatment during the study period (Figure 2).

5. *Although the duration of diabetes is most probably not different among groups, this information is of interest for the reader.*

Thank you very much for this useful suggestion- we have now added a row to our results table 2 for ‘Duration of diabetes at start of follow-up period’ for each of the three time periods of interest: Initiation of non-insulin monotherapy, intensification to non-insulin combination therapy, intensification to insulin therapy.

6. *- The authors included the number of consultations which is different between groups. However, it is important to know whether the difference may be due to non-attendance to the planned appointments. Do the authors have information on adherence to planned follow-up consultations. This may be also an indicator of adherence to the therapeutical plan; this may clearly influence the outcomes of the study.*

For this study, we had access to information on consultations attended, but not on whether the individuals did not attend a planned follow-up consultation. This is an important issue and we have now added text about this to our limitations section of the discussion. The additional text reads: “Finally, we were unable to explore ethnic differences in rates of non-attendance to planned appointments, which may be related to ethnic differences in adherence to therapeutic regimes and compliance with diabetes management plans.”

7. *- This reviewer understands that the data analyzed are those that correspond to primary care diabetes management. Do the authors have information on access of the subjects to secondary/tertiary care of the different ethnic groups? Were any subjects managed at this level for their diabetes?*

As part of a wider shift from secondary to primary care management, the vast majority of routine care for T2DM is provided in primary care settings. This includes initiation and intensification of medications. In rare instances of change of medication can be made via hospital, these changes are communicated to the GP and actioned accordingly.

While individuals in our study may have attended secondary or tertiary care for their diabetes care, this study did not have access to linked hospital data to explore this. However, even when linked hospital data are available, prescriptions and medications given during hospital stays are not part of the linked dataset made available to researchers.

8. *- There are several factors known to affect the outcomes measured in this study, both on the patient's and on the professional's side. One of them is on the subject with diabetes, i.e. depressive disorders; do the authors have data on differences among ethnic groups in the prevalence of this co-morbidity? This would be relevant as a confounding variable.*

Thank you for this suggestion, we do have access to data on coded diagnoses for depression and have now included it as a covariate in our models and reported its distribution in table 1.

9. *– Other confounding variables known to affect the outcomes are* *cultural differences in perception of the disease, perception of the impact on daily life (especially for insulin) and communication barriers between patients and health care professionals (very likely to affect treatment implementation). These variables have been shown to impact on treatment outcome and probably deserve a comment in the discussion.*

Thank you for this suggestion- we agree that these factors you have mentioned are all very important and have now included more text about this in the discussion section.

“Intensification to insulin is particularly challenging, due to the complexity of administration, the level of instruction required, and patient concerns around the use of injectable treatments. Such challenges may be further exacerbated by cultural and language barriers on both the patient [4,5] and provider side[6], potentially explaining excessive delays in treatment intensification amongst ethnic minority groups.”

10. *In the Discussion, under ‘Unanswered questions and future research’, I do not see the reason to specifically mention the CAROLINA trial. Please, explain.*

The CAROLINA trial was mentioned as at the time of writing the paper it had just published results of a comparative effectiveness study comparing antidiabetic regimens with respect to cardiovascular outcomes. Such work has not been undertaken in a UK context and would be valuable to replicate and extend as next steps in this area of research. We have now removed this reference.

****** Reviewer #3:***
*Thank you for the opportunity to review this review this article which uses the UK primary care electronic healthcare records to assess the association between Type 2 diabetes glucose lowering treatment intensification and ethnicity. The article shows that despite similar initial treatment intensification there appears to be substantially more inertia in initiating later glucose lowering therapies in those of Black and South Asian ethnic minority background, associated with reduced follow up and HBA1c monitoring.

The article is well written and addresses a question of some importance with (to my non methodologist eyes) robust analysis. However one factor notably missing is an attempt to understand/explain these differences, or relate them to findings in other disease areas.*

1. *The finding of differences later in care is at odds with that of initial treatment and it would be helpful to consider this further. Within the limitations of the dataset is there scope here to start to shed some light on cause of this variation, that might therefore help target action to address these imbalances? For instance how does this variation alter once reduced follow up/monitoring is taken into account - if a large effect this might point to differences in offering or attending following up being key areas to address, rather than patient or physician willingness to accept or prescribe more intense therapy.*

Thank you for this comment, we have now updated our regression models to account for the number of consultations prior to initiation/intensification to serve as an indicator of reduced follow-up/monitoring. Our estimates remain close to those reported in the original manuscript, with the direction of association and interpretation remaining unchanged, suggesting that reduced monitoring is one aspect of a complex and multifactorial decision making process between the individual and healthcare provider. We have added text to the discussion around concerns around intensification of insulin, and potential cultural/language barriers which may exacerbate existing delays in treatment intensification.

2. *Is there scope to assess adherence (at least in terms of prescription pick up)? Poor adherence to existing therapy can be a reason for not adding additional therapy.*

In UK primary care records, it is not possible to assess prescription pick up or adherence. We have now added this as a limitation of the study in the discussion section.

3. *It may also be informative to break down the effect adjusting for confounders in the same way as other authors in related fields have done (for example see figure 3 of Nanna et al JAMA cardiology 2018 PMID: 29898219).*

Thank you very much for this suggestion. We have provided tables reporting the complete breakdown of confounders for the models examining time to treatment initiation and intensification, and odds of therapeutic inertia in the appendix.

4. *On brief search it appears to me that there may be substantial related work in other fields relevant to this exact issue including in management of hypertension and treatment of lipids - it is unlikely that an issue with titrating diabetes therapy exists (or is best addressed) in isolation, so it would be helpful to at least put this in context of some of the work in other conditions in the discussion.*

We agree with the reviewer that the issues of therapeutic inertia are not unique to diabetes alone and have substantial overlap with approaches to therapeutic management of other long-term conditions. As suggested, we have added contextual information around other similar conditions and the importance of health beliefs in the discussion section.

“Considering the wider spectrum of cardio-metabolic disease, therapeutic inertia remains a substantial problem in the management of blood pressure and lipids. As with diabetes, causes for inertia are multifactorial, stemming from patient and provider, healthcare system, and policy factors. Recent reviews have highlighted the importance of education around clinical guidelines for both clinicians and patients, the use multi-disciplinary clinical teams, and the importance of quality improvement initiatives such as pay-for-performance schemes.[7,8]”

5. *It would also be helpful comment on possible explanation and related work - for example differences in statin use by ethnicity appear to be heavily influenced by health beliefs (see Nanna et al article above) and a brief search suggests there has been previous qualitative work on this area in type 2 diabetes which may be relevant - for example there appears to be work around ethnicity and health beliefs relating to insulin initiation that is directly relevant to the findings here (e.g. PMID: 27574375 and references within). The substantial increased use of the new more expensive glucose lowering agents in later stages in those of non white ethnicity may be relevant to this.*

Thank you very much for this suggestion and the link to the relevant work. We agree that including a discussion of the role of health beliefs in ethnic differences in treatment is relevant and will greatly strengthen the relevance of our paper. We have now added extra text on this topic this to the discussion section.

“Ethnic differences in health beliefs and attitudes towards medication may also play a role in therapeutic inertia. A recent study from the USA found that statin undertreatment amongst African American groups was partially explained by lower levels of trust in healthcare practitioners and lower perceived safety of statins in the African American population compared to the white majority population. [36] Similarly, a UK survey of the Bangladeshi population found that refusal of insulin treatment was associated with fear of premature death, fear of weight gain, loss of independence, and lack of perceived improvements to quality of life.[34]”

*Additional points

1. It is a little confusing exactly what has been adjusted for, and why. Baseline covariates (page 6) suggests factors like smoking and BMI were included as covariates in analysis, but many of these are absent from the related statement of a-priori confounders on page 8.* Page *8 suggests to my mind that consultations and number of measurements were also adjusted for, but this does not appear to be the case in table 2. In table 3 all the things on page 6 again crop up. This leaves me rather lost, and also confused why different covariates may be considered (if indeed this is the case) for very closely related analysis of time to intensification of therapy and treatment inertia, which would appear to be aspect of the same thing. Related to this the DAG suggests the authors consider there is a similar relationships for hbA1c and BMI/smoking etc but (if page 8/table 2 is correct) these appear to be treated differently in analysis. One would expect BMI in particularly to affect
choice of agent (given clinicians and patients may be more reluctant to use medications associated with weight gain such as insulin/sulfonylureas where there is obesity).*

Thank you very much for this comment. We had originally set out to look at a-priori and fully adjusted models, however since these models yielded very similar results - the models presented in the paper did not end up being completely aligned.

We agree with the reviewer that all of the covariates available for this study are relevant to both the analyses of time to initiation/intensification, and odds of therapeutic inertia. With this in mind we have re-run the models for both sets of analyses so that they both account for exactly the same set of confounders.

Namely: HbA1c value, BMI value, gender, deprivation, age at diagnosis, macro- and micro-vascular disease, calendar year, smoking status, BMI, number of consultations, and depression (as requested by reviewer 2).

Due to the high collinearity between number of consultations and number of HbA1c measurements (coeff=0.8), it was decided to adjust only for number of consultations in the models.

The direction of the associations and interpretation of the results remains unchanged – however, we thank the reviewer for improving the consistency of our methodology and reporting for the manuscript- which will undoubtedly improve understanding and clarity for the reader.

*2. There is to my mind a possibility of residual confounding here that may be exacerbated by the use of adjustment for artificial subgroups (as the covariates description on page 6 suggests) rather than using continuous covariates. Was there a reason to split age into deciles rather than adjust for age as a continuous covariate (in the same way as other groups using this dataset have done?), ditto for BMI which is even more crudely categorised - a BMI of 30 and 60 are likely to have different influences on choice of treatment.*

Thank you for this comment. Age was considered as a continuous covariate in the survival analysis models. In order to be consistent throughout the paper, we have now updated the analysis of treatment inertia to also consider age as a continuous variable. We have now updated the description of the age variable in the methods section to make this clear.

BMI was originally categorised to account for previously published evidence around the differing levels of vascular/diabetes risk conferred in different ethnic groups at the same BMI. We have now updated the analyses of therapeutic inertia to account for BMI and HbA1c as continuous variables in the same way as age.

We agree that categorisation of continuous variables may result in us losing information by creating artificial groupings. The new analyses now consider HbA1c, BMI and age as continuous variables throughout.

*3. We know there is marked regional variation in use of newer agents and potentially rural/urban differences in care. There will also be major differences in ethnicity by rural/urban status and region. I assume this will be accounted for by modelling at the practice level, could the authors reassure me that this is the case?*

This is correct, all regression models accounted for clustering at the practice level by using multilevel techniques to account for the clustering of patients within practices. This is mentioned in the methods and discussion sections.

Methods: “As individuals attending the same general practice may have similar levels of care provision and clinical coding, multilevel modelling was used to account for the clustering of people within practices.”

References

1	NHS Commissioning Assembly. The Diabetes Sample Service Specification. 2014. https://www.diabetes.org.uk/Professionals/Position-statements-reports/Integrated-diabetes-care

2	Mathur R, Palla L, Chaturvedi N, *et al.* Ethnic differences in the severity and clinical management of type 2 diabetes at time of diagnosis: A cohort study in the Clinical Practice Research Datalink. *Diabetes Res Clin Pract* 2020;**160**:108006. doi:10.1016/j.diabres.2020.108006

3	Bartlett JW, Harel O, Carpenter JR. Asymptotically Unbiased Estimation of Exposure Odds Ratios in Complete Records Logistic Regression. *Am J Epidemiol* Published Online First: 2014. doi:10.1093/aje/kwv114

4	Visram H. Patient Barriers to Insulin Use in Multi-Ethnic Populations. *Can J Diabetes* Published Online First: 2013. doi:10.1016/j.jcjd.2013.02.054

5	Khan H, Lasker SS, Chowdhury TA, *et al.* Prevalence and reasons for insulin refusal in Bangladeshi patients with poorly controlled Type 2 diabetes in East London. *Diabet Med* 2008;**25**:1108–11.http://ovidsp.ovid.com/ovidweb.cgi?T=JS&PAGE=reference&D=emed8&NEWS=N&AN=2008413581

6	Patel N, Stone MA, Chauhan A, *et al.* Insulin initiation and management in people with Type2 diabetes in an ethnically diverse population: The healthcare provider perspective. *Diabet Med* Published Online First: 2012. doi:10.1111/j.1464-5491.2012.03669.x

7	Dixon DL, Sharma G, Sandesara PB, *et al.* Therapeutic Inertia in Cardiovascular Disease Prevention: Time to Move the Bar. *J Am Coll Cardiol* Published Online First: 2019. doi:10.1016/j.jacc.2019.08.014

8	Khunti K, Kosiborod M, Ray KK. Legacy benefits of blood glucose, blood pressure and lipid control in individuals with diabetes and cardiovascular disease: Time to overcome multifactorial therapeutic inertia? Diabetes, Obes. Metab. 2018;**20**:1337–41. doi:10.1111/dom.13243
