# Supplementary material for: Ethnic disparities in initiation and intensification of diabetes treatment in adults with type 2 diabetes in the UK, 1990–2017: A cohort study
Source: PLoS Med. 2020 May 15;17(5):e1003106. doi: 10.1371/journal.pmed.1003106 (PMC7228040; doi:10.1371/journal.pmed.1003106)
Supplement: Attachment — Submitted filename: Response_to_reviewers_6032020.docx [file pmed.1003106.s015.docx]

Dear Dr. Turner,

Thank you for taking the time to send our manuscript for review again. We are happy that the paper has been accepted for publication pending our response to the outstanding queries. These are addressed below, with our responses in red. A marked revision of the manuscript also has the changes in red font.

Requests from Editors:

We suggest a more compact title: "Ethnic disparities in initiation and intensification of diabetes treatment in adults with type 2 diabetes in the UK, 2004-2017: a cohort study"

Thank you for this suggestion- we are happy to amend the title as you have suggested

Please add a full point as needed after "hypothesized confounders" in the abstract.

This has now been added

Please adapt "... groups were faster to initiate ..." (abstract) to "... non-insulin monotherapy was initiated earlier in south Asian and Black groups ..." or similar. 
We have adapted the sentence as suggested

In the "Conclusions" subsection of your abstract, please remove the word "strong", bearing in mind the research design, or substitute "persuasive" or similar.

This has now been changed

At the end of the "Introduction" section of your main text, are (b) and (c) different? Please reword as appropriate.

We have clarified that (b) refers to time to initiation/intensification (survival analysis) while (c) refers to treatment inertia following identification of uncontrolled HbA1c. While the former point refers to hazard ratios comparing time to event, the latter point refers to ethnic differences in the odds of intensifying treatment within 12 months of having an uncontrolled HbA1c recorded.

Please refer to the attached "RECORD" checklist at the appropriate point in your methods section.

We have added a reference to the attached RECORD checklist in the methods section just before the ‘secondary analysis’ subsection.

Please remove the short section on "patient and public involvement".

This is now removed.

Under "ethnic differences" (results, third paragraph), please correct "p<0.001".

This has now been corrected.

In the first paragraph of the discussion, please adapt "... experienced significant delays" to "were more likely to experience delays" or similar. 
This has now been changed.

Please avoid the phrase "white majority".

This has now been removed throughout the manuscript. 

Please use the term "diabetes treatment", or similar, rather than "antidiabetic therapy" throughout the ms. 
This has now been changed throughout the manuscript. 

Please substitute "sex" for "gender" throughout the article. 
This has now been changed throughout the manuscript. 

Please reposition all reference call-outs as follows: "... adverse diabetes outcomes [12,13]. In the UK ...".

These have now been changed throughout the manuscript. 


In the reference list, please format author names correctly where needed, e.g., references 32, 28 and 40.

The formatting of all references has been checked and fixed.

Please add journal names where they are missing, e.g., references 18 and 44.

The journal names have now been added.

In table 1, please remove the colour highlighting of "depression".

This has been removed- it was highlighted in the previous version as part of the marked copy to indicate changes requested by the reviewer.

FIGURES and GRAPHICS: 

1. Please ensure that all main figures are uploaded in .tif or .eps format. For more information about how to convert your figure files, please see our guidelines:[http://journals.plos.org/plosmedicine/s/figures](https://protect-eu.mimecast.com/s/VFmPC4LO0C2LQ7iMUrBK?domain=journals.plos.org).
Figures are now in Tiff format.

2. Please ensure that all main figure files are cited in ascending numerical order in the main text of the article.

The figure files are now cited in ascending order

3. Please ensure that all main figures are referenced in the manuscript as Fig 1, Fig 2, etc. (including capitalization), rather than Figure 1, Figure 2, etc. Please note, however, that the file names themselves must not include the space, i.e. Fig1.tif, Fig2.tif, etc.
Figures are referenced as Fig 1, file names are Fig1.tif etc.

4. Please ensure that all main figure legends are placed after the paragraph containing their first in-text citation.

Figure legends are now added to text following their first citation


TABLES and BOXES:

1. Please cite main tables in ascending numerical order in your main article file.

Tables are now cited in ascending order

2. Please ensure that all main tables in their entirety (including titles and footnotes), are placed after the paragraph containing their first in-text citation.

All tables are now incorporated into the main manuscript.


METADATA:

1. Please ensure that the CRediT author contributions listed for each co-author are completed in full. To qualify for authorship, each author must meet at least one of the seven core contributions (conceptualization; data curation; formal analysis; investigation; methodology; software; validation), as well as at least one of the writing contributions (original draft preparation; review and editing). Authors may also be noted against any of the remaining contributions, however these alone will not qualify a contributor for authorship.

The currently listed contributions of the following authors do not qualify them for authorship according to the above criteria: Sophie Eastwood and Nish Chaturvedi. Please ensure that the full contributions of each author are acknowledged in the "Add/Edit/Remove Authors" section of our submission form. If they do not meet criteria for authorship please let us know as soon as possible by emailing [plosmedicine@plos.org](mailto:plosmedicine@plos.org).

Can I add the following contributions:

Sophie Eastwood: Methodology, review and editing

Nish Chaturvedi: Conceptualization, review and editing

2. In accordance with PLOS' data policy, please ensure that your Data Availability Statement in the submission form clearly identifies how readers can access your data. Data cannot be available on request, or only accessible by contacting one of the authors. Note that your Data Availability Statement will be typeset as it is written, so please ensure it is in complete sentences and appears as you would like it to in the published version.

For details on our data policy, please see [http://journals.plos.org/plosmedicine/s/data-availability](https://protect-eu.mimecast.com/s/siTVC5LPGCPqQWTNNhs2?domain=journals.plos.org)
.

This is added

ARTICLE FILE:

1. Please indicate the corresponding author in the byline by placing an asterisk (*) after their affiliation number.

2. Please include the corresponding author's email address on the title page of your manuscript, indicated by an asterisk (*). Only an asterisk and the email address itself are required.

These are now added

3. Please remove the funding information from your manuscript file. The published version is pulled automatically from the “Financial Disclosure” field in the submission form and must not be duplicated. Please therefore take this opportunity to check that the information in this field is correct and completed in full.
The funding statement has been removed.

4. Please remove the Competing Interests section from your manuscript file. The published version is pulled automatically from the "Competing Interests" field in the submission form and must not be duplicated. Please therefore take this opportunity to check that the information in this field is correct and completed in full.

Competing interests are removed.

5. Please remove your Author Contributions section from your manuscript file as this information is automatically pulled from the authors section of our submission form and must not be duplicated. Please therefore take this opportunity to check that the information in this field is correct and completed in full.

Author contributions are removed.

6. Please remove the data availability information from your manuscript file as this is automatically pulled from the "Data Availability" field in the submission form and must not be duplicated. Please therefore take this opportunity to check that the information in this field is correct and completed in full.
Data availability statement removed.

REFERENCES: 

1. Please ensure your references are in the style of PLOS Medicine (ICMJE/Vancouver style). The reference style guide can be found here:[http://journals.plos.org/plosmedicine/s/submission-guidelines#loc-references](https://protect-eu.mimecast.com/s/tNrPC69QJS6QAGt2XsxM?domain=journals.plos.org). You can also find a downloadable EndNote file in these guidelines to assist you. Please do a manual check as well, as the EndNote file does not catch everything. Please note that the references will be typeset as they appear so please ensure the format is consistent.

All references are in the Mendeley Style ‘PLOS Medicine’

2. Please put all reference citations in square brackets separated by either commas or dashes with no spaces, e.g. [1,2,3] or [1-3].

Done.

3. Please make sure all references are cited in ascending numerical order in the text. 
Done.

SUPPORTING INFORMATION: 

1. Please upload your supporting information as individual files, and use the following naming format: S1 Text; S1 Fig; S1 Table, S1 Data, etc. Each file type should start as S1 and increase numerically: S1 Text, S2 Text, etc. Note that all relevant in-text citations and captions will need to be updated accordingly.

The supporting information has been separated into individual files and renamed in the main manuscript as required.

2. Your RECORD checklist is currently listed as file type ‘Other’ in the system’s file inventory, meaning it will not publish with the final article. If you would like this document to be published, please change the file type to 'Supporting Information' and ensure it adheres to our naming conventions.

Done.

3. Please ensure that all SuppoS6_TableS8_TaS11rting Information files use the following format verbatim throughout the manuscript (including legends and all in-text citations): S1 Fig, S1 Table, S1 Text, S1 Data, etc. Each file type should start at 1 and increase numerically. Please note, however, that the file names themselves must use an underscore rather than a space, i.e. S1_Fig.tif, S1_Table.xlsx, S1_Text.doc, S1_Data.xlsx, S1_RECORD_Checklist.docx, etc.
Done.

4. Please ensure that each supporting information file has a legend at the end of the manuscript file, after the Reference list.

Done.

5. Please note that your submission cannot contain any references to generic "supporting information" files and instead must refer to specific files using our naming convention (S1 Fig, S1 Table, S1 Text, S1 Data, SI PRISMA Checklist, etc.)

Done.

please also update the author lists for references 38 and 40:

Ref 38 author list should be: "Khan H, Lasker SS, Chowdhury TA."

Ref 40 author list should be: "Sinclair AJ, Bayer AJ, Girling AJ, Woodhouse KW."

 These are both updated.

Comments from Reviewers:

*** Reviewer #1: 

Thank you for the revision and replies to the comments made on the original submission. Overall I consider my original comments to be resolved with the changes to the manuscript in this version and the inclusion of the protocol in the supplementary materials. The change to use continuous covariates (i.e. BMI) as suggested by reviewer 3 is a good change to the manuscript and the presentations of hazard/odds ratios in Table 2/3 highlights the differences between groups well. I had one query relating to the identification of ethnicity prompted by the additional table, and a comment about the figures in the main part of the paper. Table S1 is very helpful to understand the selection of ethnic groups as the main exposure in the study. The majority of persons haven an 'unknown' ethnicity classification and are excluded from the analysis, which would presumably mean that many of the persons who are actually 'White' are excluded at this this step.

1. Are the 'White' group very similar to those in the 'Unknown' category? i.e. are the differences seen between ethnicities in treatment intensification driven by selection bias on identification as 'White' in the CPRD? My own experiences with routinely-collected data on self-reported CoB suggest that people and health services that elicit this information from patients are likely to be different to those that do not.

Thank you for this comment- It is likely that the unknown ethnic group are largely white, and thus reasons for not having a valid ethnicity recorded (such as not being asked by the healthcare practitioner, or refusing to give the information when requested), are related to their beliefs, usage of services, and engagement with practitioners – and also that practitioners may not feel the need to ask certain people about their ethnicity.

We have now added a table in the supplementary materials comparing time to intensification for those of white, other, mixed, and unknown ethnicity and referred to it in the main text (Table S6). Time to initiation of non-insulin monotherapy is slower for those of unknown ethnicity compared to white (HR 0.89, 0.82-0.97, p=0.008). This contrasts with our finding of faster initiation amongst ethnic minority groups. However, the finding of slower intensification to insulin is comparable to our findings for ethnic minority groups (HR 0.89, 95%CI 0.82-0.96, p=0.002).

Given that the number of HbA1c measurements between stages is equivalent for those of unknown vs. white ethnicity, our findings suggest that poorer monitoring is not a driving factor of slower treatment change in this population as it we found to be the case for south Asian and black groups.

2. A table comparing the baseline characteristics of the excluded 'Other' with 'White would resolve whether a select group more likely to receive treatment intensification was used by only selecting the people with a confirmed 'White' ethnicity code.

Thank you for this suggestion- a full table 1 comparing people of White ethnicity to all other ethnic groups, including mixed/other and unknown is now included in the supplementary materials and referred to in the main text (Table S5). The table shows that baseline indicators fall between those of the white and south Asian/black groups – suggesting some heterogeneity in the composition of the unknown ethnic group.

We explored differences between those of known and unknown ethnicity in detail in our previous publication “Ethnic differences in the severity and clinical management of type 2 diabetes at time of diagnosis: A cohort study in the UK Clinical Practice Research Datalink, Diabetes Res Clin Pract. 2020” We found that individuals with unknown ethnicity were younger at diagnosis and less deprived than those of known ethnicity. Coupled with the findings of heterogeneity in clinical profile at diagnosis (poorer risk factor levels but fewer co-morbidities), we surmised that this is a mixed group encompassing younger, healthier, and more affluent individuals who may not need to access healthcare, and individuals who are less healthy, or less able to access care.

2. Prior to publication, the Figures 2 and 3 should be revised to a higher standard: 1) there is not main axis title, 2) The numbers in Figure 2 are difficult to read against the striped background, and in the rarer intensification patterns they overlap and can't be read, 3) The table in Figure 3 needs some revision so it's clear there are 5 drug reported across the axis (darker gridlines between drug numbers?)

Thank you for this comment-

For figure 2, an axis title has been added. The striped pattern has been changed to solid and a new graph layout has been chosen to better illustrate the difference in intensification patterns. The data labels have been moved so they are easier to read. For figure 3 it is not possible to manipulate the gridlines so that certain ones are darker than others.

*** Reviewer #2: 

The authors have properly addressed the issues raised by this reviewer

Thank you for reviewing our manuscript. We are glad our responses to your previous comments were satisfactory.

*** Reviewer #3: 

Thank you for fully addressing my comments. I have only one further comment (for information): while there are clear limitations in assessing adherence in CPRD it is possible to assess whether a patient is requesting sufficient repeat prescriptions to cover the prescribed dose, and this measure has been shown to be associated with glycaemic control in CPRD - see Farmer et al Diabetes Care 2016 PMID: 26681714.

Thank you for your comment- other members of our group are currently researching associations between medication dosage and adherence, and impacts on risk factor levels.
